# Labdane-Type Diterpenoids from *Streptomyces griseorubens* and Their Antimicrobial and Cytotoxic Activities

**DOI:** 10.3390/ijms25063311

**Published:** 2024-03-14

**Authors:** Chang-Su Heo, Jong Soon Kang, Jeong-Wook Yang, Min Ah Lee, Hwa-Sun Lee, Hee Jae Shin

**Affiliations:** 1 Marine Natural Products Chemistry Laboratory, Korea Institute of Ocean Science and Technology, 385 Haeyang-ro, Yeongdo-gu, Busan 49111, Republic of Korea; science30@kiost.ac.kr (C.-S.H.); minah@kiost.ac.kr (M.A.L.); hwasunlee@kiost.ac.kr (H.-S.L.); 2 Department of Marine Technology and Convergence Engineering, Korea National University of Science and Technology (UST), 217 Gajungro, Yuseong-gu, Daejeon 34113, Republic of Korea; 3 Laboratory Animal Resource Center, Korea Research Institute of Bioscience and Biotechnology, 30 Yeongudanjiro, Cheongju 28116, Republic of Korea; kanjon@kribb.re.kr (J.S.K.); z7v8@kribb.re.kr (J.-W.Y.); 4 Department of Chemistry, Pukyong National University, 45 Yongso-ro, Nam-gu, Busan 48513, Republic of Korea

**Keywords:** *Streptomyces griseorubens*, labdane, diterpenoids, antimicrobial, cytotoxicity, chlorolabdan, epoxylabdan

## Abstract

Chemical investigation of the ethyl acetate (EtOAc) extract from a marine-derived actinomycete, *Streptomyces griseorubens*, resulted in the discovery of five new labdane-type diterpenoids: chlorolabdans A-C (**1**–**3**), epoxylabdans A and B (**4** and **5**), along with one known analog (**6**). The structures of the new compounds were determined by spectroscopic analysis (HR-ESIMS, 1D, and 2D NMR) and by comparing their experimental data with those in the literature. The new compounds were evaluated for their antimicrobial activity, and **2** displayed significant activity against Gram-positive bacteria, with minimum inhibitory concentration (MIC) values ranging from 4 to 8 µg/mL. Additionally, **1**, **2**, and **4** were tested for their cytotoxicity against seven blood cancer cell lines by CellTiter-Glo (CTG) assay and six solid cancer cell lines by sulforhodamine B (SRB) assay; **1**, **2**, and **4** exhibited cytotoxic activities against some blood cancer cell lines, with concentration causing 50% cell growth inhibition (IC_50_) values ranging from 1.2 to 22.5 µM.

## 1. Introduction

Labdane-type diterpenoids are one of the most abundant categories of secondary metabolites, which are discovered in plants [1,2], fungi [3,4], and marine organisms [5,6]. The labdane derivatives were isolated mainly from plant materials in families such as the gymnospers, as well as the Asteraceae, Lamiaceae, and Zingiberaceae [7,8], and sometimes they were discovered in actinomycetes [9]. Terpenoids commonly function as growth or defense materials for organisms that produce them [8]. For these reasons, some terpenoids are known to be toxic or allergenic and smell like anthelmintics [8]. Sclareol is a representative example of labdane-type diterpenoids (Labd-14-ene-8,13-diol). It is found in *Salvia sclarea* and used as an additive in the flavor and fragrance industries [10]. The naturally occurring labdane comprises four isoprene units, and its structural scaffolds typically consist of a decalin system fused with two rings and an attached side chain composed of six carbons [11]. They have *trans*-stereochemistry mainly at C-5 and C-10 carbon junctions of the ring, and lapdanes with *cis*-ring junctions are rarely found in nature [12]. Up to now, various labdane derivatives have been reported [5,13,14,15] which possess additional carbons on the decalin system. Labdanes have been acknowledged as an important source for exploring new therapeutic agents [16] with a wide range of bioactivities, such as antibacterial [2], anticancer [17], anti-inflammatory [12], antioxidant [3], and antiviral [18] properties, and they are still regarded as good subjects for research.

**Figure 1:**
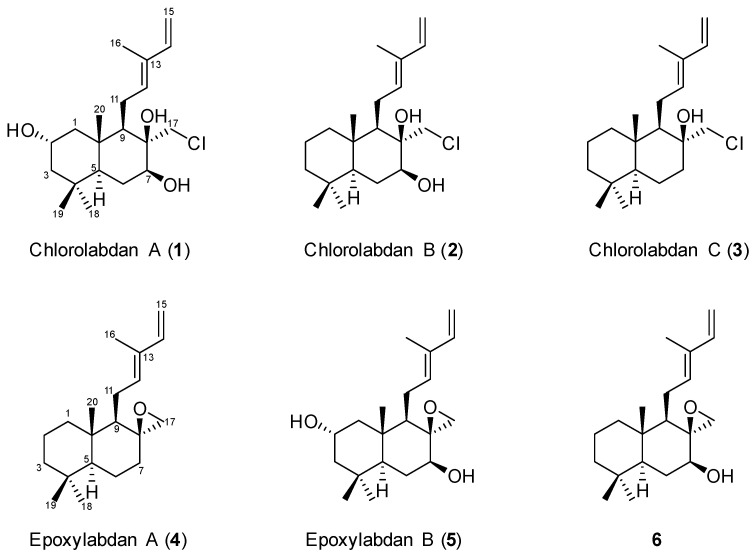
Structures of **1**–**6** isolated from *S. griseorubens* 2210JJ-087.

**Figure 2:**
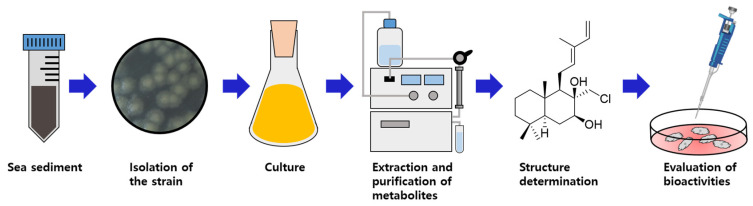
Schematic diagram of experimental procedures.

The use of microorganisms as a source of secondary metabolites has advantages, such as no climatic and spatial limit, in contrast with plant cultures [19]. The genus *Streptomyces* is especially recognized as the most significant producer of secondary metabolites, and up to 50% of the total population originated from the soil, including sediments [20]. As part of our continuous search for new secondary metabolites from marine microorganisms, an actinobacterial strain was isolated from a sediment sample collected at Jeju Island, Republic of Korea. The strain was identified as *Streptomyces griseorubens* 2210JJ-087 by 16S gene sequence analysis. The crude extract from the culture broth of the strain exhibited antibacterial activity against Gram-positive bacteria. Therefore, an additional study was carried out to identify bioactive secondary metabolites produced by the strain. As a result, six labdane diterpenoids, including five new (**1**–**5**) and one known (**6**) derivative (Figure 1 and Figure S1), were isolated from the strain. This report describes the isolation, purification, structure determination, and evaluation of the antimicrobial and cytotoxic activities of the compounds (Figure 2).

## 2. Results and Discussion

Compound **1** was isolated as a light green powder. Its molecular formula was determined as C_20_H_33_ClO_3_ based on a high-resolution electrospray ionization mass spectrometry (HRESIMS) peak at *m*/*z* 379.2012 [M+Na]^+^ (calcd for C_20_H_33_ClO_3_Na, 379.2010). The isotopic pattern at *m*/*z* 381.1996 [M+Na]^+^, with a ratio of 3:1, suggested the presence of a chlorine atom in the molecule (Figure S2). The ^1^H NMR data (Table 1 and Figure S3) showed the signals of four olefinic protons at *δ*_H_ 6.33 (^1^H, dd, *J* = 17.4, 10.8 Hz, H-14), 5.58 (^1^H, t, *J* = 6.9 Hz, H-12), 5.06 (^1^H, dd, H-15b), and 4.87 (^1^H, overlapped, H-15a); two oxymethines at *δ*_H_ 3.82 (^1^H, m, H-2) and 3.81 (^1^H, overlapped, H-7); ten methylenes at *δ*_H_ 0.81–3.60; two methines at *δ*_H_ 0.88 (^1^H, dd, *J* = 12.4, 1.8 Hz, H-5) and 1.50 (^1^H, dd, *J* = 6.3, 2.5 Hz, H-9); and four methyl groups at *δ*_H_ 0.93–1.79. The ^13^C NMR data (Table 1 and Figure S4) and heteronuclear single quantum coherence (HSQC) NMR data revealed the presence of four olefinic carbons at *δ*_C_ 142.9 (C-14), 136.5 (C-12), 133.8 (C-13), and 110.4 (C-15); one oxygenated quaternary carbon at *δ*_C_ 77.9 (C-8); two oxymethines at *δ*_C_ 71.0 (C-7) and 65.1 (C-2); two methines at *δ*_C_ 53.1 (C-5) and 53.0 (C-9); two quaternary carbons at *δ*_C_ 41.2 (C-10) and 35.5 (C-4); five methylenes at *δ*_C_ 24.2–51.6; and four methyls at *δ*_C_ 12.0–34.1 (Figure S5). 

**Figure 3:**
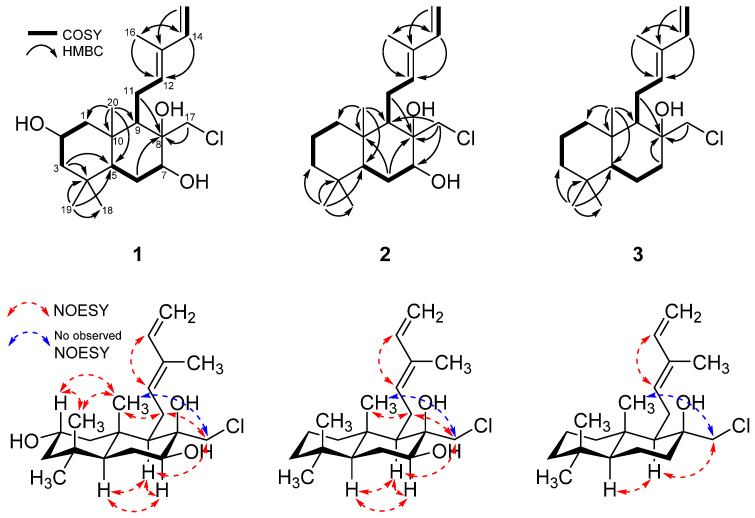
Key COSY, HMBC, and NOESY correlations of **1**–**3**.

The planar structure of **1** was elucidated by analyses of ^1^H-^1^H correlation spectroscopy (COSY) and heteronuclear multiple bond correlation (HMBC) NMR data (Figure 3, Figures S6 and S7). The ^1^H-^1^H COSY spectrum exhibited four spin systems: H_2_-1/H-2/H_2_-3, H-5/H_2_-6/H-7, H-9/H_2_-11/H-12, and H-14/H_2_-15. The HMBC correlations, from H_2_-3 to C-4 and C-5, and from H_3_-20 to C-1, C-5, and C-10, confirmed the connectivity of ring A. In addition, the HMBC correlations of H_2_-6/C-8, H_2_-11b/C-8, and H_3_-20/C-9 elucidated a decalin system of ring B, fused with ring A at C-5 and C-10. The HMBC correlations, from H-14 to C-12 and C-16, and from H_3_-16 to C-12 and C-14, identified a partial structure of the chain from C-12 to C-16 with olefins and the connection of ring B. Further, the HMBC correlations from H_3_-17 to C-8, with downfield-shifted carbon at C-17 (*δ*_C_ 45.6), confirmed the location of the chlorine atom. Thus, the gross structure of **1** was established, as shown in Figure 3.

**Figure 4:**
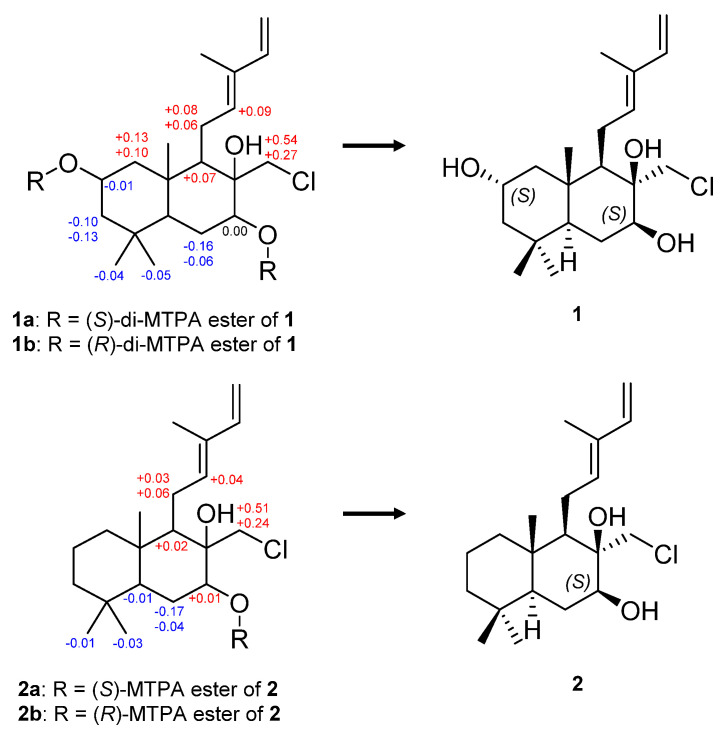
*Δδ_H_* values (in ppm) = *δ_S_* − *δ_R_* obtained for (*S*)- and (*R*)-MTPA esters of **1** and **2**.

The relative configuration of **1** was elucidated by the analysis of nuclear Overhauser effect spectroscopy (NOESY) data (Figure 3 and Figure S8). The strong NOESY correlation of H-12 with H-14 confirmed the *E*-configuration of *∆*^12^. The NOESY correlations of H-9 with H-5 and H-7, and between H-5 and H-7, indicated these protons have an α-orientation, while the NOESY correlations of H-20 with H-2, H-11a, and H-18, and between H-2 and H-18 suggested the *β*-orientation of these protons. The NOESY correlation of H-17a with H-9 and H-11b and the lack of NOESY cross-peaks between H-17 and H-20 indicated that H-17 has an *α*-orientation. Additionally, these assignments were verified by previous research on the total synthesis of cyslabdan [21]. Therefore, the relative configuration of the chiral centers at C-2, C-5, C-7, C-8, C-9, and C-10 were determined as 2S*, 5S*, 7S*, 8R*, 9R*, and 10S*. The absolute configuration of **1** was established by the modified Mosher’s method. Comparing ^1^H NMR data of (*S*)- and (*R*)-di-MTPA esters of **1** (**1a** and **1b**, Figure 4 and Figures S9–S15), the absolute configuration of the chiral centers at C-2 and C-7 was determined as 2*S*, 7*S*. Consequently, the absolute configuration of **1** was confirmed as 2*S*, 5*S*, 7*S*, 8*R*, 9*R*, and 10*S*, and **1** was named chlorolabdan A.

Compound **2** was isolated as a light green powder. Its molecular formula of C_20_H_33_ClO_2_ was determined based on an HRESIMS peak at *m*/*z* 363.2057 [M+Na]^+^ (calcd for C_20_H_33_ClO_2_Na, 363.2061), and its isotopic peak at *m*/*z* 365.2044 [M+Na]^+^, with a ratio of 3:1, revealed the presence of a chlorine atom (Figure S16). The ^1^H and ^13^C NMR spectra of **2** (Figures S17 and S18) were almost consistent with those of **1**, though the chemical shifts of **2** at C-2 (*δ*_H_ 1.42/*δ*_C_ 19.4 for **2** and *δ*_H_ 3.82/*δ*_C_ 65.1 for **1**) were significantly different from those of **1**. Accordingly, a methylene was assigned at C-2 in **2** instead of the oxymethine in **1**. By detailed analysis of the HSQC, COSY, and HMBC data of **2** (Figures S19–S21), the planar structure of **2** was also elucidated, as shown in Figure 3. The interpretation of NOESY data (Figure S22) indicated that the relative configuration of **2** was similar to that of **1**. The absolute configuration of **2** was elucidated by the modified Mosher’s method. By comparing ^1^H NMR data of (*S*)- and (*R*)-MTPA esters of **2** (**2a** and **2b**, Figure 4 and Figures S23–S29), the absolute configuration of **2** was determined as 5*S*, 7*S*, 8*R*, 9*R*, and 10*S*, and **2** was given the name of chlorolabdan B.

Compound **3** was isolated as a colorless solid. Its molecular formula of C_20_H_33_ClO was determined based on an HRESIMS peak at *m*/*z* 325.2277 [M+H]^+^ (calcd for C_20_H_34_ClO, 325.2293), requiring four degrees of unsaturation. The isotopic pattern at *m*/*z* 327.2480 [M+H]^+^, with a ratio of 3:1, suggested the presence of a chlorine atom (Figure S30). The ^1^H and ^13^C NMR data of **3** (Figures S31 and S32) were almost identical to those of **2**, except for the chemical shifts at C-7 (*δ*_H_ 1.71, 1.80/*δ*_C_ 37.9 for **3** and *δ*_H_ 3.79/*δ*_C_ 71.1 for **2**) indicating the oxymethine at C-7 of **2** was changed to a methylene in **3**. The planar structure of **3** was established by detailed analysis of the HSQC, COSY, and HMBC data (Figures S33–S35), and the relative structure of **3** was determined by the analysis of NOESY data (Figures S36 and S37). The absolute configuration of **3** was proposed by comparing the optical rotation value of **3** with those of **1** and **2** and those of the literature. The optical rotation value of **3** was ${\left[a\right]}_{D}^{25}$ +19.9 (*c* 0.1, MeOH), which was consistent with those of **1**, **2** (${\left[a\right]}_{D}^{25}$ +19.9 for **1** and +29.9 for **2**), and those of other analogs in the literature [21,22,23,24]. Consequently, the absolute configuration of **3** was determined as 5*S*, 8*R*, 9*R,* and 10*S*, and **3** was named chlorolabdan C.

**Figure 5:**
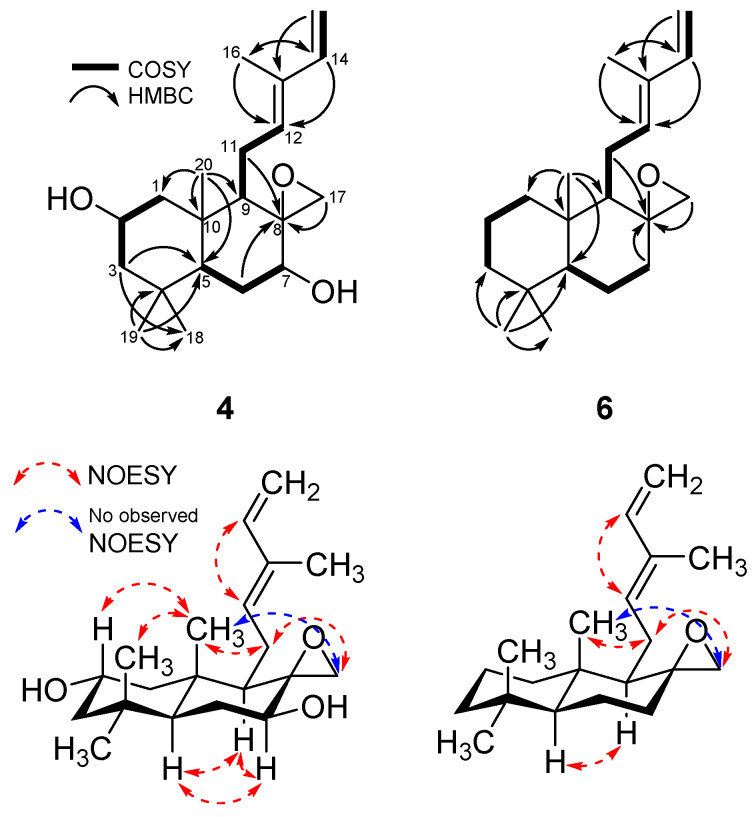
Key COSY, HMBC, and NOESY correlations of **4** and **5**.

Compound **4** was isolated as a colorless solid with a molecular formula of C_20_H_32_O by an HRESIMS peak at *m*/*z* 289.2533 [M+H]^+^ (calcd for C_20_H_33_O, 289.2526, Figure S38). An unsaturation number of five degrees was calculated for **4**, which is one more than **3**, suggesting the presence of one more ring in **4** compared to **3**. The ^1^H and ^13^C NMR data of **4** (Figures S39 and S40) were similar to those of **3**, except for the proton signals at C-17 (*δ*_H_ 2.26/2.56 for **4** and *δ*_H_ 3.23/3.38 for **3**). It was suggested that the chloromethylene at C-17 of **3** was replaced by an epoxy carbon in **4**, which was also supported by the HRESIMS data and unsaturation number. By the analysis of HSQC, COSY, HMBC, and NOESY data of **4** (Figures S41–S44), the planar and relative structures were established as shown in Figure 5. The absolute configuration of **4** was proposed as 5*S*, 7*S*, 8*R*, 9*R*, and 10*S* based on comparing the optical rotation value of **4** (${\left[a\right]}_{D}^{25}$ +16.6) with those of **1**–**3** (${\left[a\right]}_{D}^{25}$ +19.9 for **1**, +29.9 for **2**, and +19.9 for **3**). This assignment agreed with a previously reported derivative (**6**) in the literature [25], and **4** was named epoxylabdan A (Table 2).

Compound **5** was isolated as a colorless solid. Its molecular formula of C_20_H_32_O_3_ was determined based on an HRESIMS peak at *m*/*z* 343.2238 [M+Na]^+^ (calcd for C_20_H_32_O_3_Na, 343.2244, Figure S45). The interpretation of 1D and 2D NMR data (HSQC, COSY, and HMBC) revealed that the chloromethylene at C-17 of **1** was changed to an epoxy carbon in **5**, as shown in Figure 5 and Figure S46–S51. The relative configuration of **5** was consistent with those of **1**–**4** based on NOSEY data. The absolute configuration of **5** was also assigned as 2*S*, 5*S*, 8*R*, 9*R*, and 10*S* based on comparing the optical rotation value of **5** (${\left[a\right]}_{D}^{25}$ +26.4) with those of **1** and **4** (${\left[a\right]}_{D}^{25}$ +19.9 for **1** and +16.6 for **4**), and **5** was given the name of epoxylabdan B.

### Bioactivities

Compounds **1**–**6** were evaluated for their antimicrobial properties against three Gram-positive bacteria, *Bacillus subtilis* (KCTC 1021), *Micrococcus luteus* (KCTC 1915), and *Staphylococcus aureus* (KCTC 1927), and three Gram-negative bacteria, *Escherichia coli* (KCTC 2441), *Salmonella enterica* serovar Typhimurium (KCTC 2515), and *Klebsiella pneumoniae* (KCTC 2690). In previous studies, the labdane derivatives showed antibacterial activities against various pathogens, including methicillin-resistant *Staphylococcus aureus* (MRSA), *Bacillus subtilis*, *Staphylococcus aureus*, *Escherichia coli*, and *Micrococcus luteus* [23,26,27], and some of the strains were selected for the antimicrobial test to compare antibacterial activities of **1**–**6** with known compounds. As a result, **2** displayed weak MIC values against Gram-positive bacteria ranging from 4 to 8 µg/mL (Table 3), and other compounds did not significantly show an inhibition of the growth. Additionally, none of the compounds exhibited any activity against Gram-negative bacteria. Comparing our results with those of the previous studies [23,26,27], the MIC values of the new compounds were not significantly different from those of the previous ones. It is noteworthy that the new labdanes have chloromethylene moiety at C-8 (**1**–**3**). However, the chlorine atom may not serve a critical role in their activities.

Due to the limited amount of samples, only compounds **1**, **2**, and **4** were evaluated for their cytotoxicity against a normal cell line (RPMI-1788, B lymphocytes) and against seven blood cancer cell lines which are the most common cancer types in Korea: HL-60 (acute myelogenous leukemia, AML), Raji (Burkitt’s lymphoma), WSU-DLCL2 (diffuse large B cell lymphoma, DLBCL), NALM6 C. G5 (B cell acute lymphocytic leukemia, B-ALL), K562 (chronic myelogenous leukemia, CML), RPMI-8402 (T cell acute lymphocytic leukemia, T-ALL), and U266 (multiple myeloma). The tested compounds displayed cytotoxic activity against some standard cell lines, with IC_50_ values ranging from 1.2 to 22.5 µM lines (Table 4 and Table S1), while the compounds did not show activities against K562, RPMI-8402, U266, and RPMI-1788 cell lines (IC_50_: >30 µM). Although the tested compounds showed weak anticancer activities compared with previous studies [28,29,30], it is interesting that **1** showed more selective activity than **2** and **4**.

Compounds **1**, **2**, and **4** were further evaluated for their cytotoxicity against six solid cancer cell lines and a normal cell line: ACHN (renal), MDA-MB-231 (breast), PC-3 (prostate), NUGC-3 (stomach), NCI-H23 (lung), HCT-15 (colon), and MRC-9 (lung fibroblast). However, none of the compounds were cytotoxic against the six solid cancer cell lines or the normal cell line.

## 3. Materials and Methods

### 3.1. General Experimental Procedures

The 1D and 2D NMR spectra were acquired using a Bruker 600 MHz spectrometer (Bruker BioSpin GmbH, Rheinstetten, Germany). UV-VIS spectra were measured with a Shimadzu UV-1650PC spectrophotometer (Shimadzu Corporation, Kyoto, Japan). IR spectra were measured using a JASCO FT/IR-4100 spectrophotometer (JASCO Corporation, Tokyo, Japan). High-resolution ESIMS data were obtained with a Sciex X500R Q-TOF spectrometer (Sciex, Framingham, MA, USA). Low-resolution ESIMS data were acquired using an ISQ EM mass spectrometer (Thermo Fisher Scientific Korea Ltd., Seoul, Republic of Korea). Optical rotations were measured using a Rudolph analytical Autopol III S2 polarimeter with a sodium D line at 589 nm and 10 mm path length (Rudolph Research Analytical, Hackettstown, NJ, USA). HPLC experiments were carried out using a BLS-Class pump (Teledyne SSI, Inc., State College, PA, USA) on an ODS column (YMC-Pack-ODS-A, 250 × 10 mm i.d, 5 µm, Kyoto, Japan) with a Shodex RI-201H refractive index detector (Shoko Scientific Co., Ltd., Yokohama, Japan).

### 3.2. Isolation and Identification of Strain 2210JJ-087

Marine sediment samples were collected at Sehwa Beach, Jeju Island, in the Republic of Korea during expeditions in October 2022. On the seashore, the sediments were put into sterile 50 mL conical tubes and kept at 5 °C while returning to the laboratory. Then, selective heating pretreatment was carried out to eliminate unwanted microorganisms. Collected sediment samples weighing 1 g were placed in a sterile plate and kept in an oven at 60 °C for 30 min. After the heating pretreatment, 0.1 g of sediment was serially diluted to 10^−1^, 10^−2^, and 10^−3^ by sterile seawater, and each aliquot (50 µL) was spread on Bennett’s (BN) agar, Actinomycetes Isolation Agar (AIA), and Humic acid vitamin (HV) agar. The plates were stored in a BOD (Bio-Oxygen Demand) incubator at 28 °C for 7~28 days until colonies were visible. After incubation, the selected colonies were transferred onto new BN agar plates. Purification was carried out several times until single pure colonies could be seen. Strain 2210JJ-087 was isolated from HV agar and incubated for 7 days. The strain was identified as *Streptomyces griseorubens* based on morphological characteristics and 16S rRNA gene sequence analysis (GenBank accession number OR755843).

### 3.3. Fermentation of Strain 2210JJ-087, Extraction, and Isolation of Metabolites

The seed and mass cultures of strain 2210JJ-087 were carried out using the BN medium (1% glucose, 0.2% tryptone, 0.1% yeast extract, 0.1% beef extract, 0.5% glycerol, sea salts 32 g/L). A single colony of the strain from an agar plate was inoculated aseptically into a 100 mL conical flask filled with 50 mL of BN broth medium and incubated at 28 °C for 7 days on a rotary shaker at 140 rpm. The aliquot (25 mL) was inoculated aseptically into a 2 L flask containing 1 L of BN broth, and the strain was incubated at 28 °C for 7 days on a rotary shaker at 120 rpm. The seed culture broth was transferred to a 100 L fermenter filled with 70 L of BN broth and incubated at 28 °C for 14 days. The mass culture broth (70 L) was harvested and centrifuged at 60,000 rpm, and the supernatant was extracted twice with an equal volume of ethyl acetate (EtOAc, 70 L × 2). The EtOAc layer was evaporated to yield a crude extract (10.7 g). The crude extract was applied to ODS column chromatography followed by a stepwise gradient elution with methanol (MeOH) in H_2_O (1:4, 2:3, 3:2, 4:1, and 100:0, *v*/*v*). The fraction eluted with MeOH/H_2_O (4:1) was purified by a semi-preparative reversed-phase HPLC (YMC-Pack-ODS-A, 250 × 10 mm i.d, 5 µm, flow rate 2.0 mL/min, RI detector) using an isocratic elution of 60% MeOH in H_2_O to yield **5** (0.7 mg, *t*_R_ = 36 min) and **1** (2.4 mg, *t*_R_ = 56 min). The fraction eluted with MeOH/H_2_O (100:1) was purified by a semi-preparative reversed-phase HPLC using an isocratic elution of 85% MeOH in H_2_O to yield **6** (4.0 mg, *t*_R_ = 36 min), **2** (3.9 mg, *t*_R_ = 52 min), **3** (1.0 mg, *t*_R_ = 82 min), and **4** (1.4 mg, *t*_R_ = 98 min).

Chlorolabdan A (**1**): a light green powder; ${\left[a\right]}_{D}^{25}$ +19.9 (*c* 0.1, MeOH); UV (MeOH) *λ*_max_ (log *ε*) 232 (2.94) nm; IR *ν*_max_ 3430, 2827, 2355, 1642, 1436 cm^−1^; HRESIMS *m*/*z* 379.2012, [M+Na]^+^, calcd for [C_20_H_33_ClO_3_Na]^+^, 379.2010, *m*/*z* 343.2237, [M-HCl+Na]^+^, *m*/*z* 325.2278, [M-HCl-H_2_O+Na]^+^; for ^1^H NMR (CD_3_OD, 600 MHz), see Table 1; for ^13^C NMR (CD_3_OD, 150 MHz), see Table 1.

Chlorolabdan B (**2**): a light green powder; ${\left[a\right]}_{D}^{25}$ +29.9 (*c* 0.1, MeOH); UV (MeOH) *λ*_max_ (log *ε*) 232 (2.96) nm; IR *ν*_max_ 3434, 2837, 2365, 1647, 1426 cm^−1^; HRESIMS *m*/*z* 363.2057, [M+Na]^+^, calcd for C_20_H_33_ClO_2_Na 363.2061, *m*/*z* 327.2287, [M-HCl+Na]^+^; for ^1^H NMR (CD_3_OD, 600 MHz), see Table 1; for ^13^C NMR (CD_3_OD, 150 MHz), see Table 1.

Chlorolabdan C (**3**): a colorless solid; ${\left[a\right]}_{D}^{25}$ +19.9 (*c* 0.1, MeOH); UV (MeOH) *λ*_max_ (log *ε*) 232 (2.92) nm; IR *ν*_max_ 3433, 2907, 1597, 1438 cm^−1^; HRESIMS *m*/*z* 325.2277, [M+H]^+^, calcd for C_20_H_34_ClO 325.2293; for ^1^H NMR (CD_3_OD, 600 MHz), see Table 1; for ^13^C NMR (CD_3_OD, 150 MHz), see Table 1.

Epoxylabdan A (**4**): a colorless solid; ${\left[a\right]}_{D}^{25}$ +16.6 (*c* 0.1, MeOH); UV (MeOH) *λ*_max_ (log *ε*) 232 (2.82) nm; IR *ν*_max_ 3415, 2905, 2351, 1640, cm^−1^; HRESIMS *m*/*z* 289.2533, [M+H]^+^, calcd for C_20_H_33_O 289.2526; for ^1^H NMR (CD_3_OD, 600 MHz), see Table 1; for ^13^C NMR (CD_3_OD, 150 MHz), see Table 1.

Epoxylabdan B (**5**): a colorless solid; ${\left[a\right]}_{D}^{25}$ +26.4 (*c* 0.1, MeOH); UV (MeOH) *λ*_max_ (log *ε*) 232 (2.76) nm; IR *ν*_max_ 3430, 2893, 2350, 1642, 1136, cm^−1^ HRESIMS *m*/*z* 343.2238, [M+Na]^+^, calcd for C_20_H_32_O_3_Na 343.2244; for ^1^H NMR (CD_3_OD, 600 MHz), see Table 1; for ^13^C NMR (CD_3_OD, 150 MHz), see Table 1.

Compound **6**: a white powder; ${\left[a\right]}_{D}^{25}$ +23.3 (*c* 0.1, MeOH); UV (MeOH) *λ*_max_ (log *ε*) 232 (2.81) nm; IR *ν*_max_ 3412, 2923, 2363, 1618, 1163, cm^−1^; LRESIMS *m*/*z* 327.30, [M+Na]^+^, Figure S52; for ^1^H NMR (CD_3_OD, 600 MHz), see Figure S53; for ^13^C NMR (CD_3_OD, 150 MHz), see Figure S54.

### 3.4. Preparations of the MTPA Ester of **1** and **2** Using the Modified Mosher’s Method

(*R*)-MTPA chloride (5 µL) was added to a solution of **1** (0.2 mg) in anhydrous pyridine (0.5 mL) to obtain the (*S*)-di-MTPA ester of **1** (**1a**). The mixture was stirred at room temperature for 12 h. The mixture was dried under an N_2_ gas steam at 38 °C and extracted with EtOAc. The mixture was washed with 0.1 M HCl, 0.1 M NaHCO_3_, and 0.1 M NaCl solution. The mixture was evaporated, and ^1^H, HSQC, and COSY NMR spectra of **1a** were recorded to obtain the key ^1^H NMR data around stereocenters. Using the same procedure with (*S*)-MTPA chloride, (*R*)-di-MTPA ester of **1** (**1b**) was prepared, and (*R*)-MTPA ester of **2** (**2b**) and (*S*)-MTPA ester of **2** (**2a**) were also obtained.

(*S*)-di-MTPA esters of **1** (**1a**): *δ*_H_ 5.55 (H-12), 5.29 (H-2), 5.20 (H-7), 3.43 (H-17b), 3.33 (H-17a), 2.42 (H-11b), 2.23 (H-11a), 2.16 (H-1b), 1.98 (H-6b), 1.77 (H-3b), 1.70 (H-6a), 1.59 (H-9), 1.32 (H-3a), 1.09 (H-1a), 1.03 (H-19), 0.97 (H-18)

(*R*)-di-MTPA esters of **1** (**1b**): *δ*_H_ 5.46 (H-12), 5.30 (H-2), 5.20 (H-7), 3.16 (H-17b), 2.79 (H-17a), 2.36 (H-11b), 2.15 (H-11a), 2.06 (H-1b), 2.04 (H-6b), 1.90 (H-3b), 1.86 (H-6a), 1.52 (H-9), 1.42 (H-3a), 0.96 (H-1a), 1.07 (H-19), 1.02 (H-18) 

(*S*)-MTPA esters of **2** (**2a**): *δ*_H_ 5.54 (H-12), 5.20 (H-7), 3.37 (H-17b), 3.29 (H-17a), 2.45 (H-11b), 2.13 (H-11a), 1.96 (H-6b), 1.67 (H-6a), 1.48 (H-9), 1.04 (H-5), 0.96 (H-19), 0.86 (H-18)

(*R*)-MTPA esters of **2** (**2b**): *δ*_H_ 5.50 (H-12), 5.19 (H-7), 3.13 (H-17b), 2.78 (H-17a), 2.39 (H-11b), 2.10 (H-11a), 2.00 (H-6b), 1.84 (H-6a), 1.46 (H-9), 1.05 (H-5), 0.97 (H-19), 0.89 (H-18)

### 3.5. Antibacterial Assay

Antibacterial assay was conducted in a 96-well plate (SPL Life Sciences, Pocheon, Republic of Korea) by applying the broth microdilution method described by the Clinical and Laboratory Standards Institute [31]. Standard bacteria, KCTC 1021 (*Bacillus subtilis*), KCTC 1915 (*Micrococcus luteus*), KCTC 1927 (*Staphylococcus aureus*), KCTC 2441 (*Escherichia coli*), KCTC 2515 (*Salmonella enterica* serovar Typhimurium), and KCTC 2690 (*Klebsiella pneumoniae*), were purchased from Korean Collection for Type Cultures (KCTC, Daejon, Republic of Korea). The bacteria were incubated in Mueller Hinton Broth (MHB) for a day. Compounds (**1**–**6**) were dissolved in dimethyl sulfoxide (DMSO), and a serial twofold dilution of the compounds was prepared with MHB (100 µL) in the plate well in the range of 0.5–256 (µg/mL). After that, the broth containing the bacteria (100 µL) was added to each well, and the final concentration of bacteria was adjusted to 5 × 10^5^ CFU/mL by comparison with the McFarland standard. Growth-control well and sterility-control well were also prepared, and kanamycin was used as a positive control. The plates were incubated for 20 h at 37 °C, and MIC was determined by the lowest concentration at which the growth of bacteria was not visible.

### 3.6. CellTiter-Glo (CTG) and Sulforhodamine B (SRB) Assay for Cytotoxicity Testing

The cytotoxicity test was evaluated using CTG luminescent cell viability and SRB assay according to the published procedures [32,33]. Briefly, the blood cancer cell lines and a normal cell line were purchased from the American Type Culture Collection (Manassas, VA, USA) (HL-60, acute myelogenous leukemia, Raji: Burkitt’s lymphoma, NALM6 C. G5: B cell acute lymphocytic leukemia, K562: chronic myelogenous leukemia, U266: multiple myeloma, and RPMI-1788: B lymphocytes) and the DSMZ-German Collection of Microorganisms and Cell Cultures (WSU-DLCL2: diffuse large B cell lymphoma and RPMI-8402: T cell acute lymphocytic leukemia). The cell lines were incubated in RPMI 1640 supplemented with 10% fetal bovine serum, penicillin (100 IU/mL), and streptomycin (100 µg/mL) at 37 °C under a humidified atmosphere of 5% CO_2_, and the passage of cells was between 12 and 8. The cell lines were prepared in an opaque-walled 96-well plate (8 × 10^3^ cells/well), and compounds (**1**, **2**, **4**, and doxorubicin as positive control) with 0.1% DMSO were added to each well and incubated for 48 h. Then, the cell cultures were treated with 100 µL of CellTiter-Glo Reagent (Promega, Madison, WI, USA) and maintained for 10 min to obtain the luminescence signal. GloMax-Multi Detection System (Promega, Madison, WI, USA) was used for measuring. The solid cancer cell lines and the normal cell line were purchased from the American Type Culture Collection (Manassas, VA, USA) (ACHN: renal, MDA-MB-231: breast, PC-3: prostate, NCI-H23: lung, HCT-15: colon, and MRC-9: lung fibroblast) and the Japanese Cancer Research Resources Bank (JCRB) (NUGC-3: stomach). The cell lines were incubated in RPMI 1640 supplemented with 10% fetal bovine serum, penicillin (100 IU/mL), and streptomycin (100 µg/mL) at 37 °C under a humidified atmosphere of 5% CO_2_, and the passage of cells was between 12 and 18. Then, cells were seeded in the 96-well plate (8 × 10^3^ cells/well), and compounds (**1**, **2**, **4**, and adriamycin as positive control) with 0.1% DMSO were added to each well. After incubation for 48 h, the cell cultures were fixed using 50% trichloroacetic acid (50 µg/mL) and were dyed with 0.4% SRB in 1% acetic acid. Unbound dye was washed using 1% acetic acid, and protein-bound dye was collected with 10 mM Tris base (pH 10.5) to measure the optical density. Absorbance was measured at 540 nm with a VersaMax microplate reader (Molecular Devices, Sunnyvale, CA, USA). GraphPad Prism 4.0 (GraphPad, San Diego, CA, USA) was used to calculate IC_50_ value.

## 4. Conclusions

Six labdane-type diterpenoids were isolated from the marine-derived *Streptomyces griseorubens* 2210JJ-087, including five new compounds (**1**–**5**), namely, chlorolabdans A–C, along with epoxylabdans A–B. The gross structures of the new compounds were elucidated by detailed analysis of HR-ESIMS, 1D, and 2D NMR data. The absolute configurations of the compounds were determined by the modified Mosher’s method and comparing their optical rotation values with those of the co-isolated derivatives and other analogs in the literature. Among the new compounds, **2** exhibited weak antimicrobial activity against Gram-positive bacteria (*Bacillus subtilis*, *Micrococcus luteus*, and *Staphylococcus aureus*) with MIC values ranging from 4 to 8 µg/mL. Additionally, **1**, **2**, and **4** were evaluated for their cytotoxicity against seven blood cancer cell lines, six solid cancer cell lines, and two normal cell lines. The tested compounds displayed cytotoxicity against some blood cancer cell lines with IC_50_ values ranging from 1.2 to 22.5 µM, and **1** showed cytotoxicity against only the Raji cell line. The results suggest that labdane may serve as a lead structure for developing new anticancer agents targeting certain cancer cell lines and expanded biochemical diversities of labdane-type diterpenoids occurring in nature. However, further studies on the semi-synthesis of their derivatives are required to optimize their pharmacological properties.

## Figures and Tables

**Table 1:** ^1^H (600 MHz) and ^13^C NMR (150 MHz) data for **1**–**3** in CD_3_OD.

No	1		2		3	
	*δ*_H_, Mult (*J* in Hz)	*δ*_C_, Type	*δ*_H_, Mult (*J* in Hz)	*δ*_C_, Type	*δ*_H_, Mult (*J* in Hz)	*δ*_C_, Type
1a	0.81, dd (12.0, 2.0)	49.9, CH_2_	0.90, ol	41.1, CH_2_	0.91, ol	41.0, CH_2_
1b	2.03, m		1.72, ol		1.73, ol	
2a	3.82, m	65.1, CH	1.42, ol	19.4, CH_2_	1.41, ol	19.4, CH_2_
2b			1.64, m		1.63, m	
3a	1.12, m	51.6, CH_2_	1.21, m	43.0, CH_2_	1.19, m	43.1, CH_2_
3b	1.75, ol		1.42, ol		1.40, ol	
4		35.5, C		34.1, C		34.2, C
5	0.88, dd (12.4, 1.8)	53.1, CH	0.90, ol	53.6, CH	0.89, ol	57.0, CH
6a	1.61, dd (12.4, 11.9)	27.4, CH_2_	1.61, m	27.6, CH_2_	1.56, m	18.9, CH_2_
6b	1.75, ol		1.73, ol		1.61, m	
7a	3.81, ol	71.0, CH	3.79, dd (11.6, 5.1)	71.1, CH	1.71, ol	37.9, CH_2_
7b					1.80, m	
8		77.9, C		78.0, C		75.7, C
9	1.50, dd (6.3, 2.5)	53.0, CH	1.46, dd (6.4, 2.5)	53.1, CH	1.33, dd (6.1, 2.6)	56.1, CH
10		41.2, C		39.7, C		40.1, C
11a	2.19, dd (16.7, 4.8)	24.2, CH_2_	2.14, dd (17.6, 4.3)	24.2, CH_2_	2.13, dd (18.0, 4.4)	24.4, CH_2_
11b	2.52, m		2.47, m		2.38, m	
12	5.58, t (6.9)	136.5, CH	5.56, t (6.8)	136.9, CH	5.48, t (6.8)	137.1, CH
13		133.8, C		133.5, C		133.6, C
14	6.33, dd (17.4, 10.8)	142.9, CH	6.33, dd (17.4, 10.8)	142.9, CH	6.33, dd (17.4, 10.7)	142.9, CH
15a	4.87, ol	110.4, CH_2_	4.87, d (10.7)	110.3, CH_2_	4.87, ol	110.4, CH_2_
15b	5.06, d (17.4)		5.05, d (17.4)		5.05, d (17.4)	
16	1.79, s	12.0, CH_3_	1.76, s	12.0, CH_3_	1.76, s	12.0, CH_3_
17a	3.28, d (10.8)	45.6, CH_2_	3.27, d (10.7)	45.7, CH_2_	3.23, d (10.9)	53.4, CH_2_
17b	3.60, d (10.8)		3.59, d (10.7)		3.38, d (10.9)	
18	0.93, s	23.1, CH_3_	0.89, s	22.3, CH_3_	0.87, s	22.2, CH_3_
19	0.99, s	34.1, CH_3_	0.93, s	33.9, CH_3_	0.90, s	34.0, CH_3_
20	1.06, s	16.8, CH_3_	1.01, s	15.8, CH_3_	1.03, s	15.8, CH_3_

‘’ol’’ means overlapped.

**Table 2:** ^1^H (600 MHz) and ^13^C NMR (150 MHz) data for **4** and **5** in CD_3_OD.

No	4		5	
	*δ*_H_, Mult (*J* in Hz)	*δ*_C_, Type	*δ*_H_, Mult (*J* in Hz)	*δ*_C_, Type
1a	1.02, td (13.0, 3.4)	40.3, CH_2_	0.90, ol	49.6, CH_2_
1b	1.84, ol		2.09, ol	
2a	1.46, m	19.7, CH_2_	3.85, m	65.2, CH
2b	1.64, m			
3a	1.23, td (13.3, 3.8)	43.1, CH_2_	1.16, d (13.2)	51.6, CH_2_
3b	1.43, m		1.77, m	
4		34.4, C		35.7, C
5	1.10, dd (12.4, 2.6)	56.2, CH	1.13, dd (12.2, 2.0)	53.3, CH
6a	1.61, m	21.2, CH_2_	1.53, m, CH_2_	30.5, CH_2_
6b	1.71, m		1.97, m	
7a	1.31, m	37.1, CH_2_	3.72, dd (11.7, 5.0)	70.3, CH
7b	1.97, td (13.7, 5.0)			
8		59.3, C		60.9, C
9	1.58, m	54.4, CH	1.58, m	53.2, CH
10		40.9, C		42.2, C
11a	1.73, m	21.6, CH_2_	1.86, m	21.7, CH_2_
11b	2.04, dd (16.7, 5.4)		2.12, m	
12	5.34, t (6.8)	136.2, CH	5.36, t (6.8)	135.5, CH
13		134.7, C		135.1, C
14	6.32, dd (17.4, 10.8)	142.5, CH	6.34, dd (17.4, 10.7)	142.4, CH
15a	4.88, ol	110.7, CH_2_	4.89, ol	110.9, CH_2_
15b	5.05, d (17.4)		5.08, d (17.4)	
16	1.70, s	12.1, CH_3_	1.73, s	12.2, CH_3_
17a	2.26, d (4.3)	50.0, CH_2_	2.41, d (4.8)	45.0, CH_2_
17b	2.56, d (4.3)		2.81, d (4.8)	
18	0.89, s	22.2, CH_3_	0.94, s	23.0, CH_3_
19	0.92, s	34.0, CH_3_	1.00, s	34.0, CH_3_
20	0.93, s	15.2, CH_3_	0.96, s	16.3, CH_3_

**Table 3:** Antibacterial activity of **1**–**6**.

Strains	MIC (µg/mL)						
	1	2	3	4	5	6	Kanamycin
*Bacillus subtilis*	>128	4	32	>128	>128	32	0.5
*Micrococcus luteus*	>128	8	>128	>128	>128	>128	0.5
*Staphylococcus aureus*	>128	8	>128	>128	>128	>128	1

Kanamycin as a positive control. MIC values are minimum inhibitory concentrations.

**Table 4:** Growth inhibition (IC_50_, µM) of **1**, **2,** and **4** against blood cancer cell lines.

Cell Lines	IC_50_ (µM)			
	1	2	4	Doxorubicin
HL-60	>30	>30	>30	0.018
Raji	15.520	3.406	17.110	0.011
WSU-DLCL2	>30	1.182	14.170	0.006
NALM6 C. G5	>30	22.460	>30	0.003

Doxorubicin as a positive control. IC_50_ values are the concentration corresponding to 50% cell growth inhibition. **3** and **5** were not tested due to the limited amount of samples.

## Data Availability

The data presented in the article are available in the Supplementary Materials.

## Supplementary Materials

The following supporting information can be downloaded at: https://www.mdpi.com/article/10.3390/ijms25063311/s1.
