# Supplementary material for: Labdane-Type Diterpenoids from Streptomyces griseorubens and Their Antimicrobial and Cytotoxic Activities
Source: Int J Mol Sci. 2024 Mar 14;25(6):3311. doi: 10.3390/ijms25063311 (PMC10970165; doi:10.3390/ijms25063311)
Supplement: Supplementary file 1 [file ijms-25-03311-s001.zip › ijms-2886759-supplementary.pdf]

Labdane-Type Diterpenoids from *Streptomyces griseorubens* and Their Antimicrobial and Cytotoxic Activities

Chang-Su Heo [1,2], Jong Soon Kang [3], Jeong-Wook Yang [3], Min Ah Lee[1,4], Hwa-Sun Lee[1,4] and Hee Jae Shin [1,2,*]

Contents

Figure S1. Structures of **1-6** isolated from *Streptomyces griseorubens* 2210JJ-087. 4

Figure S2. HRESIMS data of **1**. 5

Figure S3. ^{1}H NMR spectrum of **1** (CD_3OD). 6

Figure S4. ^{13}C NMR spectrum of **1** (CD_3OD). 7

Figure S5. HSQC spectrum of **1** (CD_3OD). 8

Figure S6. ^{1}H-^{1}H COSY spectrum of **1** (CD_3OD). 9

Figure S7. HMBC spectrum of **1** (CD_3OD). 10

Figure S8. NOESY spectrum of **1** (CD_3OD). 11

Figure S9. HSQC spectrum of (*S*)-di-MTPA esters of **1** (**1a**) (CD_3OD). 12

Figure S10. ^{1}H-^{1}H COSY spectrum of (*S*)-di-MTPA esters of **1** (**1a**) (CD_3OD). 13

Figure S11. ^{1}H NMR spectrum of (*S*)-di-MTPA esters of **1** (**1a**) (CD_3OD). 14

Figure S12. HSQC spectrum of (*R*)-di-MTPA esters of **1** (**1b**) (CD_3OD). 15

Figure S13. ^{1}H-^{1}H COSY spectrum of (*R*)-di-MTPA esters of **1** (**1b**) (CD_3OD). 16

Figure S14. ^{1}H NMR spectrum of (*R*)-di-MTPA esters of **1** (**1b**) (CD_3OD). 17

Figure S15. Summarized $\Delta\delta$ values (in ppm) = δ_S - δ_R obtained for (*S*)- and (*R*)-di-MPTA esters of **1**. 18

Figure S16. HRESIMS data of **2**. 19

Figure S17. ^{1}H NMR spectrum of **2** (CD_3OD). 20

Figure S18. ^{13}C NMR spectrum of **2** (CD_3OD). 21

Figure S19. HSQC NMR spectrum of **2** (CD_3OD). 22

Figure S20. ^{1}H-^{1}H COSY NMR spectrum of **2** (CD_3OD). 23

Figure S21. HMBC NMR spectrum of **2** (CD_3OD).. 24

Figure S22. NOESY NMR spectrum of **2** (CD_3OD). 25

Figure S23. HSQC spectrum of (*S*)-MTPA esters of **2** (**2a**) (CD_3OD). 26

Figure S24. ^{1}H-^{1}H COSY spectrum of (*S*)-MTPA esters of **2** (**2a**) (CD_3OD). 27

Figure S25. ^{1}H NMR spectrum of (*S*)-MTPA esters of **2** (**2a**) (CD_3OD). 28

Figure S26. HSQC spectrum of (*R*)-MTPA esters of **2** (**2b**) (CD_3OD). 29

Figure S27. ^{1}H-^{1}H COSY spectrum of (*R*)-MTPA esters of **2** (**2b**) (CD_3OD). 30

Figure S28. ^{1}H NMR spectrum of (*R*)-MTPA esters of **2** (**2b**) (CD_3OD). 31

Figure S29. Summarized Δδ values (in ppm) = δ_S - δ_R obtained for (*S*)- and (*R*)-MPTA esters of **2**. 32
Figure S30. HRESIMS data of **3**. 33
Figure S31. ^{1}H NMR spectrum of **3** (CD_3OD). 34
Figure S32. ^{13}C NMR spectrum of **3** (CD_3OD). 35
Figure S33. HSQC NMR spectrum of **3** (CD_3OD). 36
Figure S34. ^{1}H-^{1}H COSY NMR spectrum of **3** (CD_3OD). 37
Figure S35. HMBC NMR spectrum of **3** (CD_3OD). 38
Figure S36. 1D selective NOESY data of **3** (irradiated H-9) (CD_3OD). 39
Figure S37. 1D selective NOESY data of **3** (irradiated H-14) (CD_3OD). 40
Figure S38. HRESIMS data of **4**. 40
Figure S39. ^{1}H NMR spectrum of **4** (CD_3OD). 42
Figure S40. ^{13}C NMR spectrum of **4** (CD_3OD). 42
Figure S41. HSQC NMR spectrum of **4** (CD_3OD). 43
Figure S42. ^{1}H-^{1}H COSY NMR spectrum of **4** (CD_3OD). 44
Figure S43. HMBC NMR spectrum of **4** (CD_3OD). 45
Figure S44. NOESY NMR spectrum of **4** (CD_3OD). 47
Figure S45. HRESIMS data of **5**. 47
Figure S46. ^{1}H NMR spectrum of **5** (CD_3OD). 49
Figure S47. ^{13}C NMR spectrum of **5** (CD_3OD). 49
Figure S48. HSQC NMR spectrum of **5** (CD_3OD). 50
Figure S49. ^{1}H-^{1}H COSY NMR spectrum of **5** (CD_3OD). 51
Figure S50. HMBC NMR spectrum of **5** (CD_3OD). 52
Figure S51. NOESY NMR spectrum of **5** (CD_3OD). 53
Figure S52. LRESIMS data of **6**. 54
Figure S53. ^{1}H NMR spectrum of **6** (CD_3OD). 55
Figure S54. ^{13}C NMR spectrum of **6** (CD_3OD). 56
Table S1. Results of the cytotoxicity test of **1**, **2**, and **4**. 58

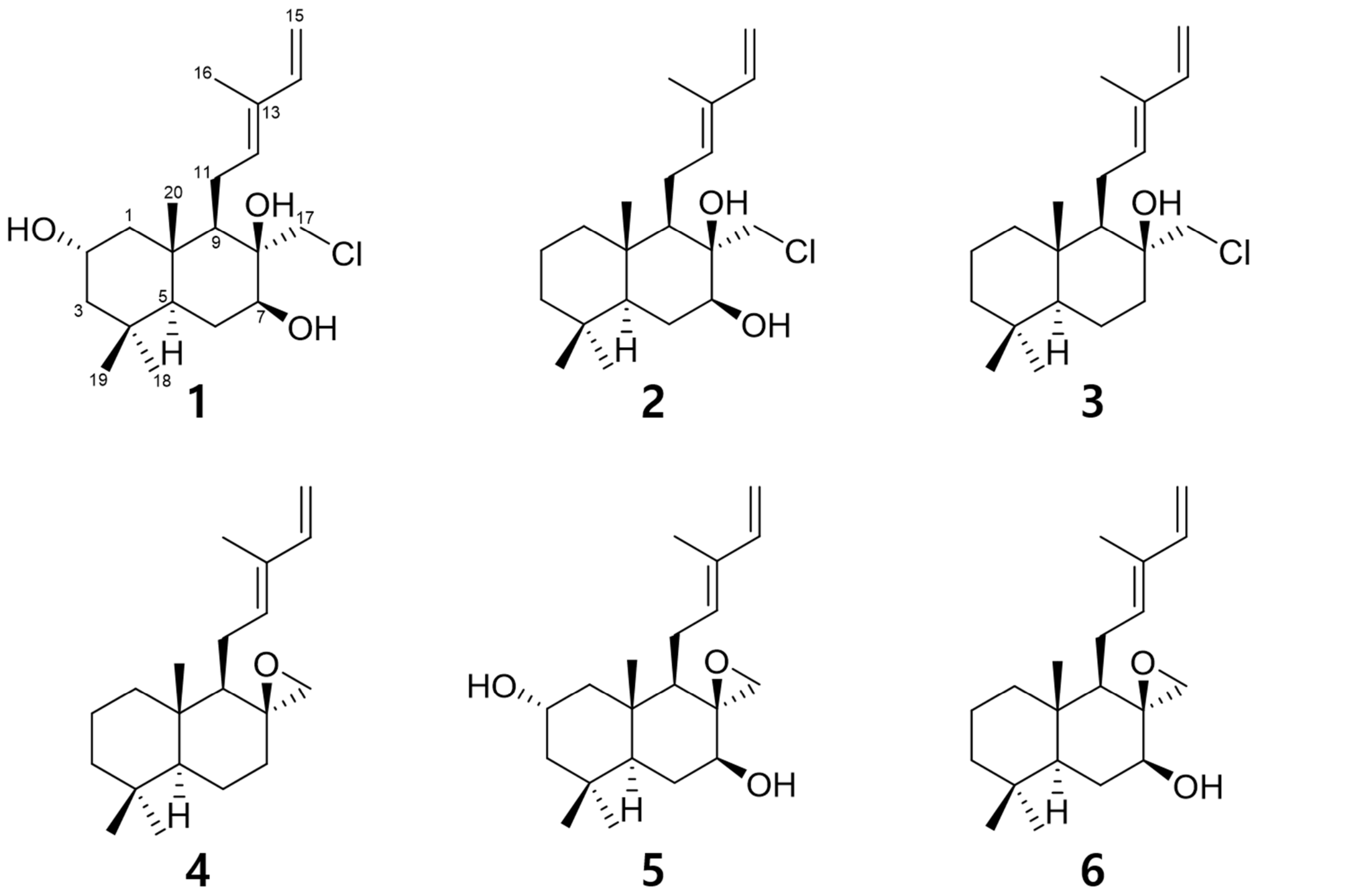

Figure S1. Structures of **1**-**6** isolated from *Streptomyces griseorubens* 2210JJ-087.

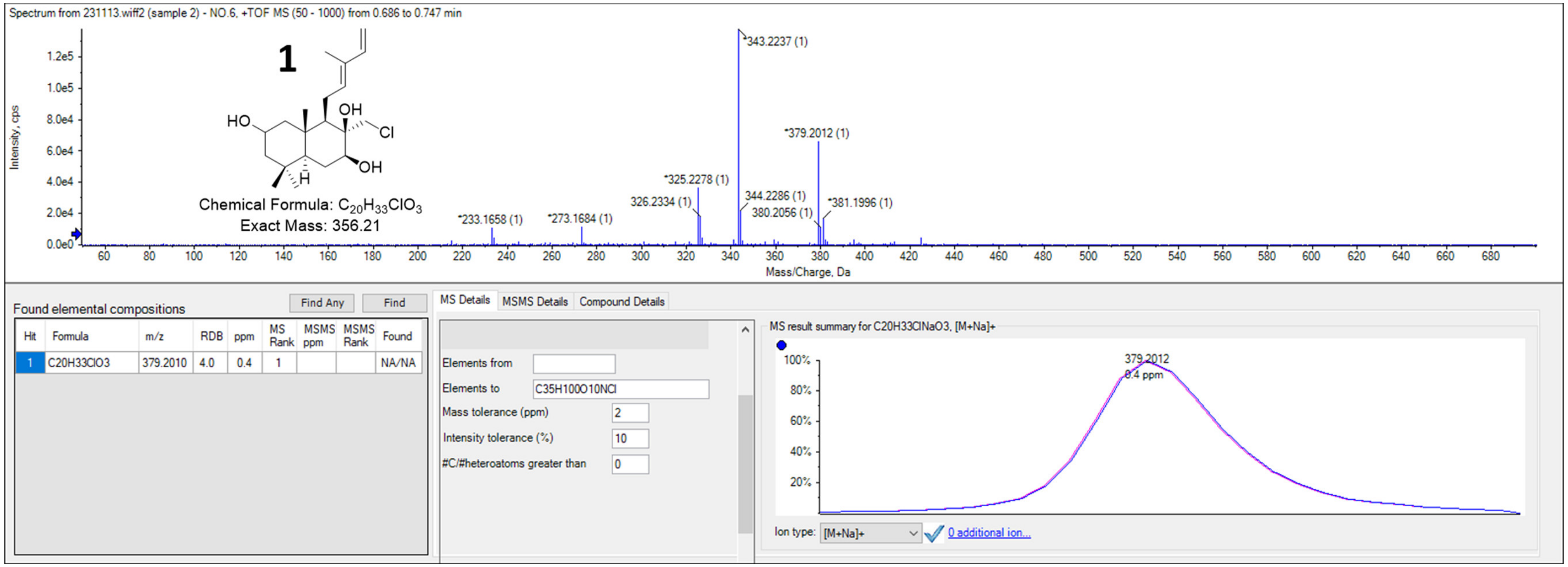

Figure S2. HRESIMS data of **1**.

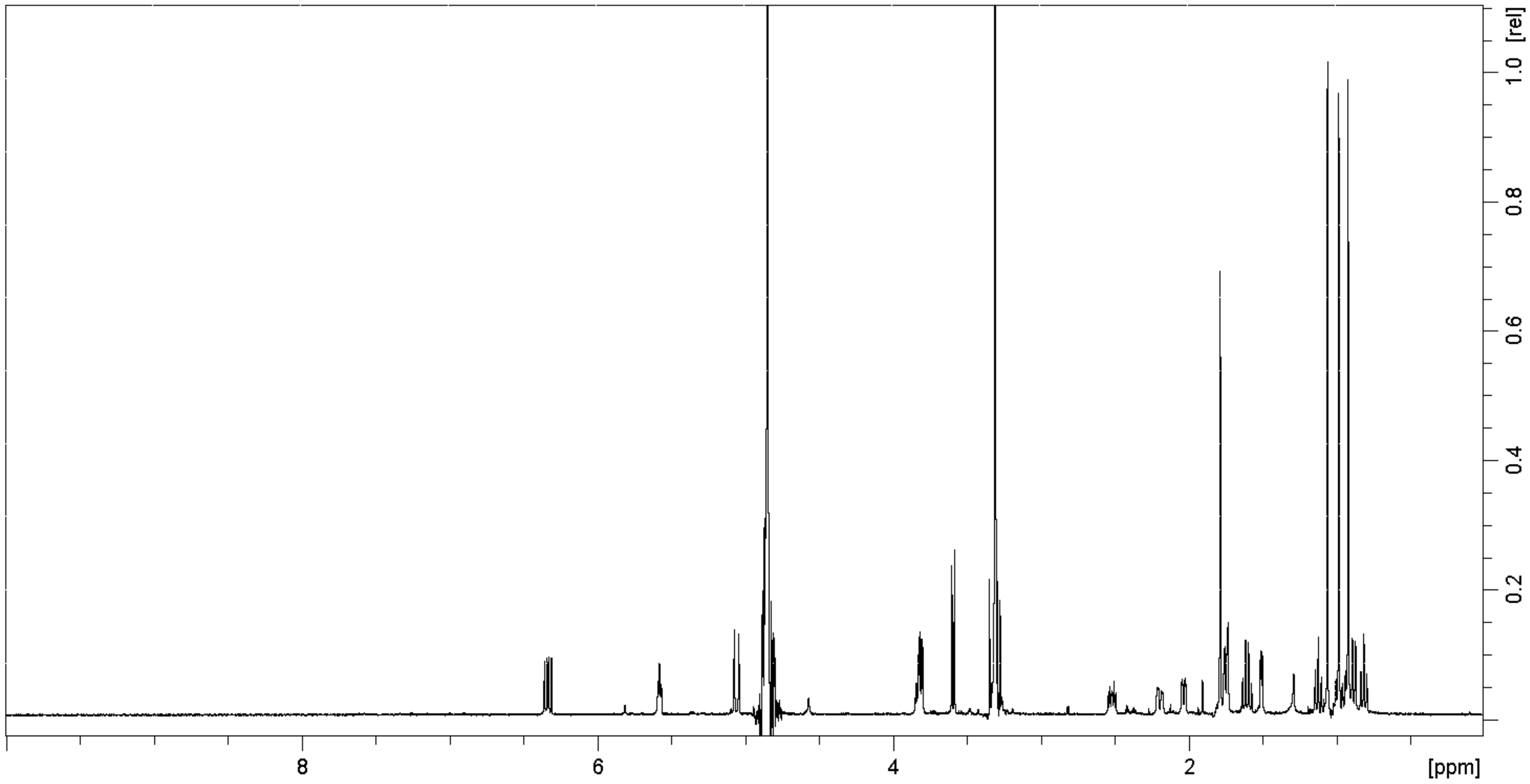

Figure S3. ^{1}H NMR spectrum of **1** (CD_3OD).

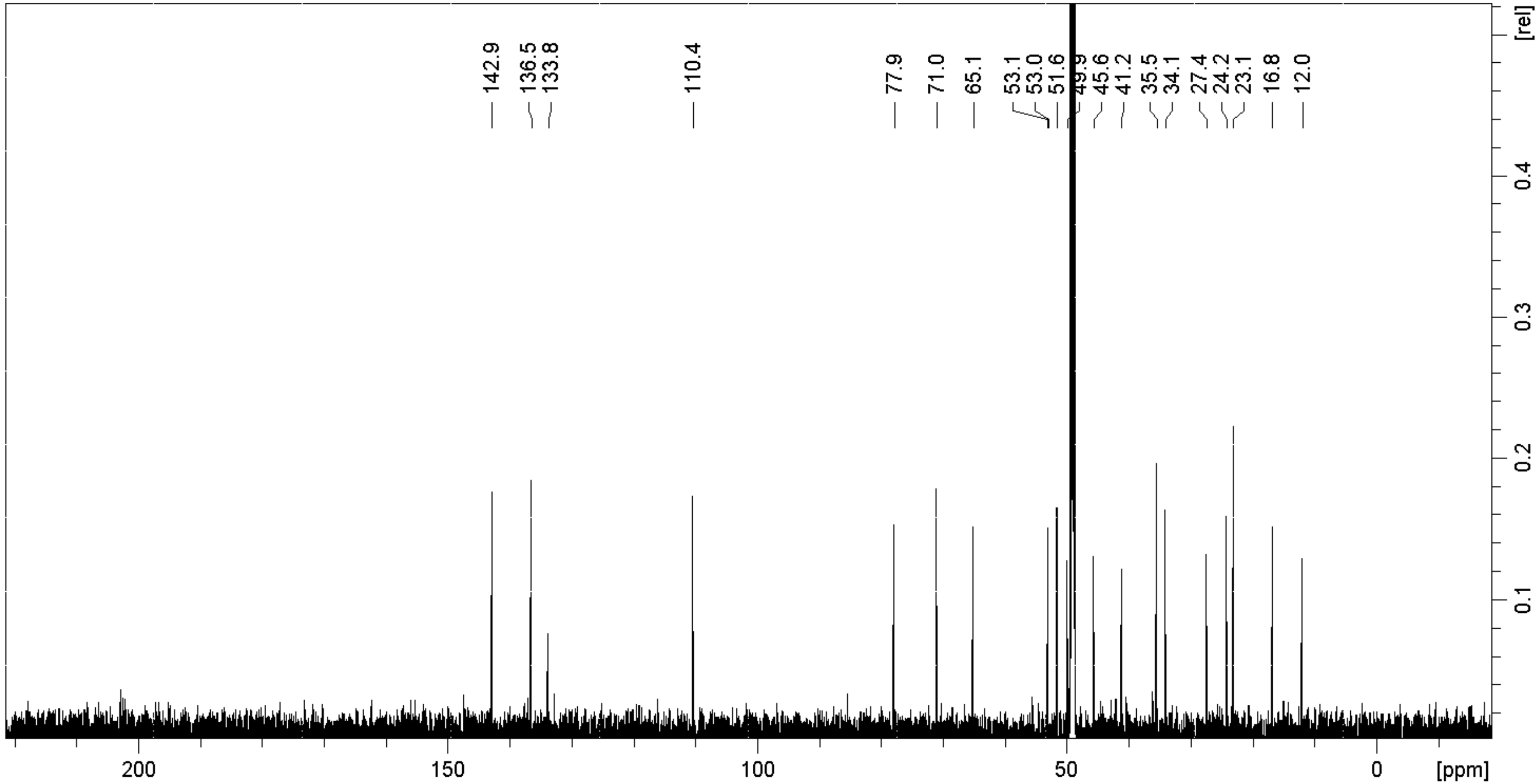

Figure S4. ^{13}C NMR spectrum of **1** (CD_3OD).

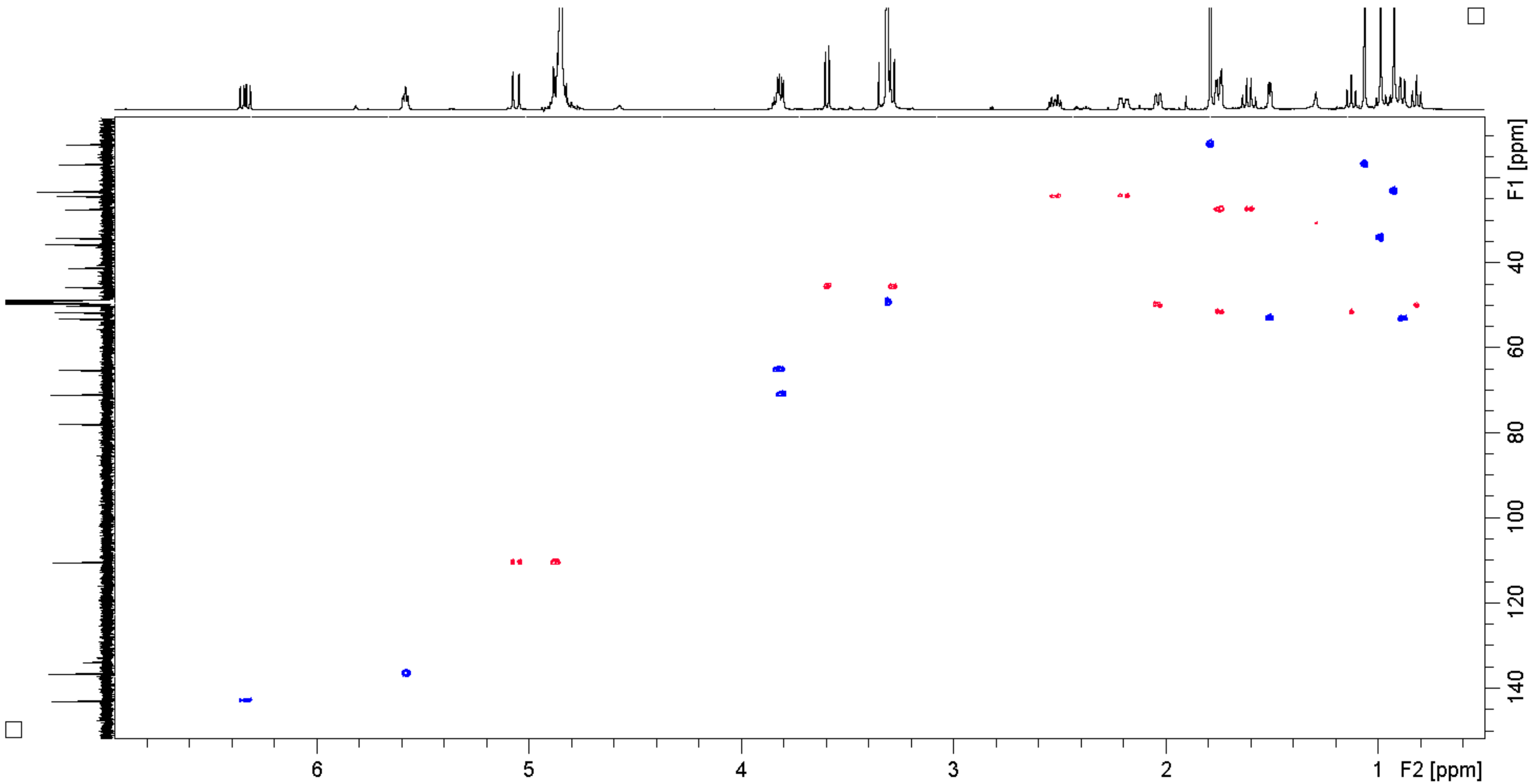

Figure S5. HSQC spectrum of **1** (CD_3OD).

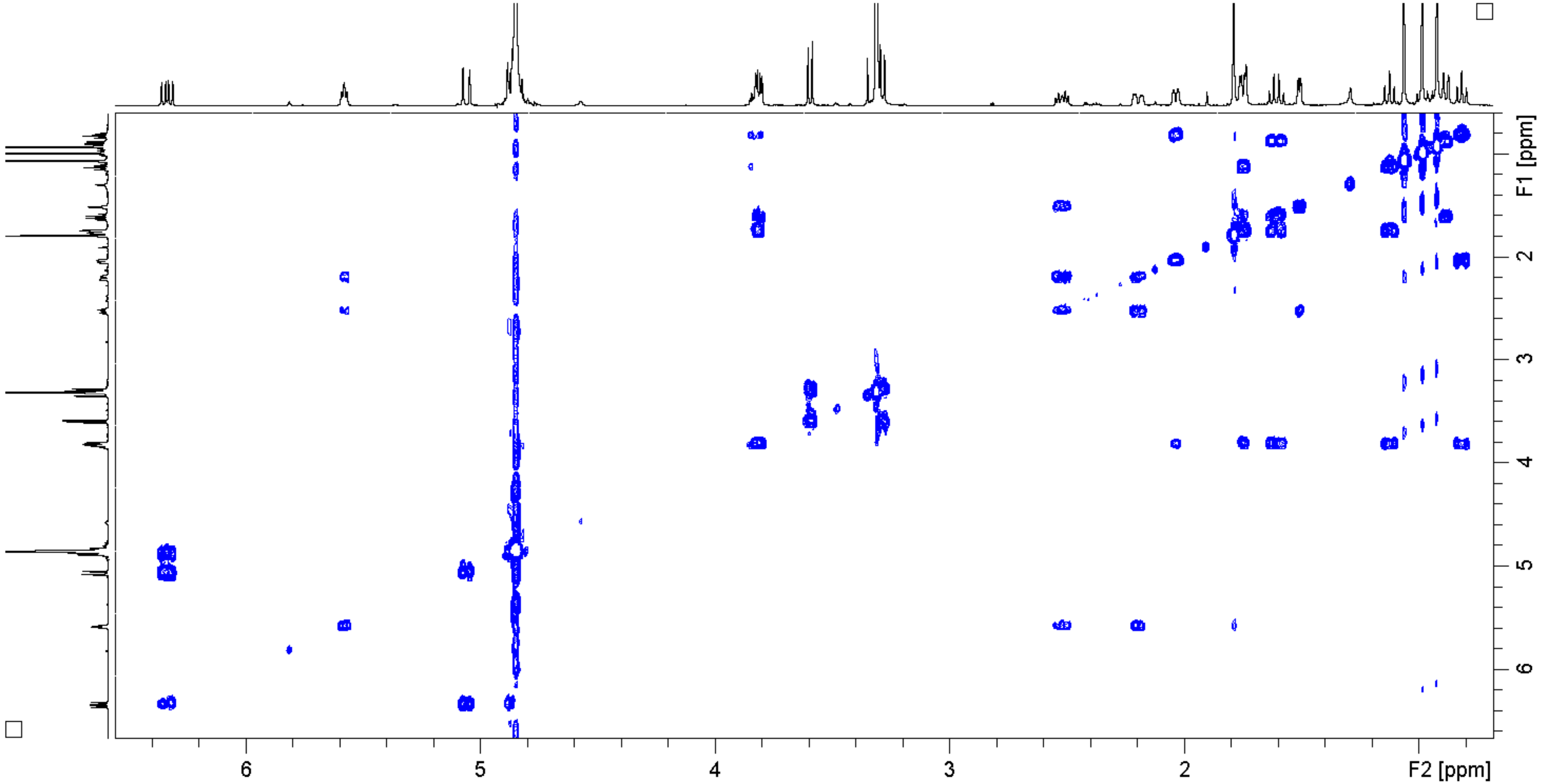

Figure S6. ^{1}H-^{1}H COSY spectrum of **1** (CD_3OD).

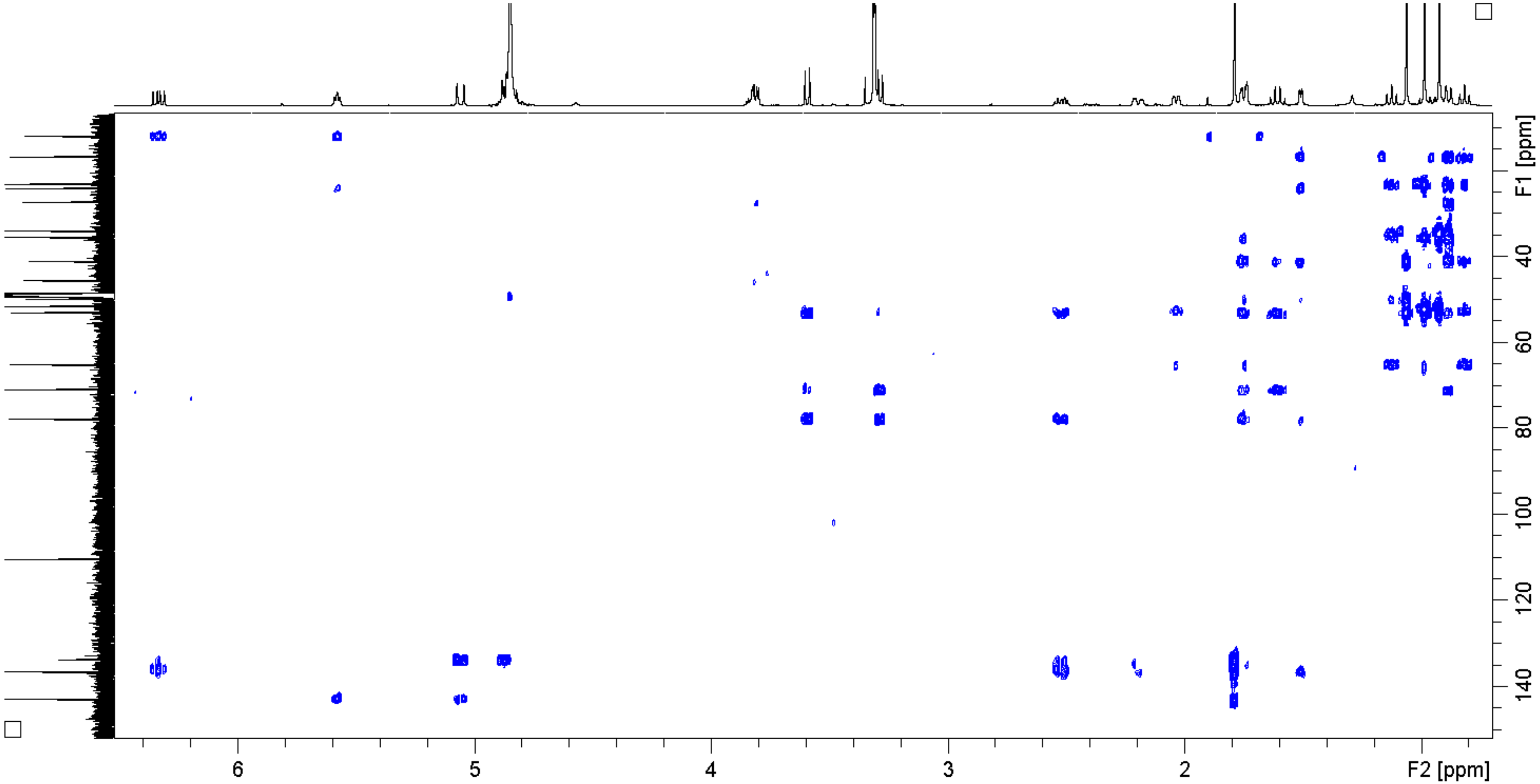

Figure S7. HMBC spectrum of **1** (CD_3OD).

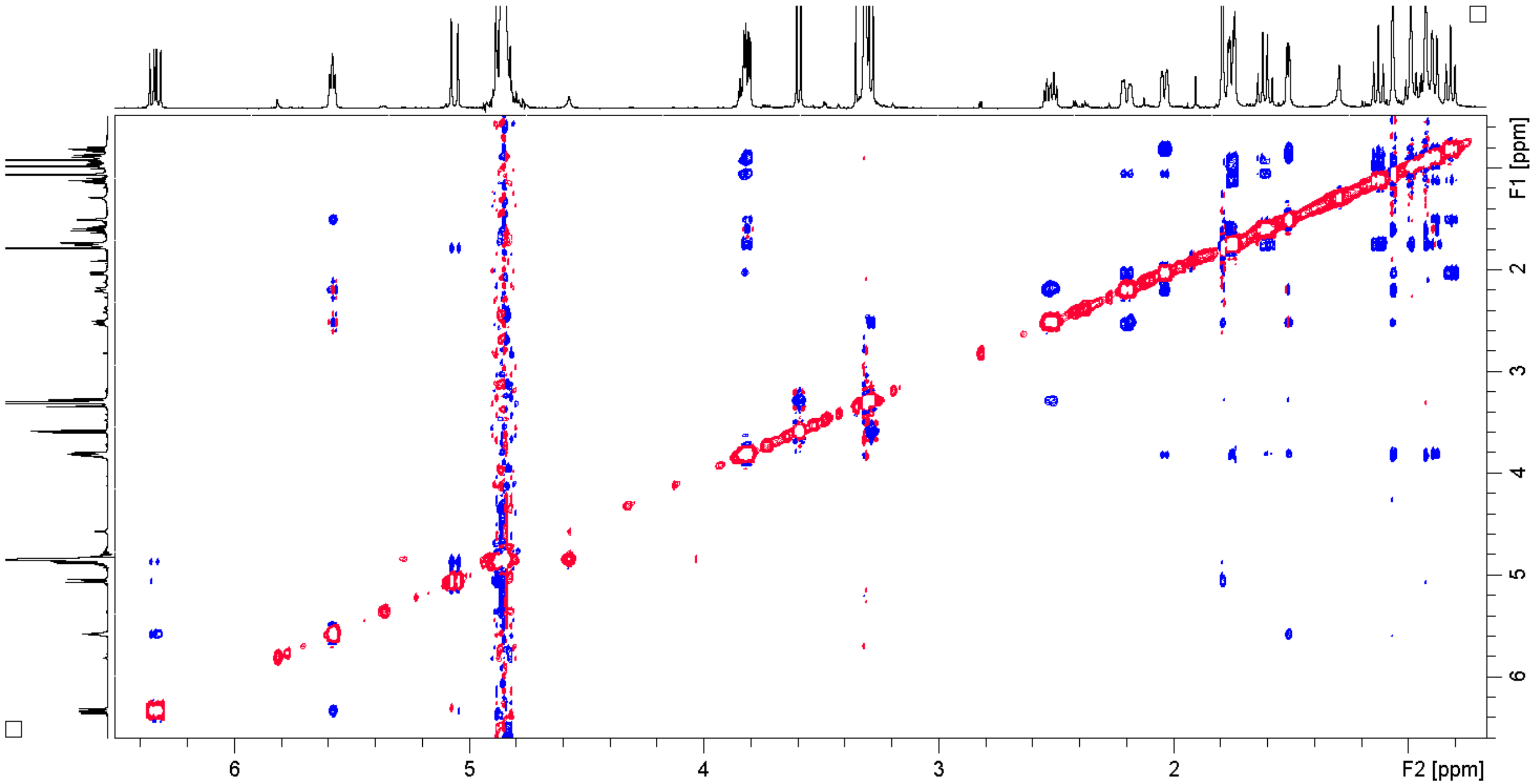

Figure S8. NOESY spectrum of **1** (CD_3OD).

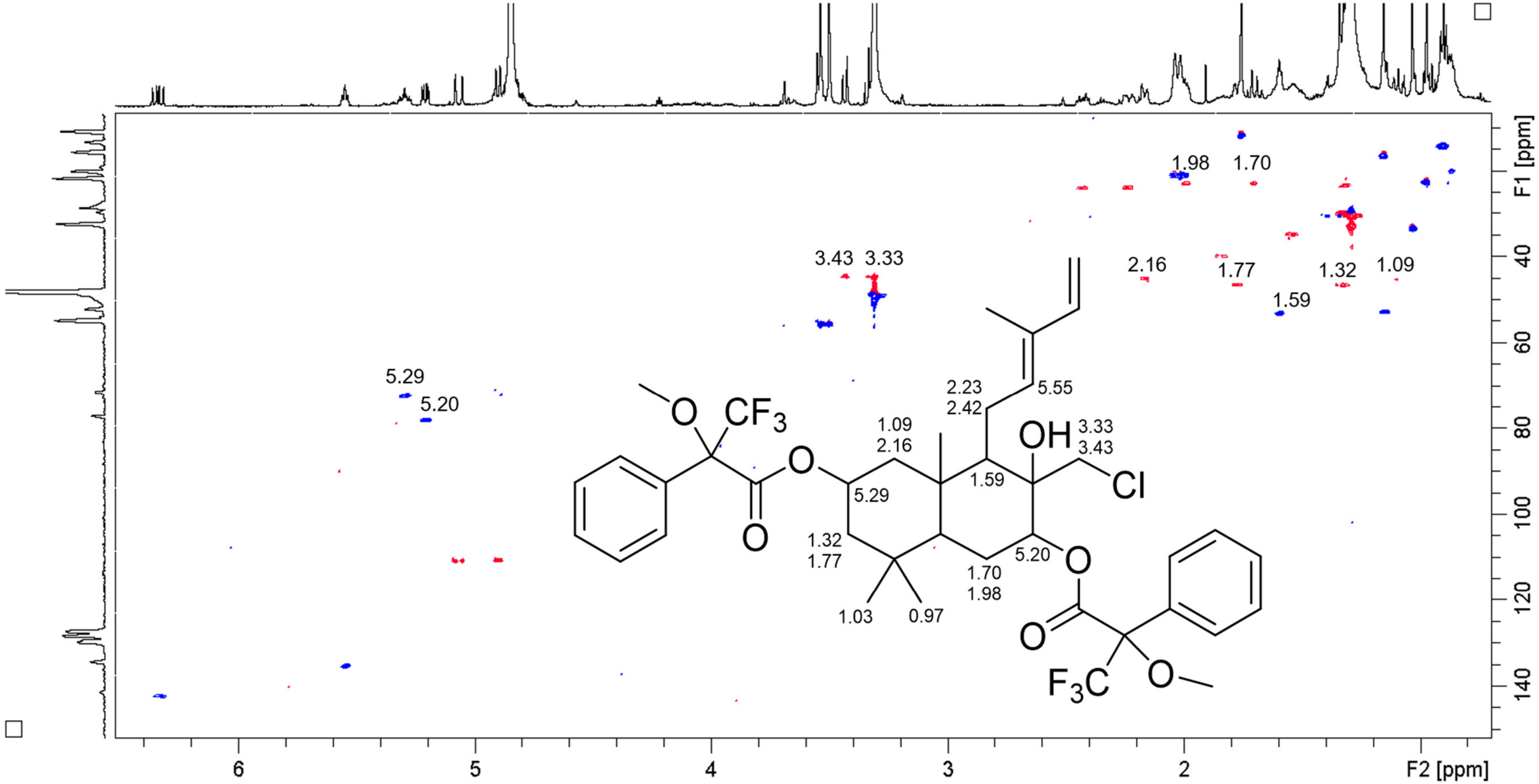

Figure S9. HSQC spectrum of (*S*)-di-MTPA esters of **1** (**1a**) (CD_3OD).

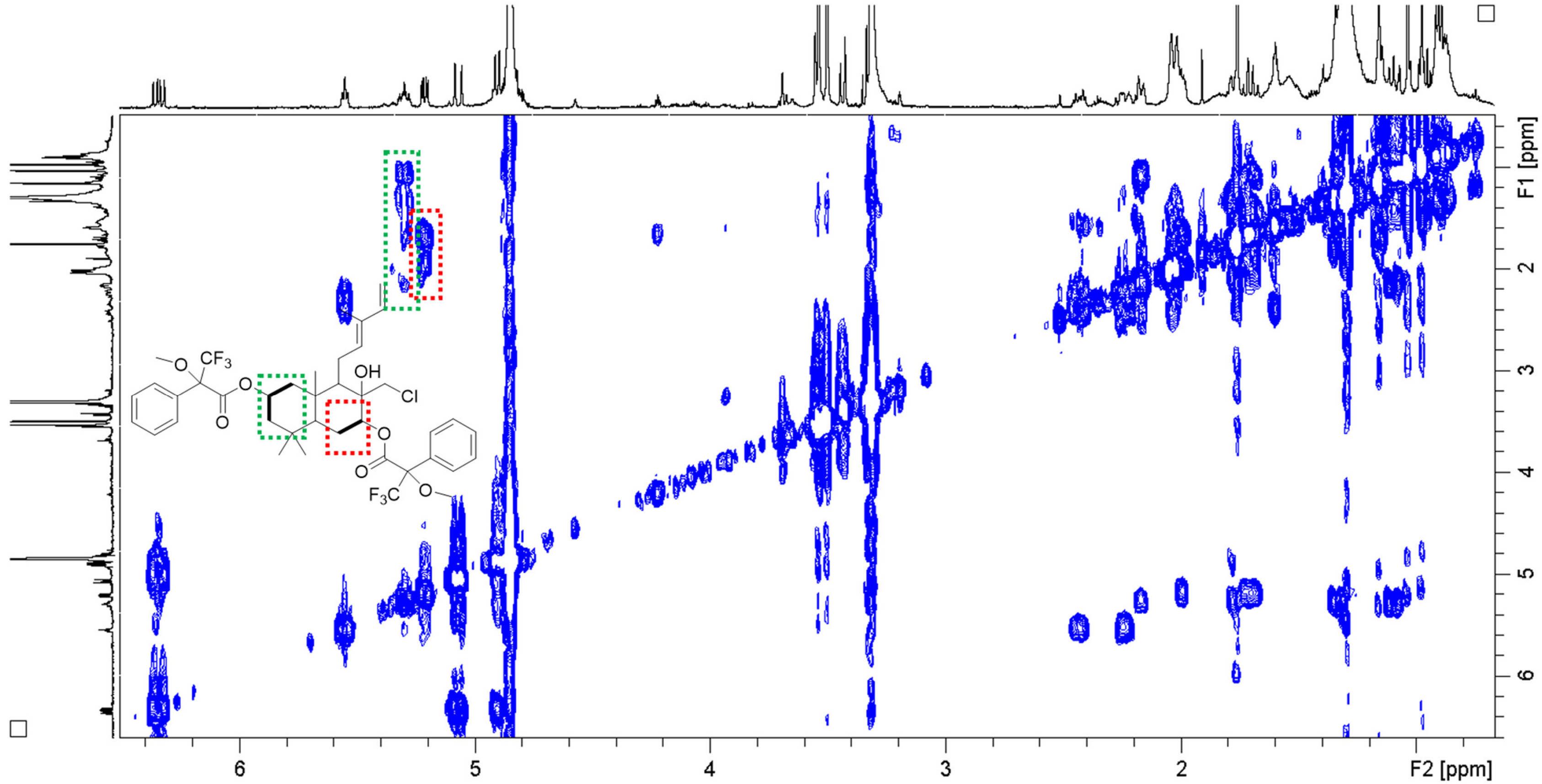

Figure S10. ^{1}H-^{1}H COSY spectrum of (*S*)-di-MTPA esters of **1** (**1a**) (CD_3OD).

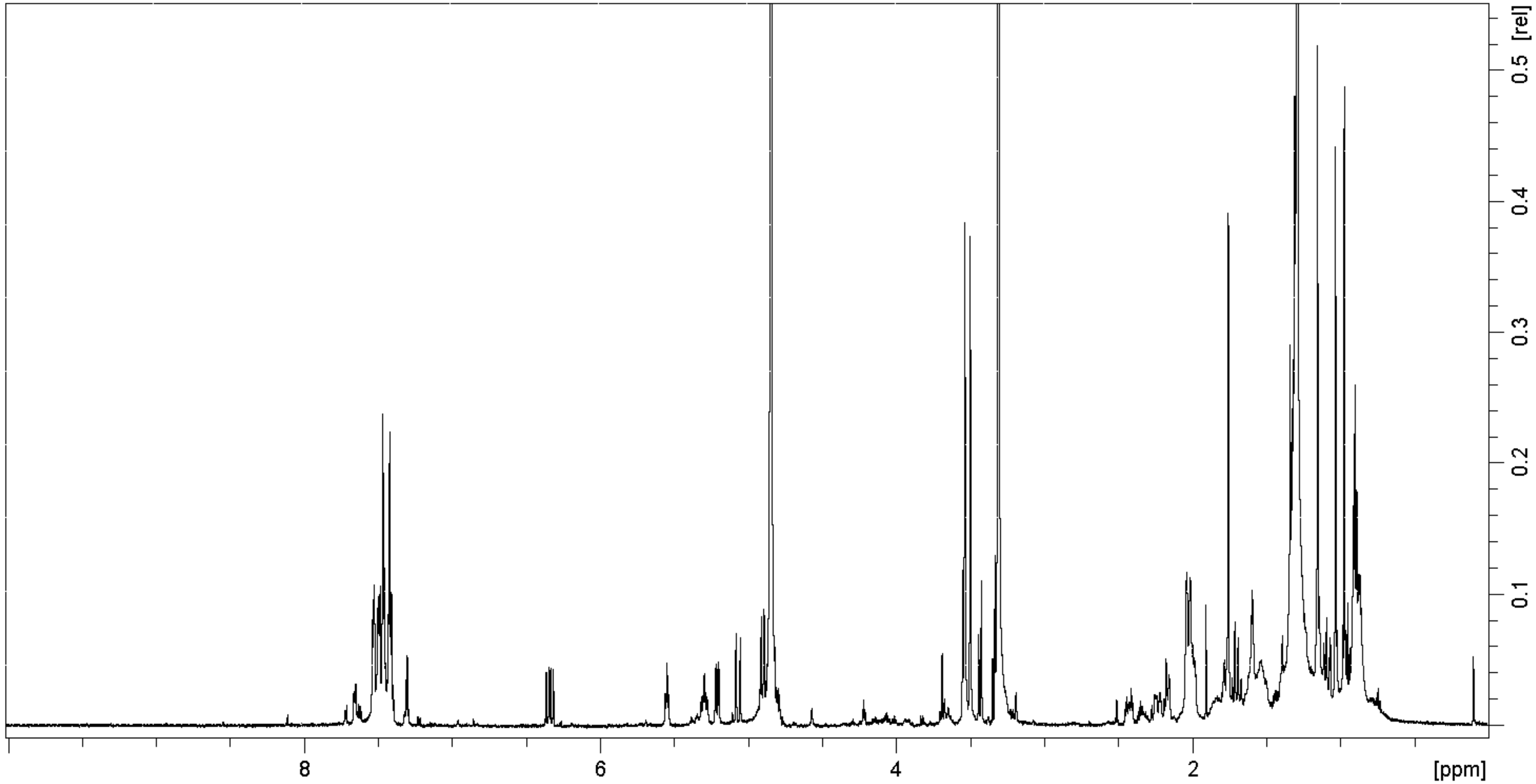

Figure S11. ^{1}H NMR spectrum of (*S*)-di-MTPA esters of **1** (**1a**) (CD_3OD).

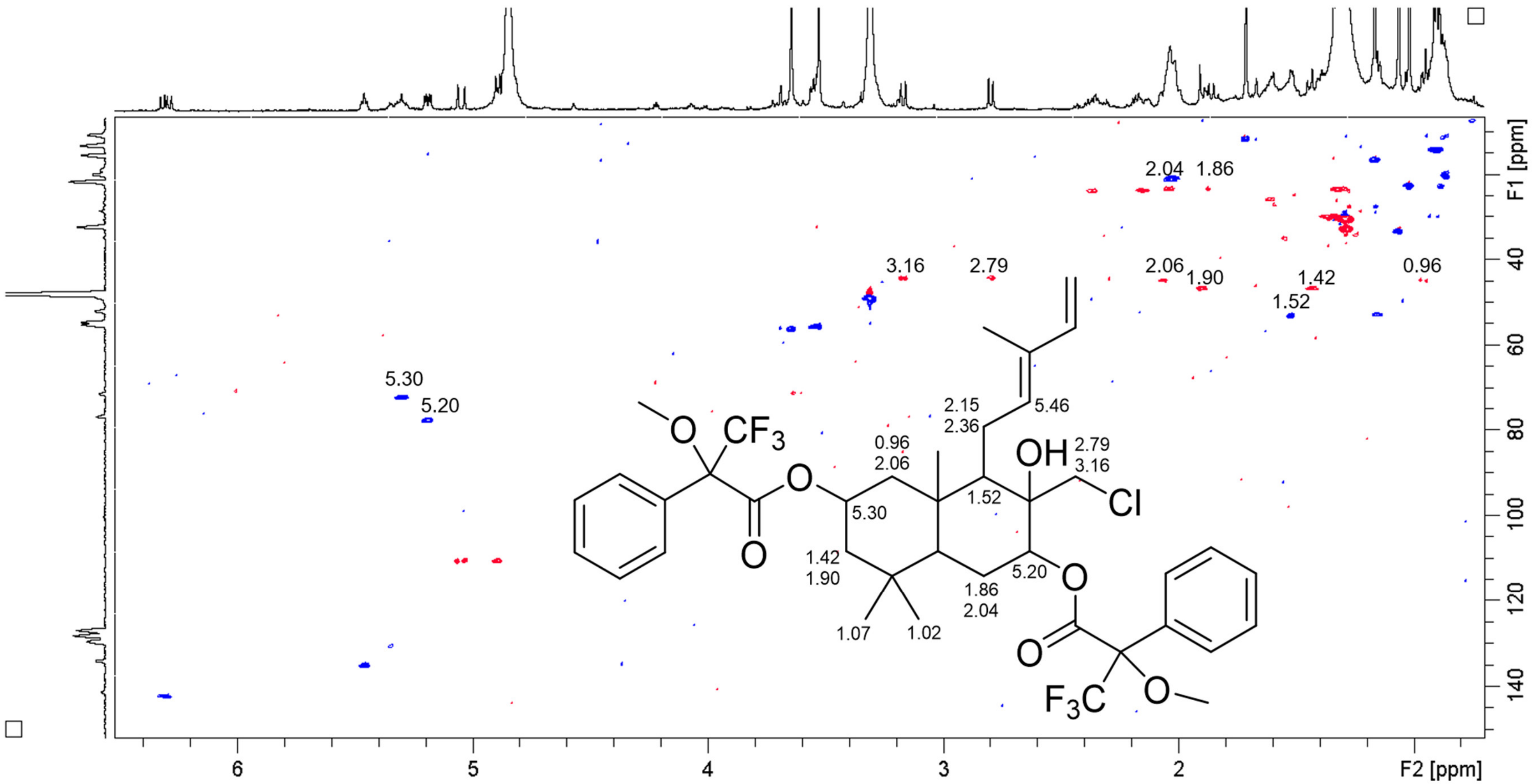

Figure S12. HSQC spectrum of (*R*)-di-MTPA esters of **1** (**1b**) (CD_3OD).

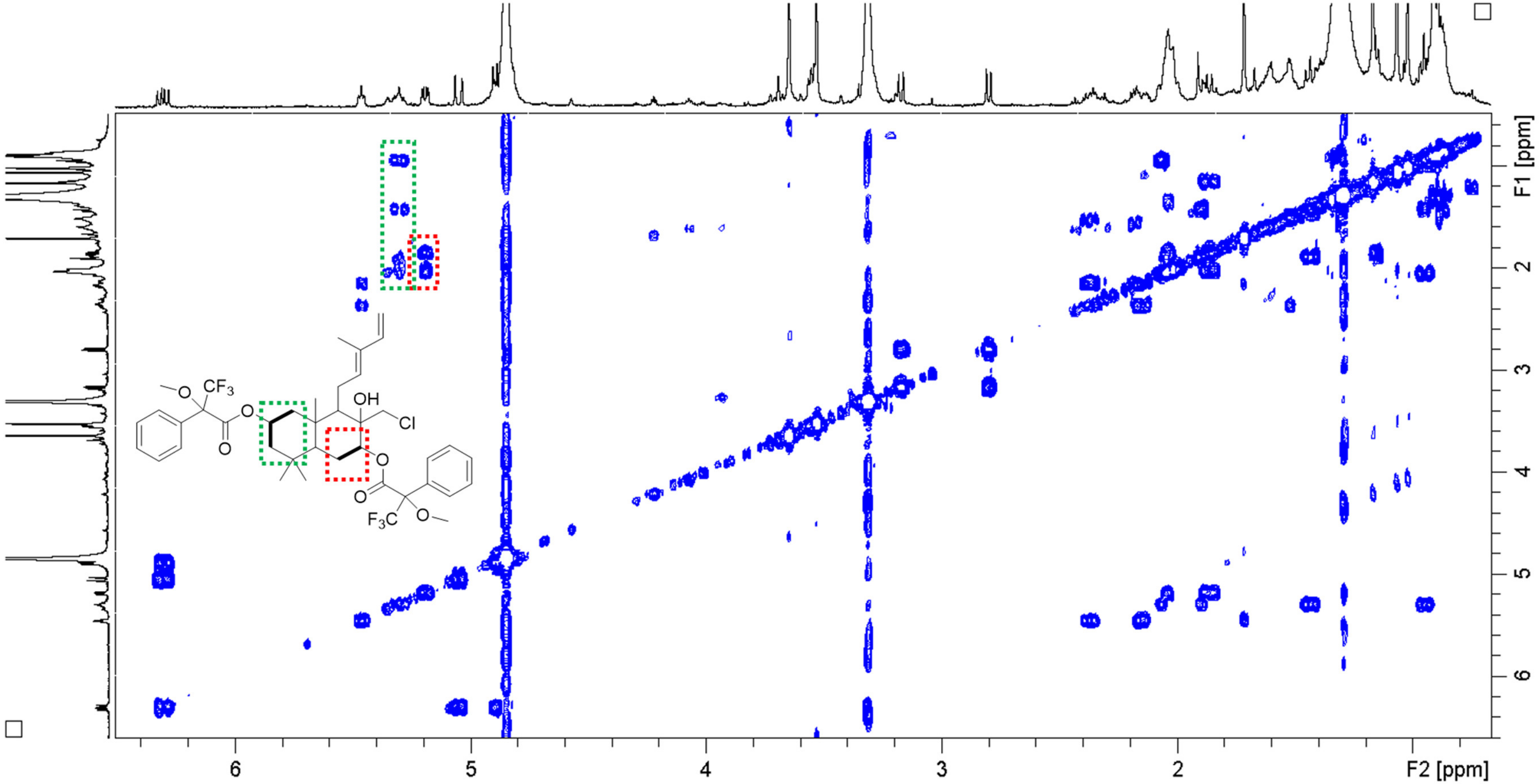

Figure S13. ^{1}H-^{1}H COSY spectrum of (*R*)-di-MTPA esters of **1** (**1b**) (CD_3OD).

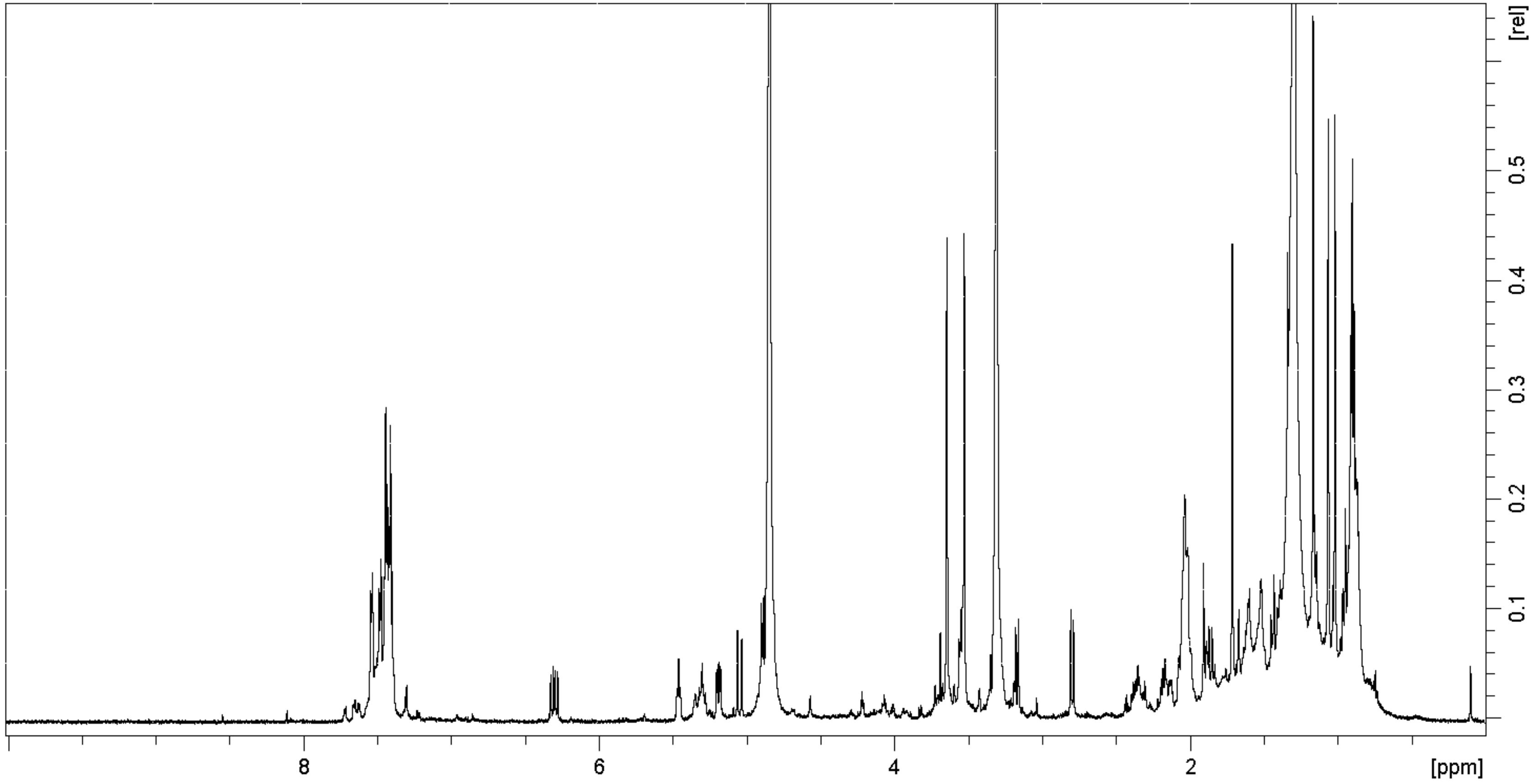

Figure S14. ^{1}H NMR spectrum of (*R*)-di-MTPA esters of **1** (**1b**) (CD_3OD).

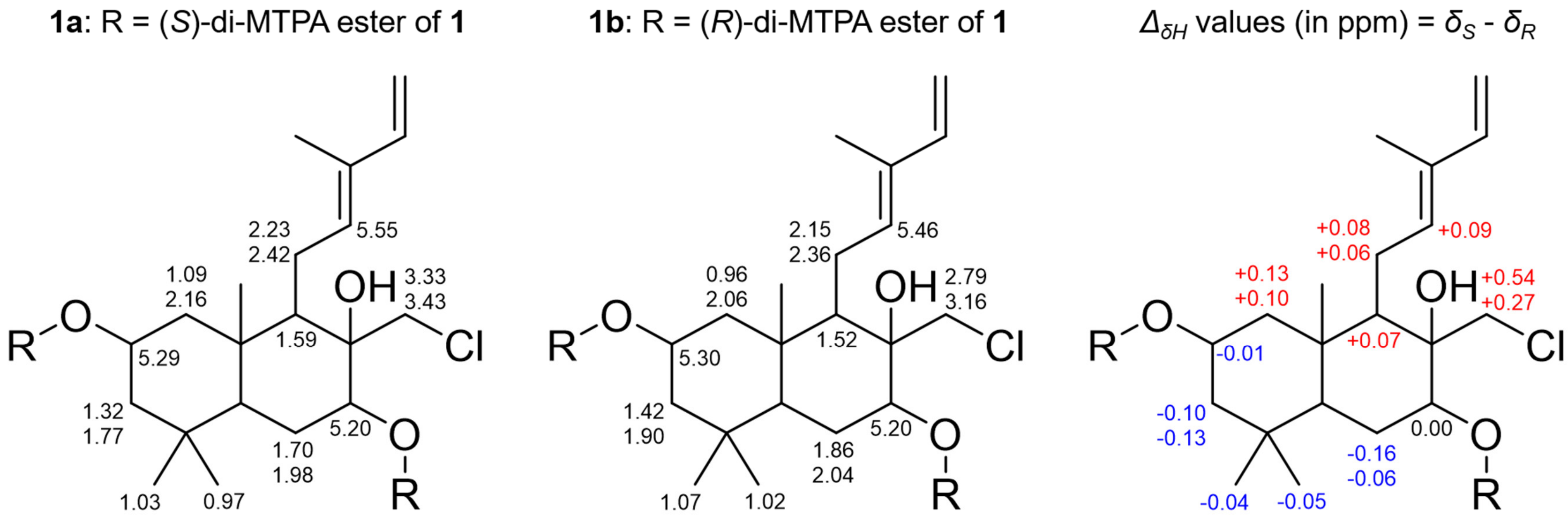

Figure S15. Summarized Δδ values (in ppm) = δ*S* - δ*R* obtained for (*S*)- and (*R*)-di-MPTA esters of **1**.

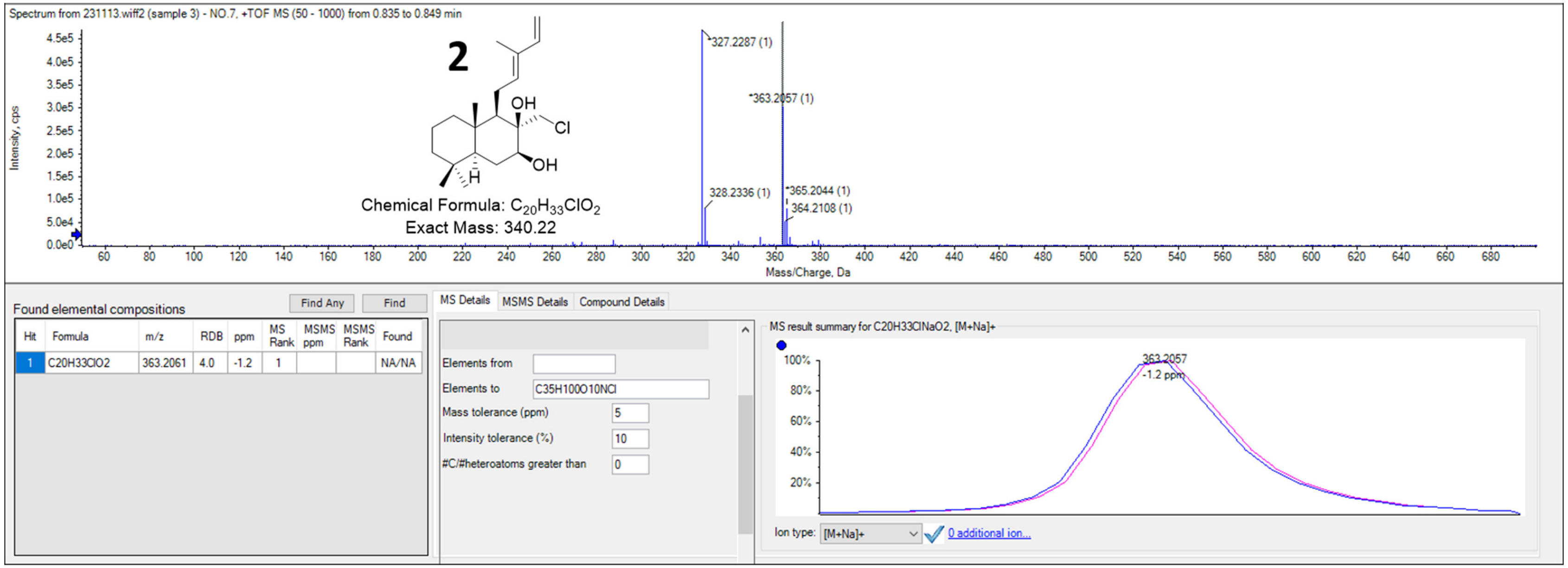

Figure S16. HRESIMS data of **2**.

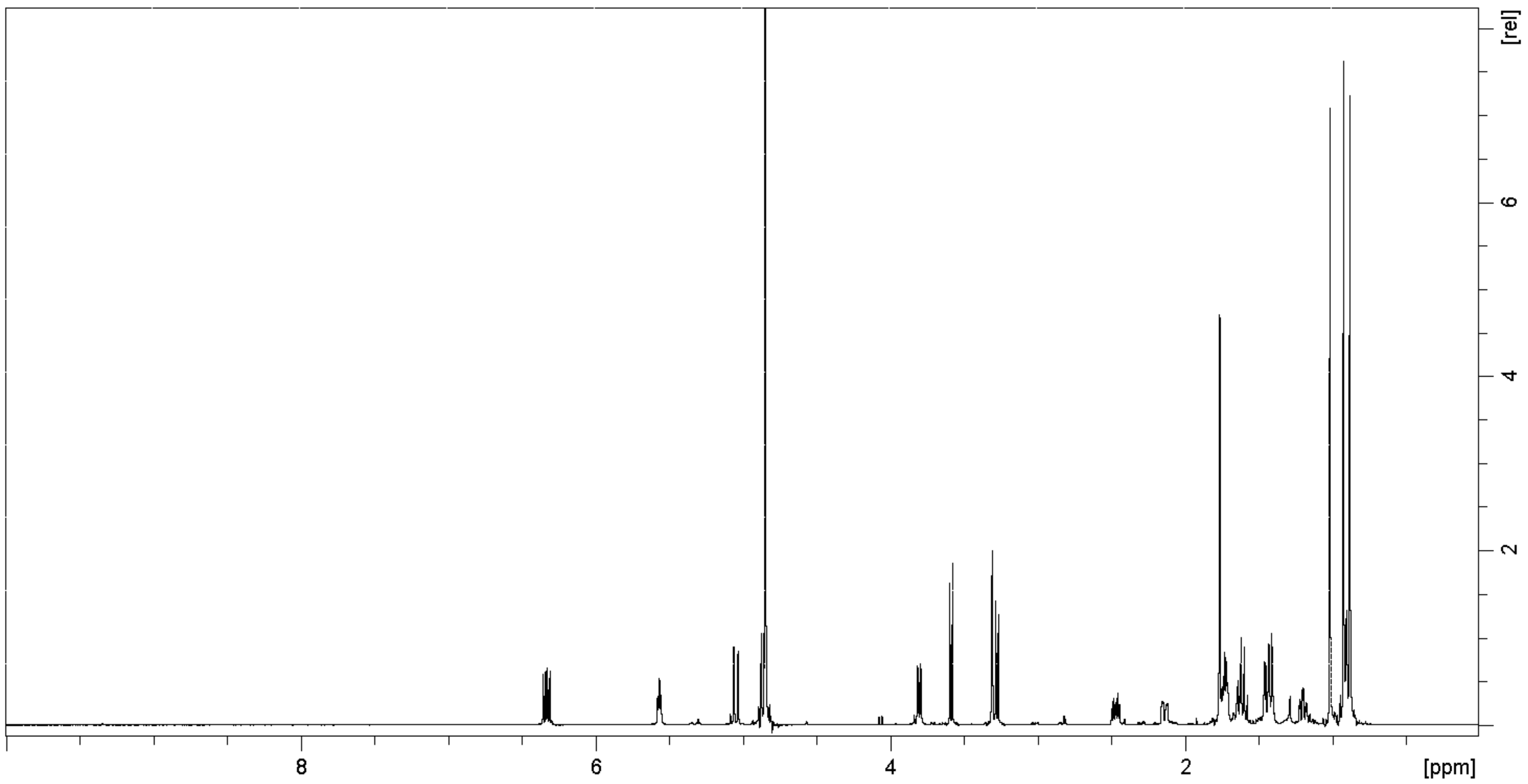

Figure S17. ^{1}H NMR spectrum of **2** (CD_3OD).

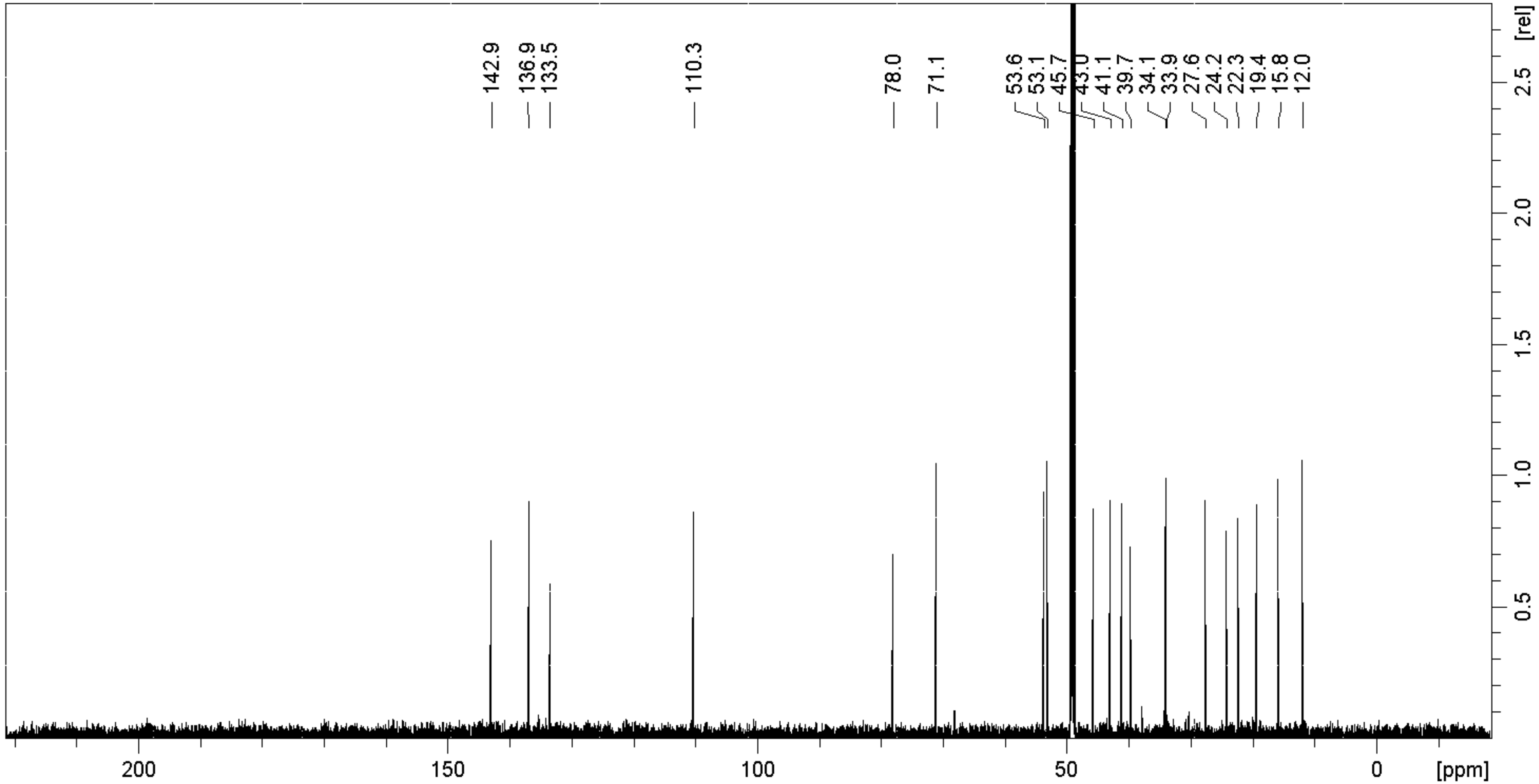

Figure S18. ^{13}C NMR spectrum of **2** (CD_3OD).

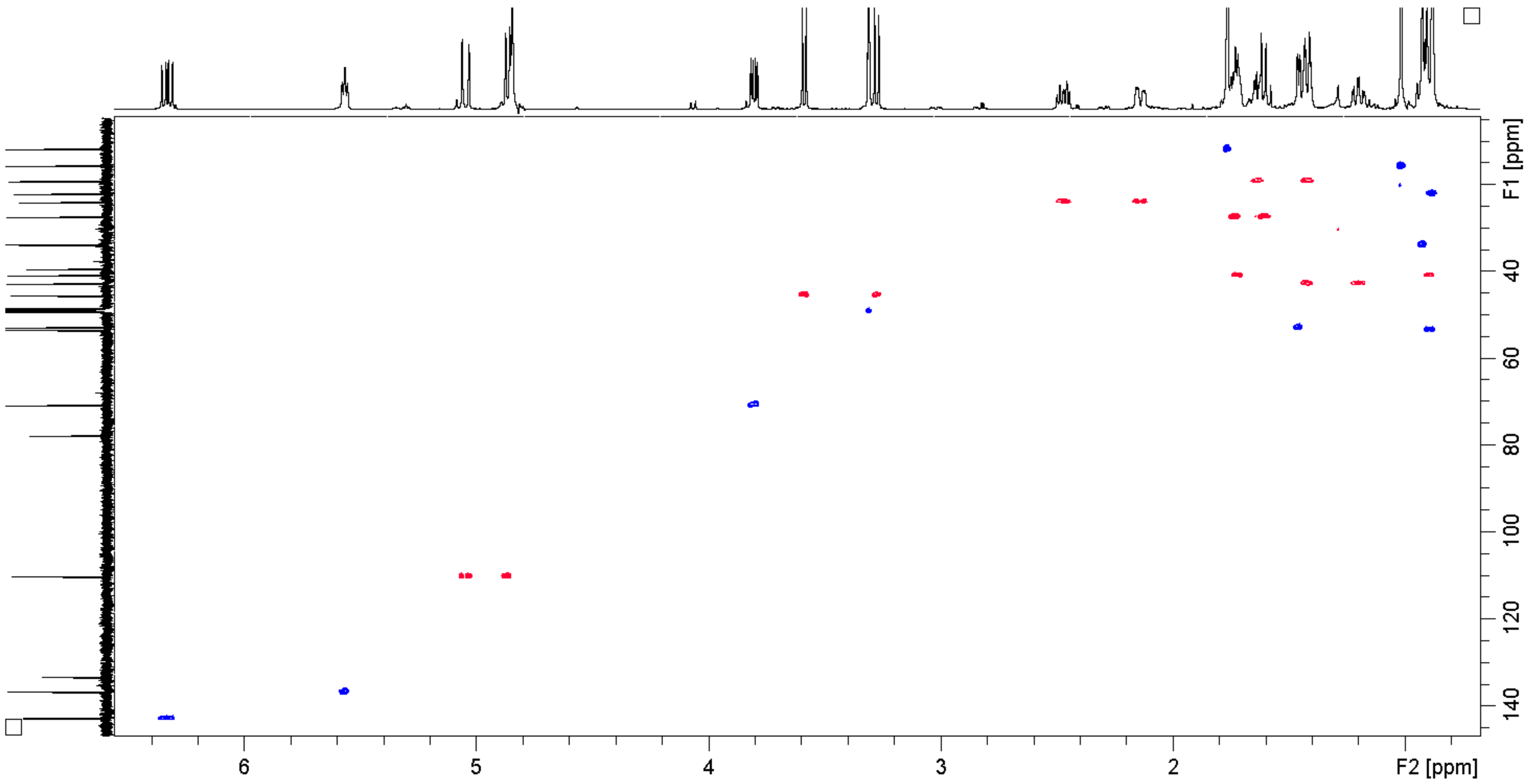

Figure S19. HSQC NMR spectrum of **2** (CD_3OD).

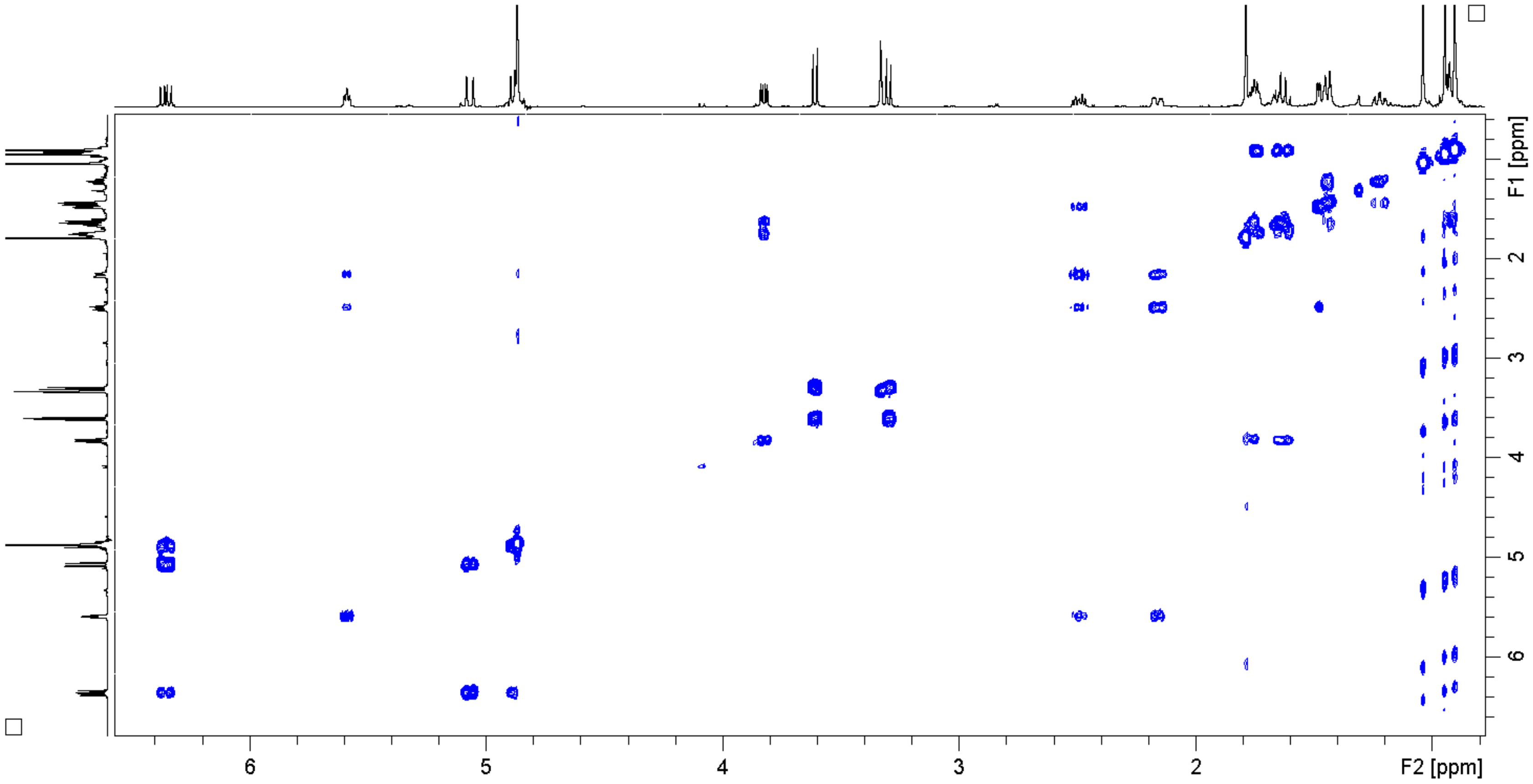

Figure S20. ^{1}H-^{1}H COSY NMR spectrum of **2** (CD_3OD).

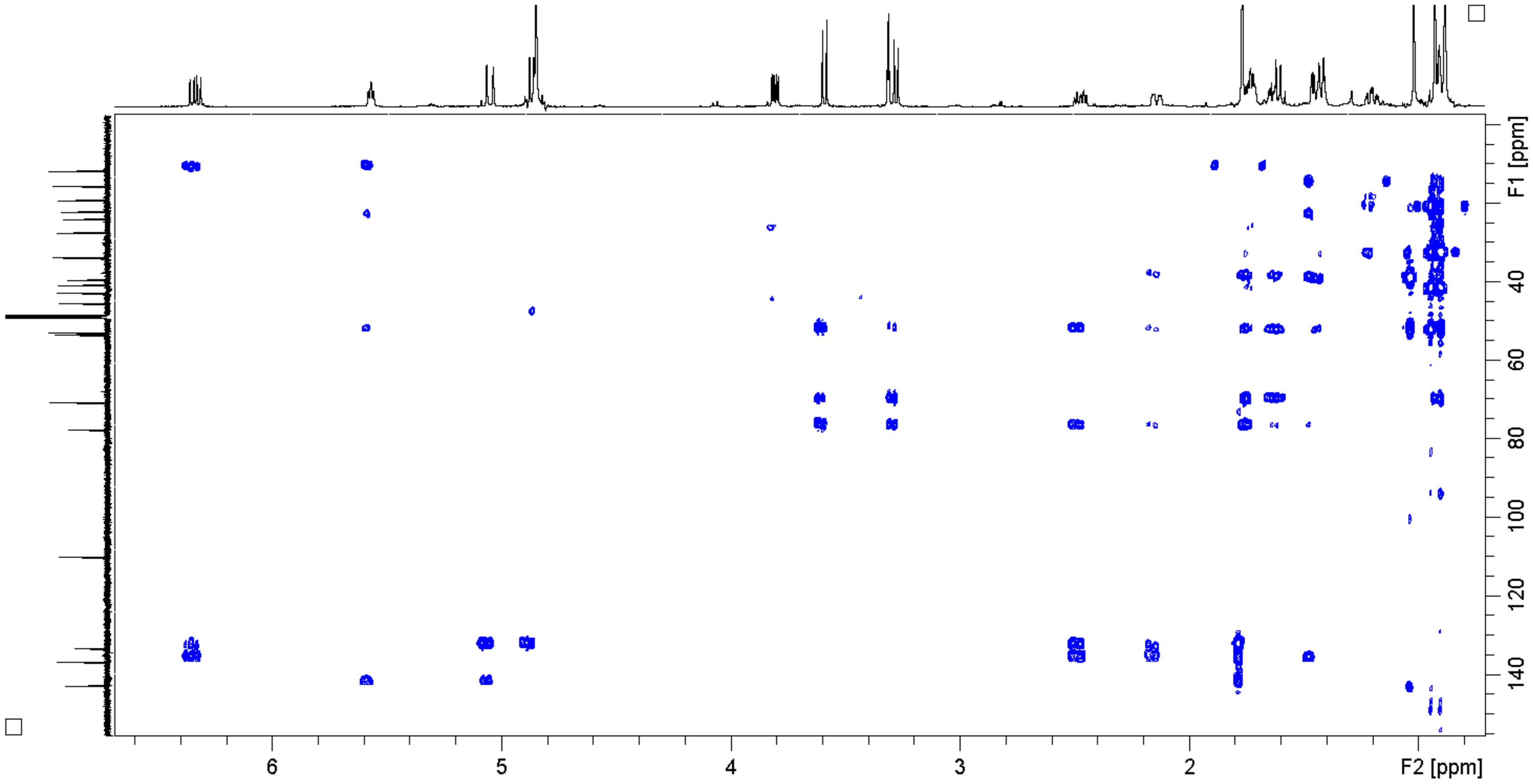

Figure S21. HMBC NMR spectrum of **2** (CD_3OD)..

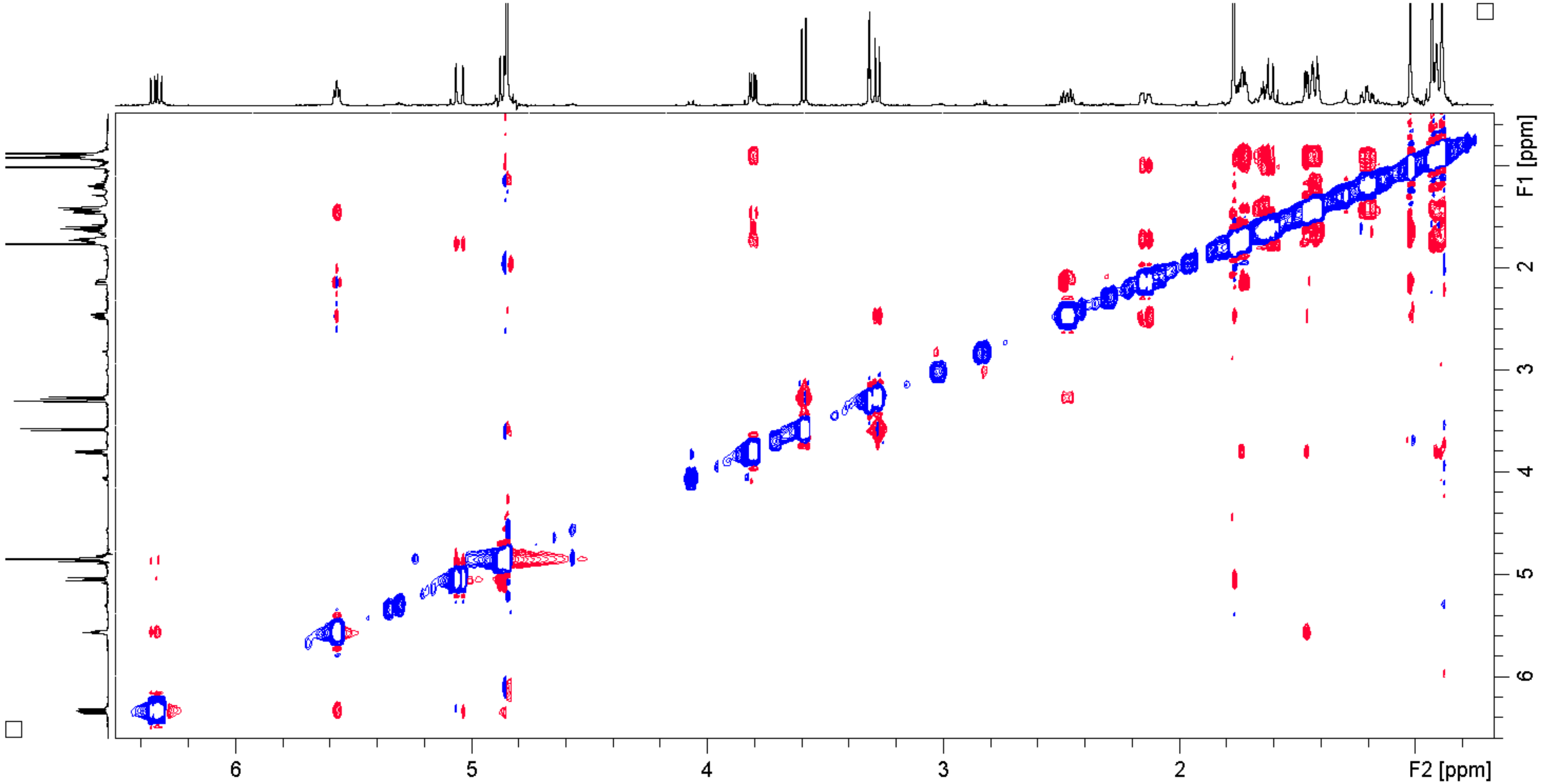

Figure S22. NOESY NMR spectrum of **2** (CD_3OD).

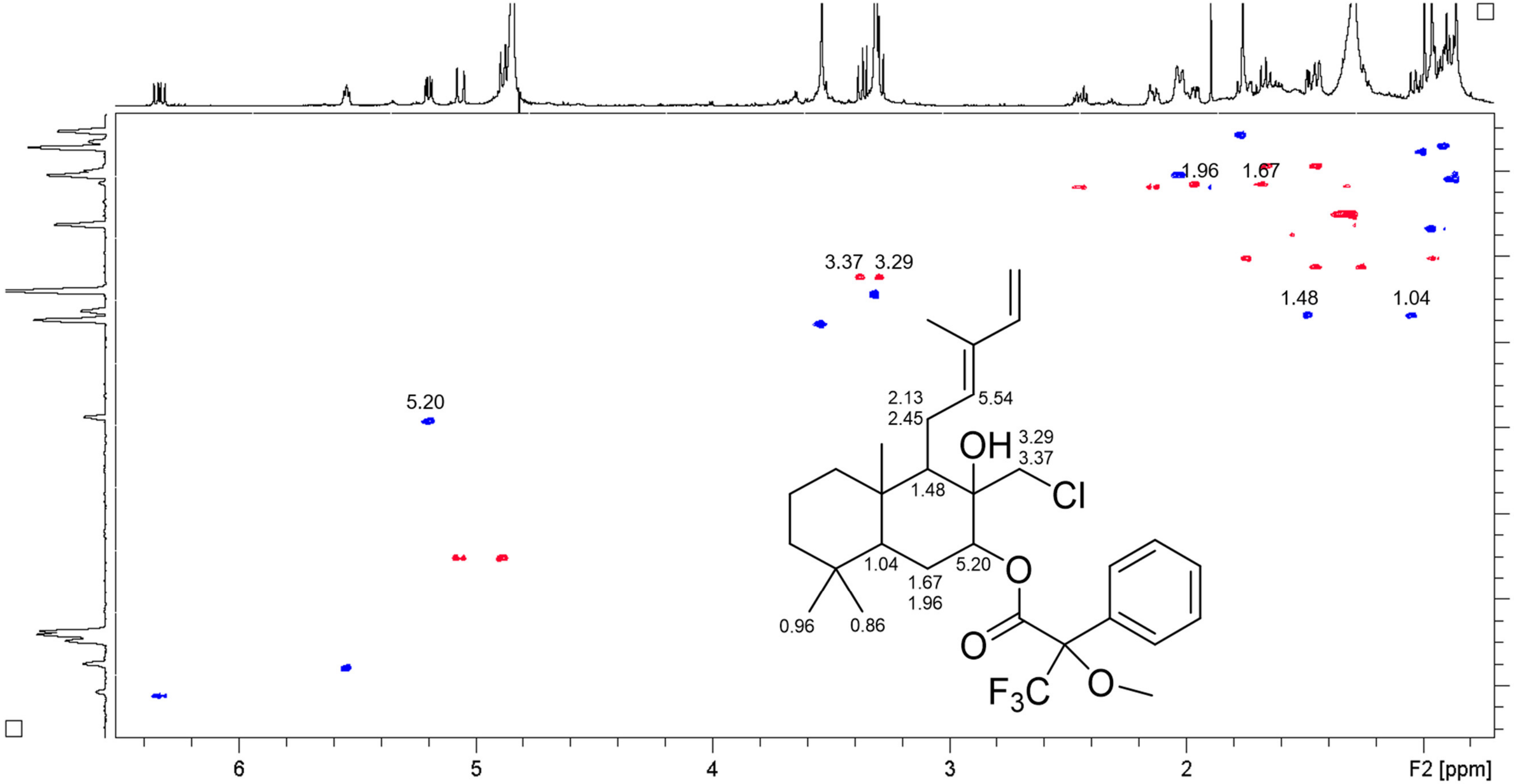

Figure S23. HSQC spectrum of (*S*)-MTPA esters of **2** (**2a**) (CD_3OD).

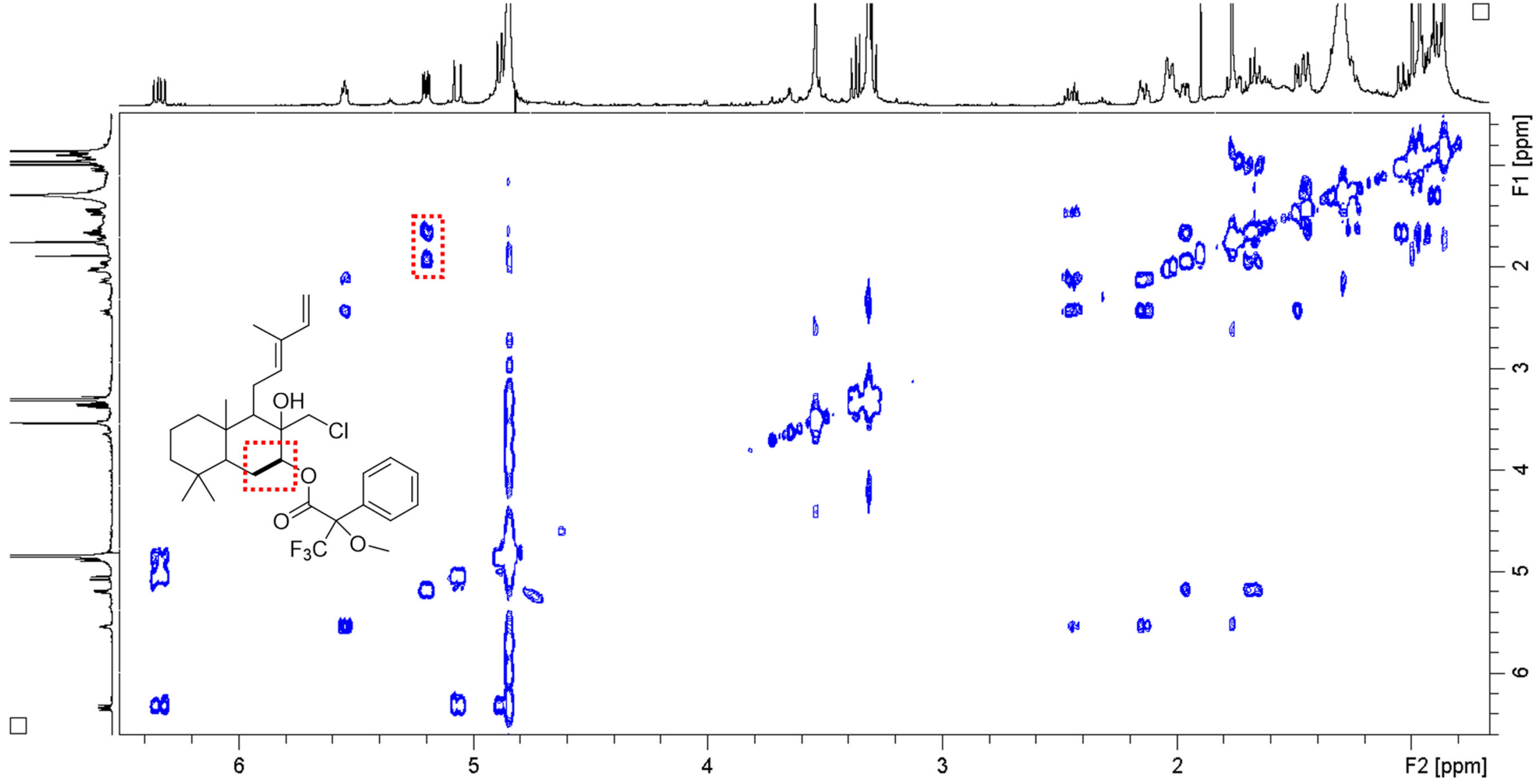

Figure S24. ^{1}H-^{1}H COSY spectrum of (*S*)-MTPA esters of **2** (**2a**) (CD_3OD).

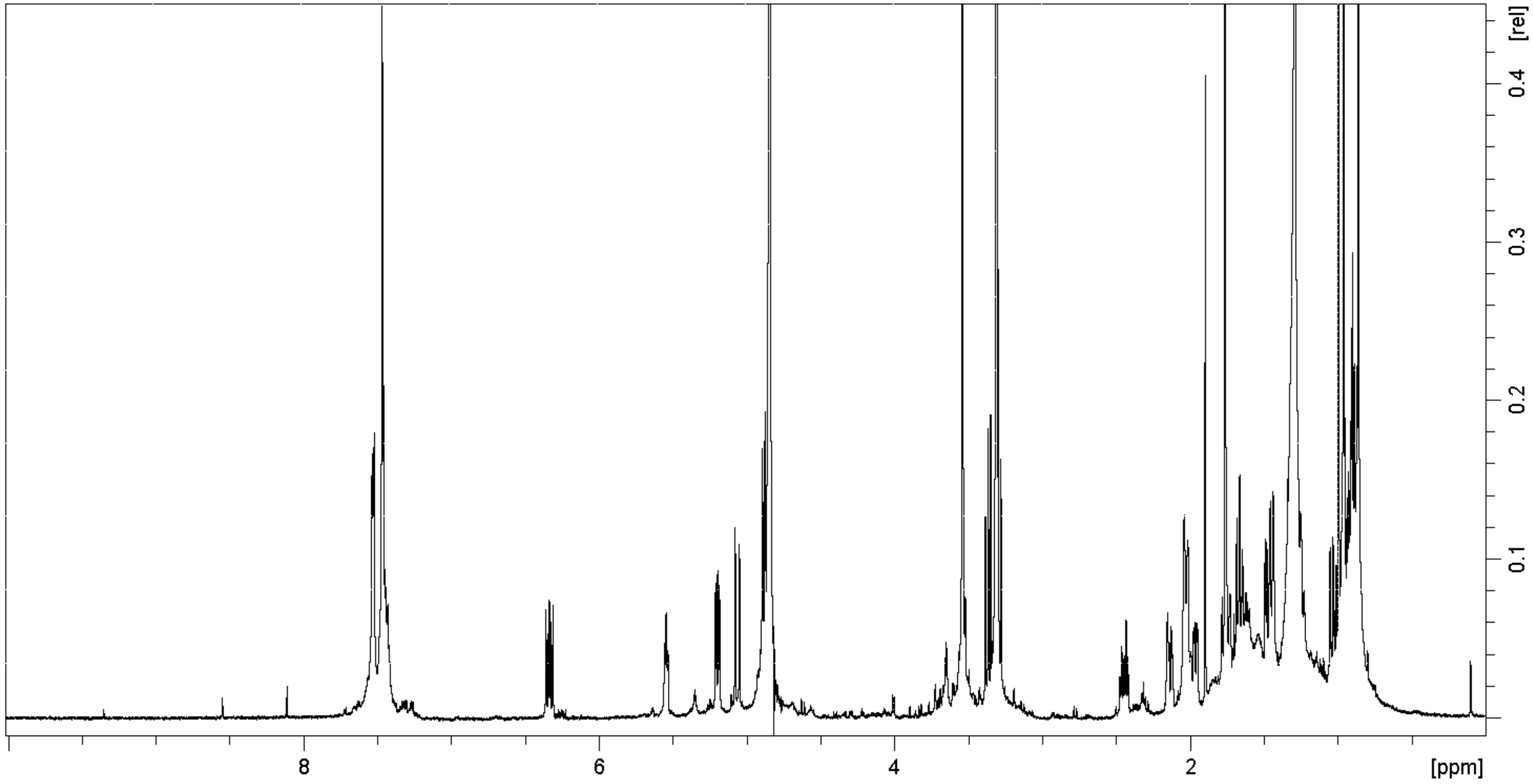

Figure S25. ^{1}H NMR spectrum of (*S*)-MTPA esters of **2** (**2a**) (CD_3OD).

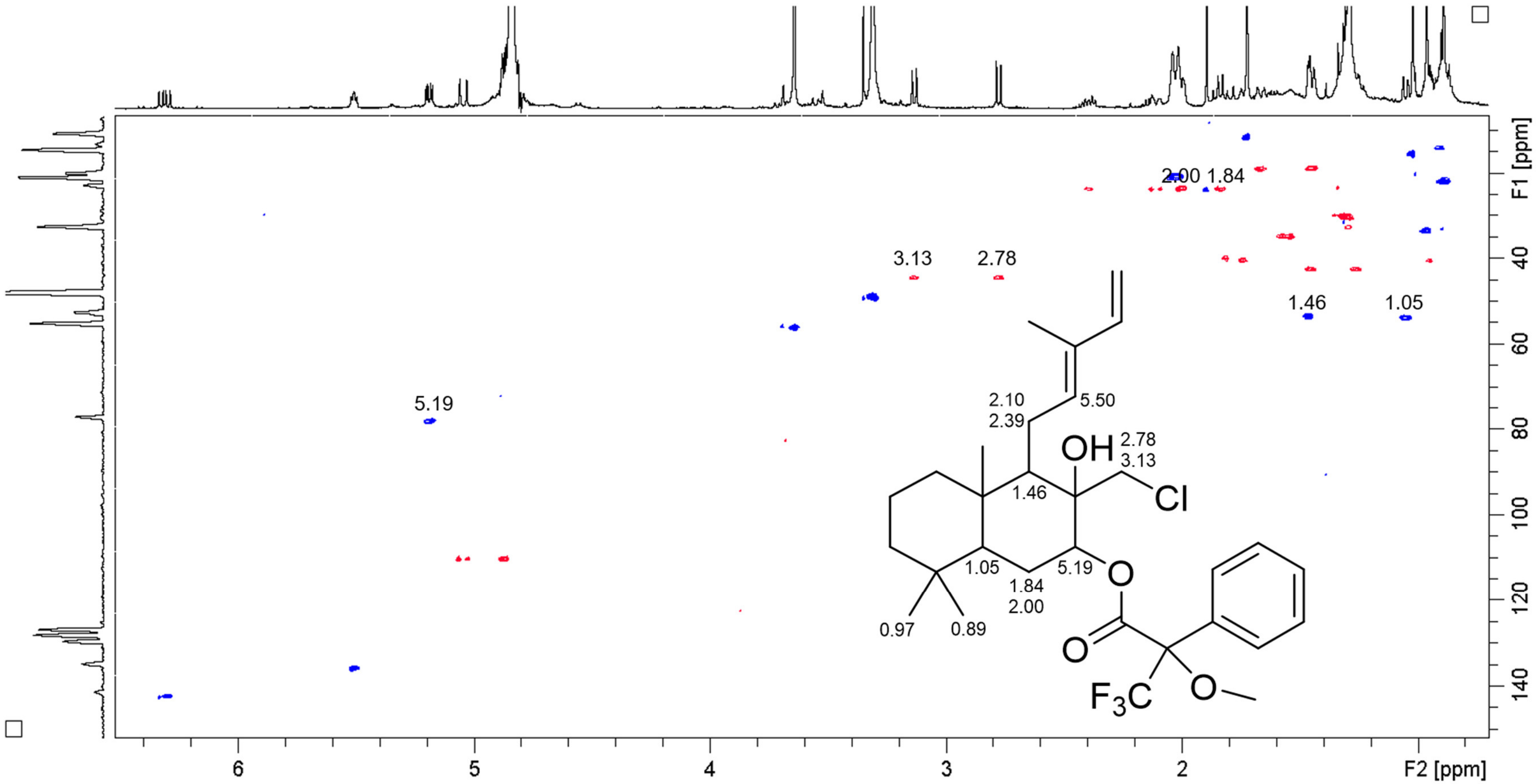

Figure S26. HSQC spectrum of (*R*)-MTPA esters of **2** (**2b**) (CD_3OD).

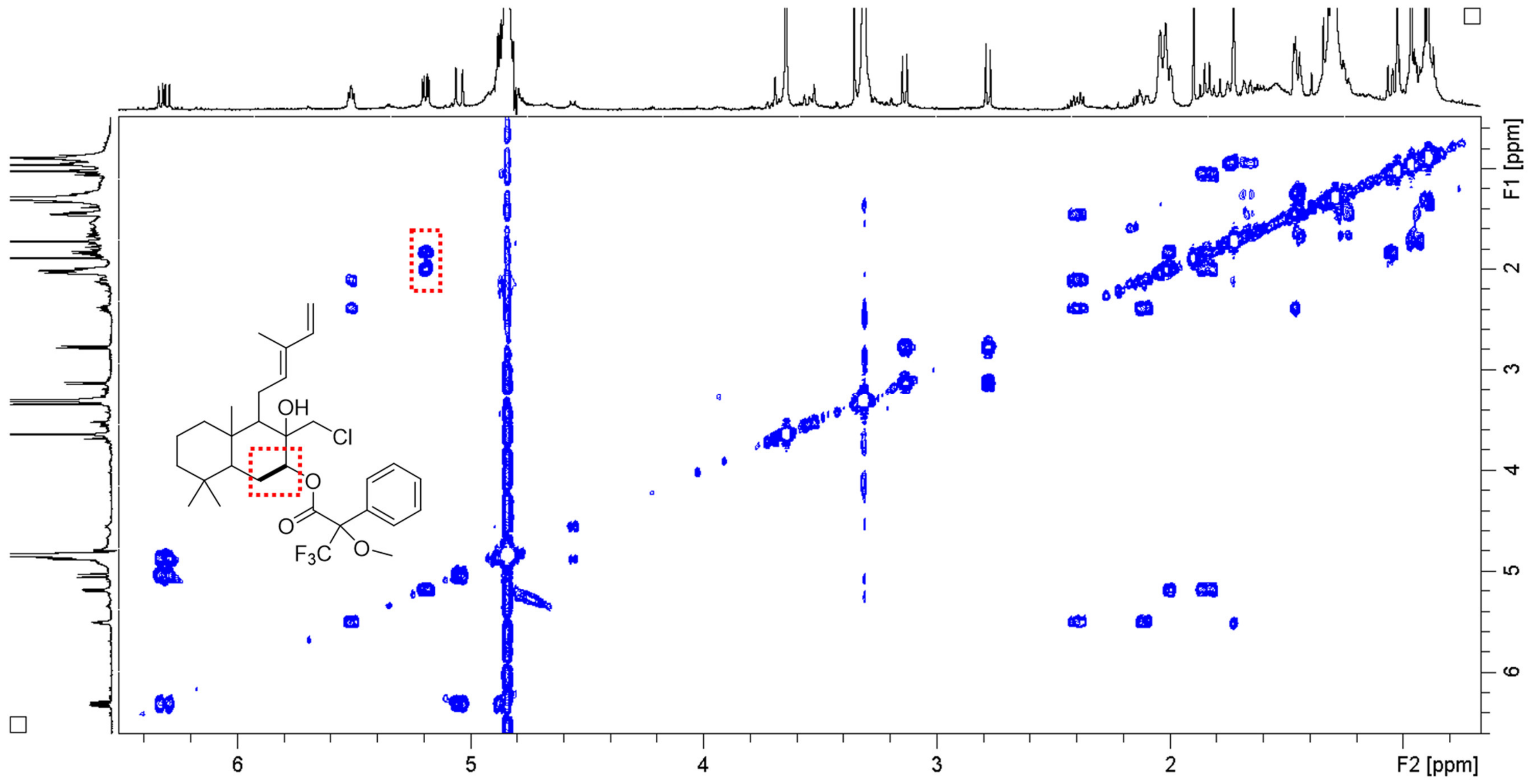

Figure S27. ^{1}H-^{1}H COSY spectrum of (*R*)-MTPA esters of **2** (**2b**) (CD3OD).

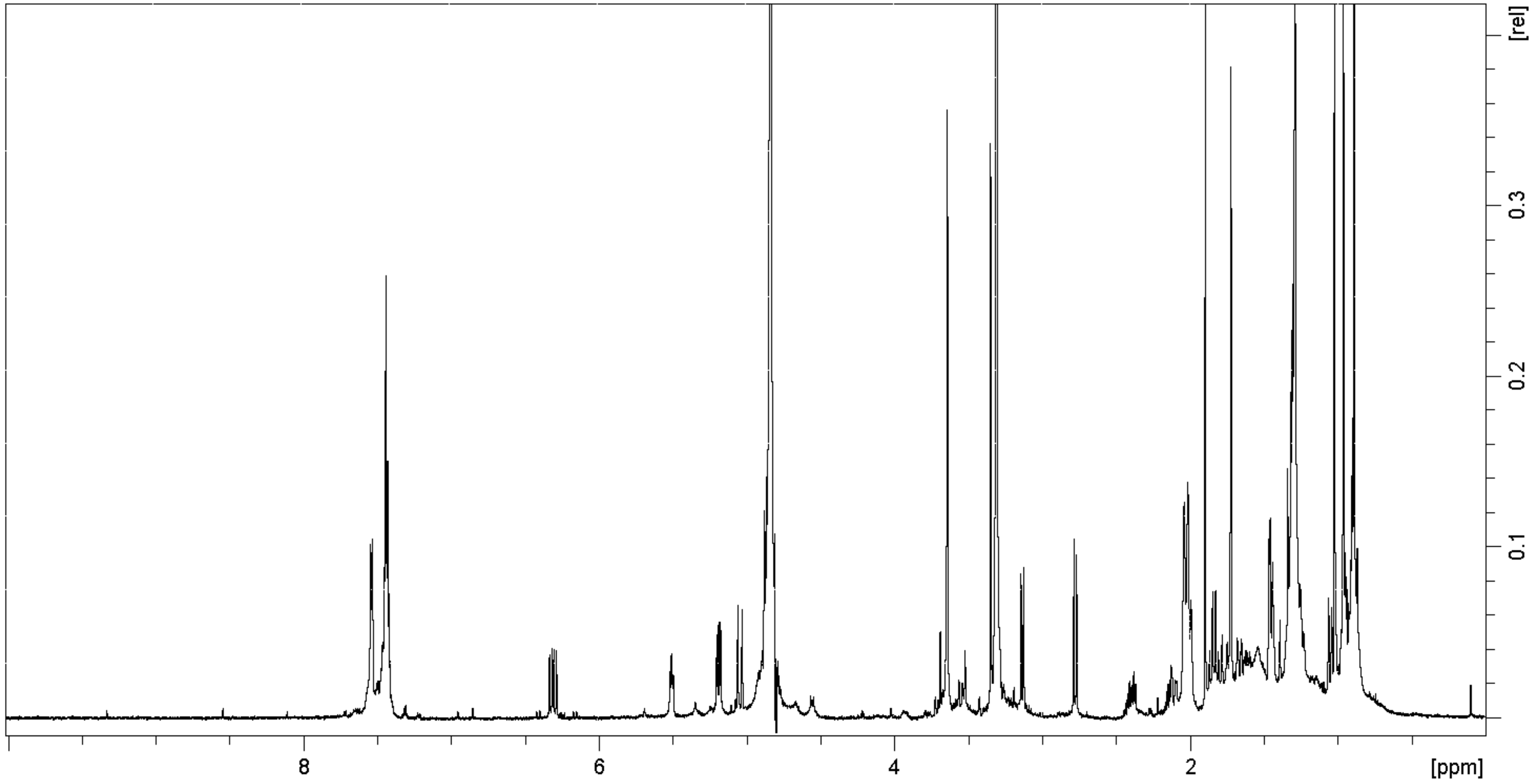

Figure S28. ^{1}H NMR spectrum of (*R*)-MTPA esters of **2** (**2b**) (CD_3OD).

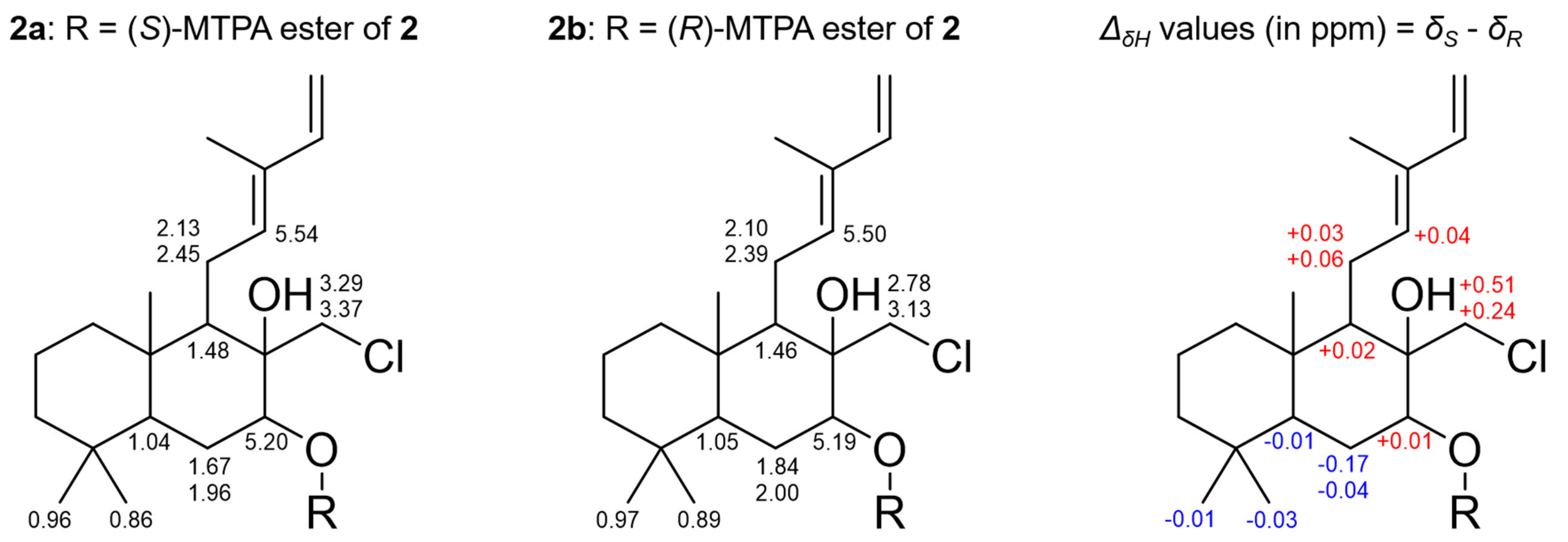

Figure S29. Summarized Δδ values (in ppm) = δ_S - δ_R obtained for (*S*)- and (*R*)-MPTA esters of **2**.

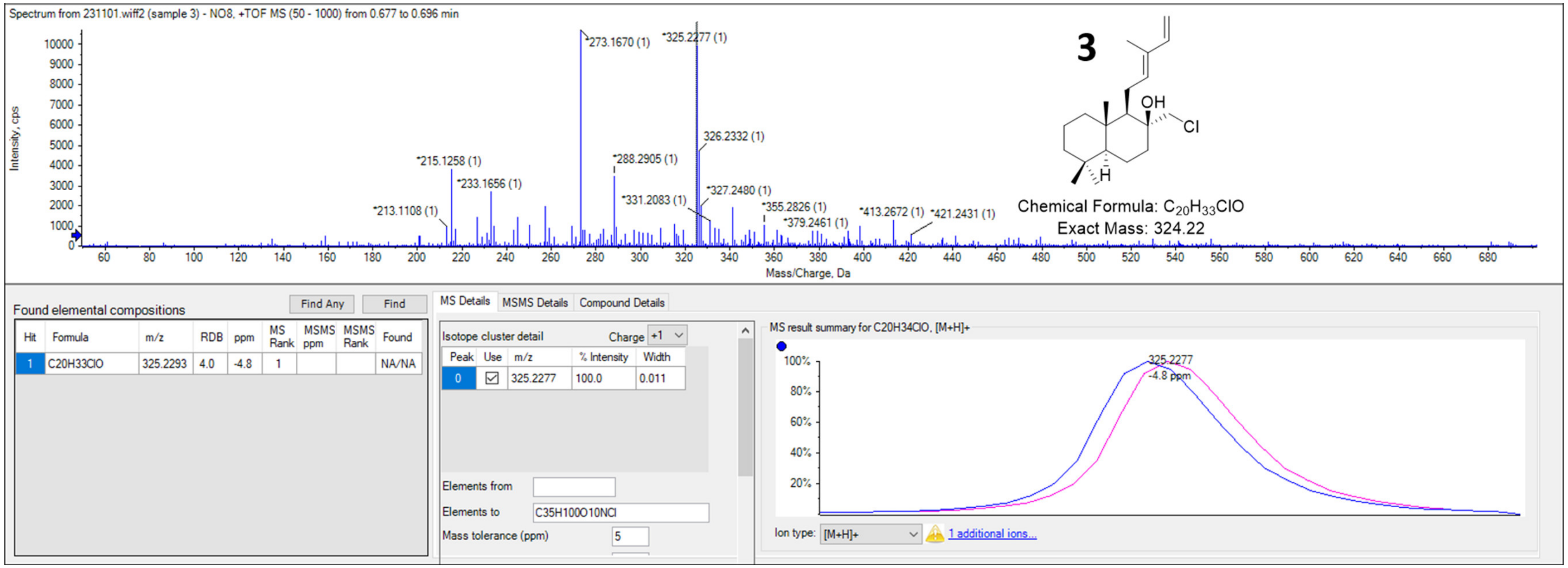

Figure S30. HRESIMS data of **3**.

.

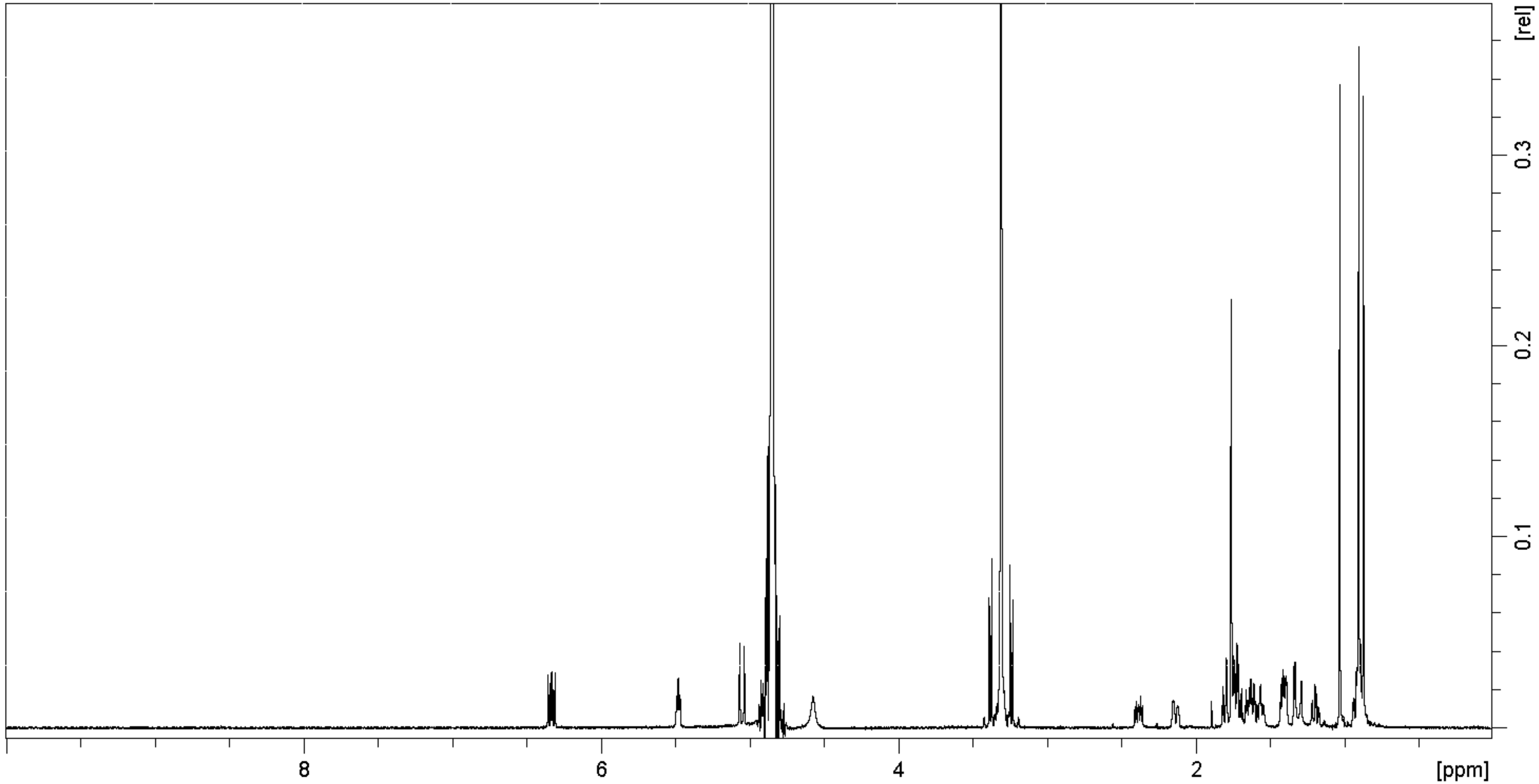

Figure S31. ^{1}H NMR spectrum of **3** (CD_3OD).

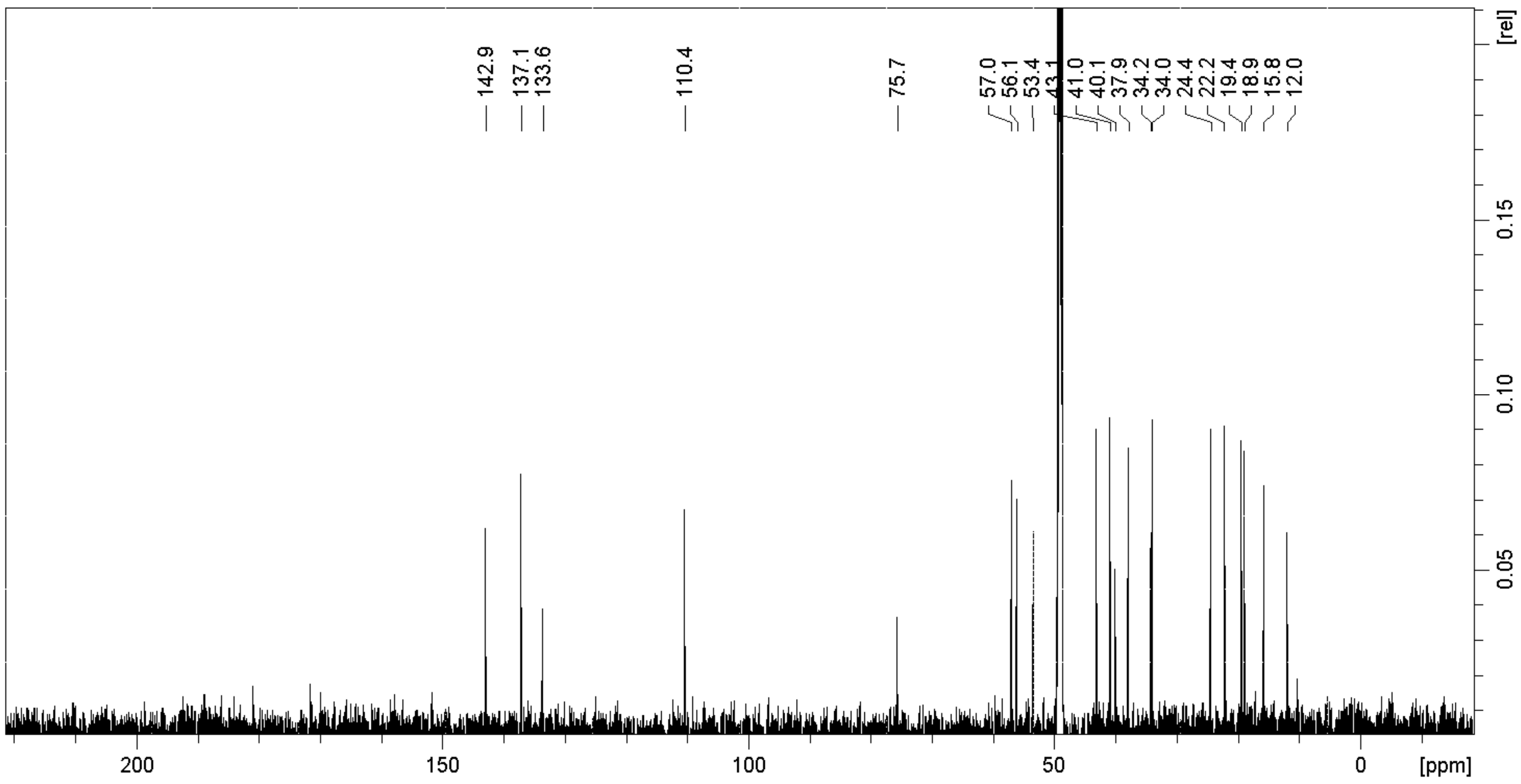

Figure S32. ^{13}C NMR spectrum of **3** (CD_3OD).

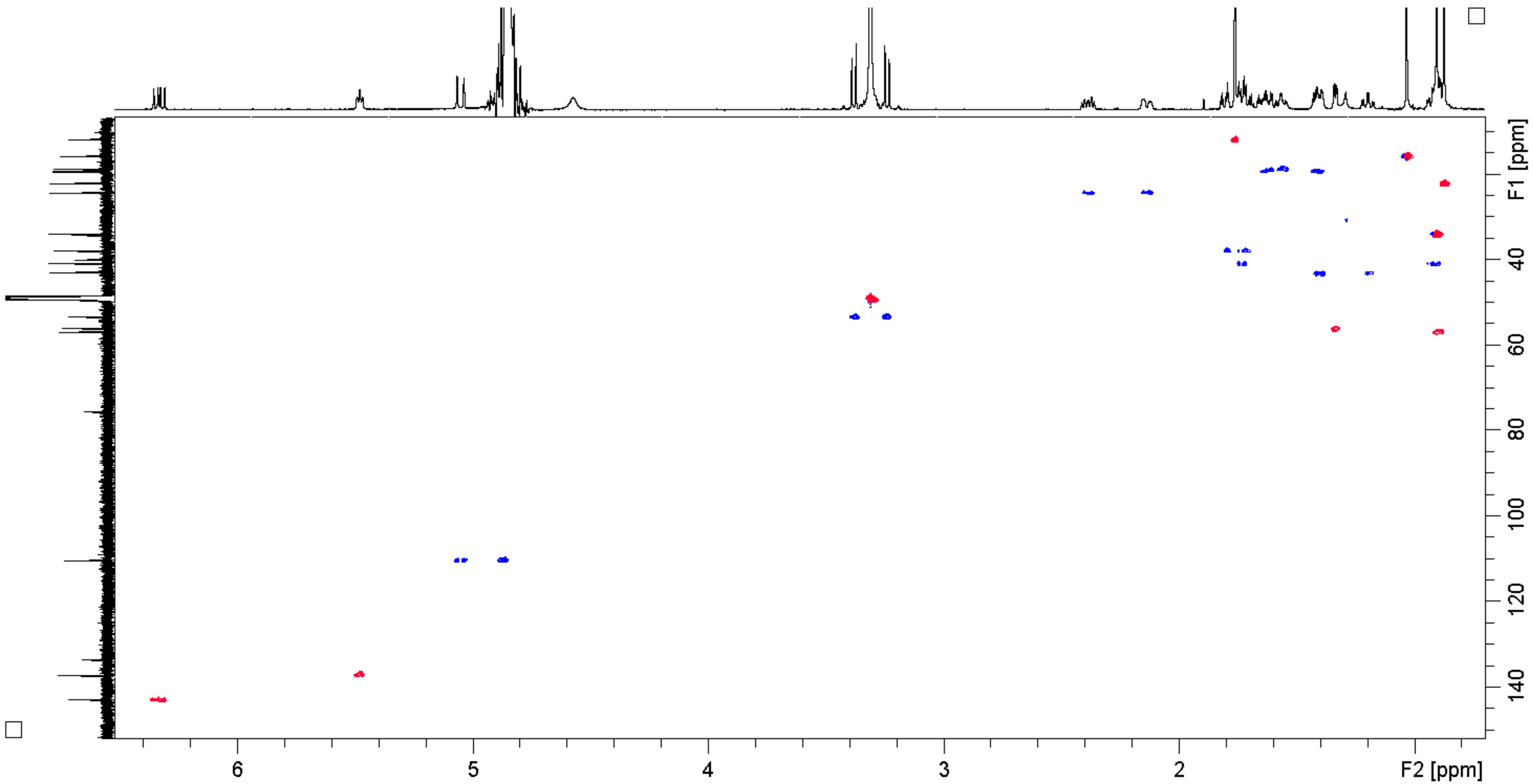

Figure S33. HSQC NMR spectrum of **3** (CD_3OD).

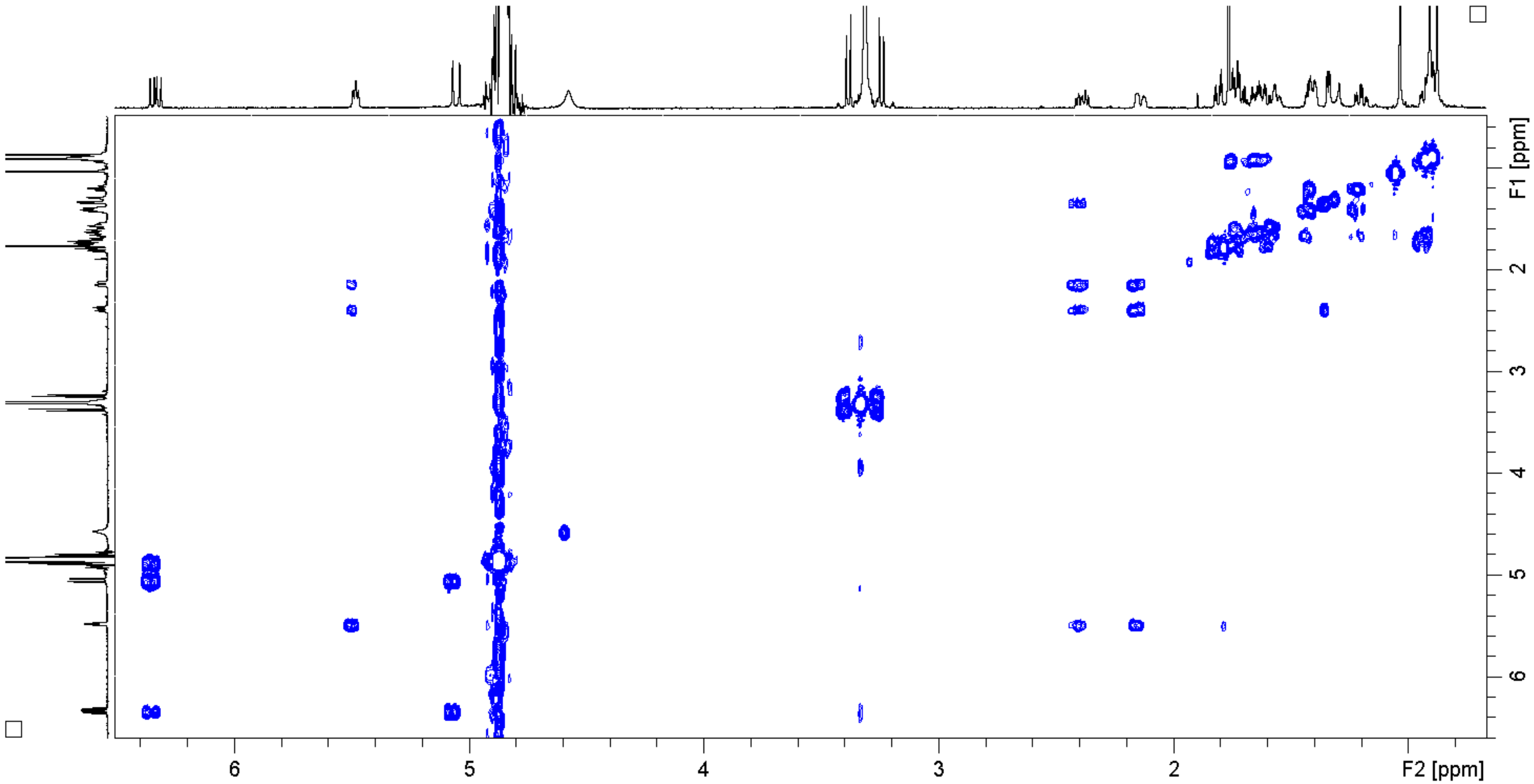

Figure S34. ^{1}H-^{1}H COSY NMR spectrum of **3** (CD_3OD).

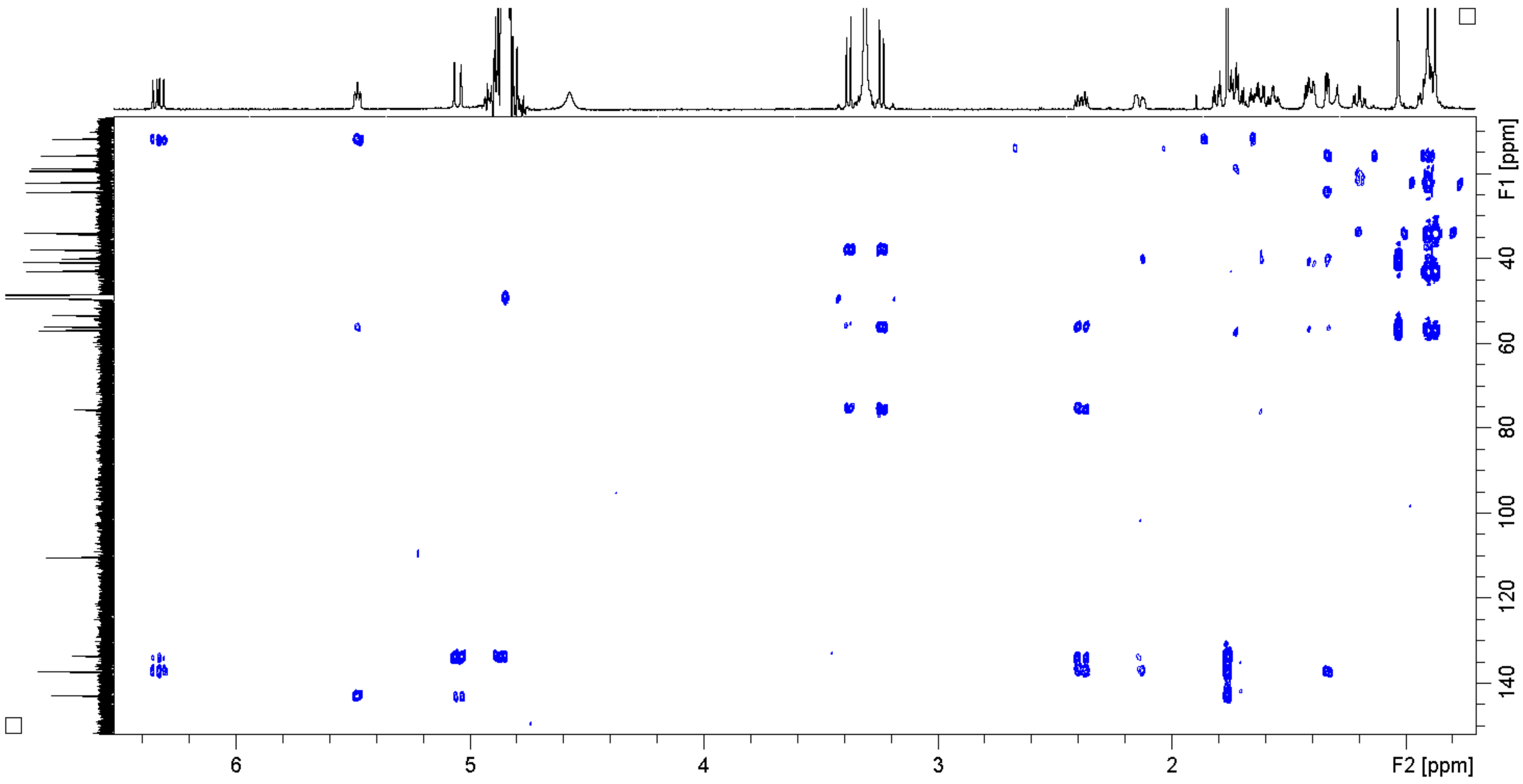

Figure S35. HMBC NMR spectrum of **3** (CD_3OD).

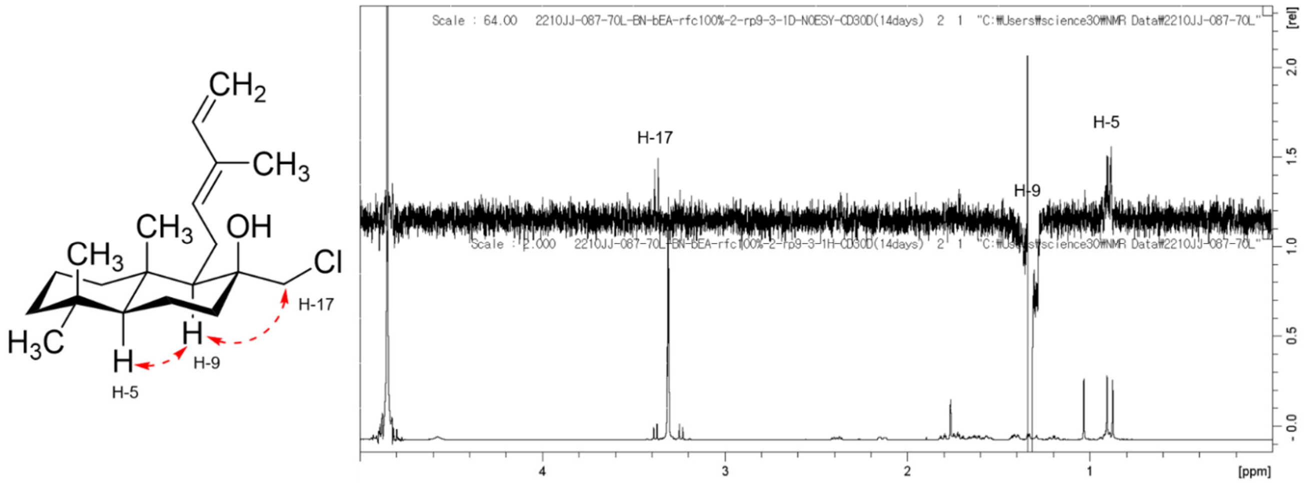

Figure S36. 1D selective NOESY data of **3** (irradiated H-9) (CD_3OD).

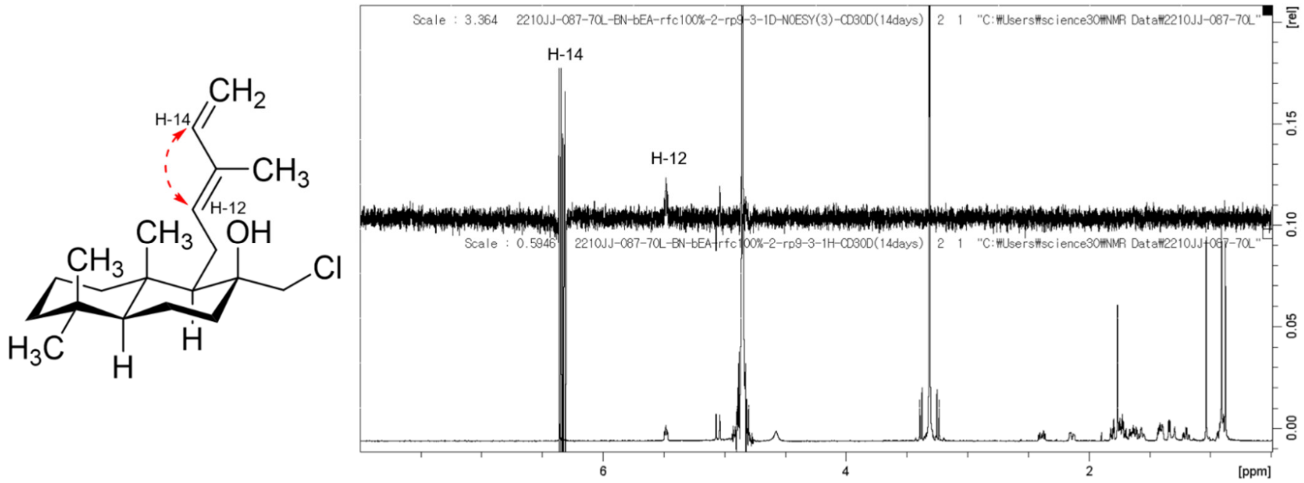

Figure S37. 1D selective NOESY data of **3** (irradiated H-14) (CD_3OD).

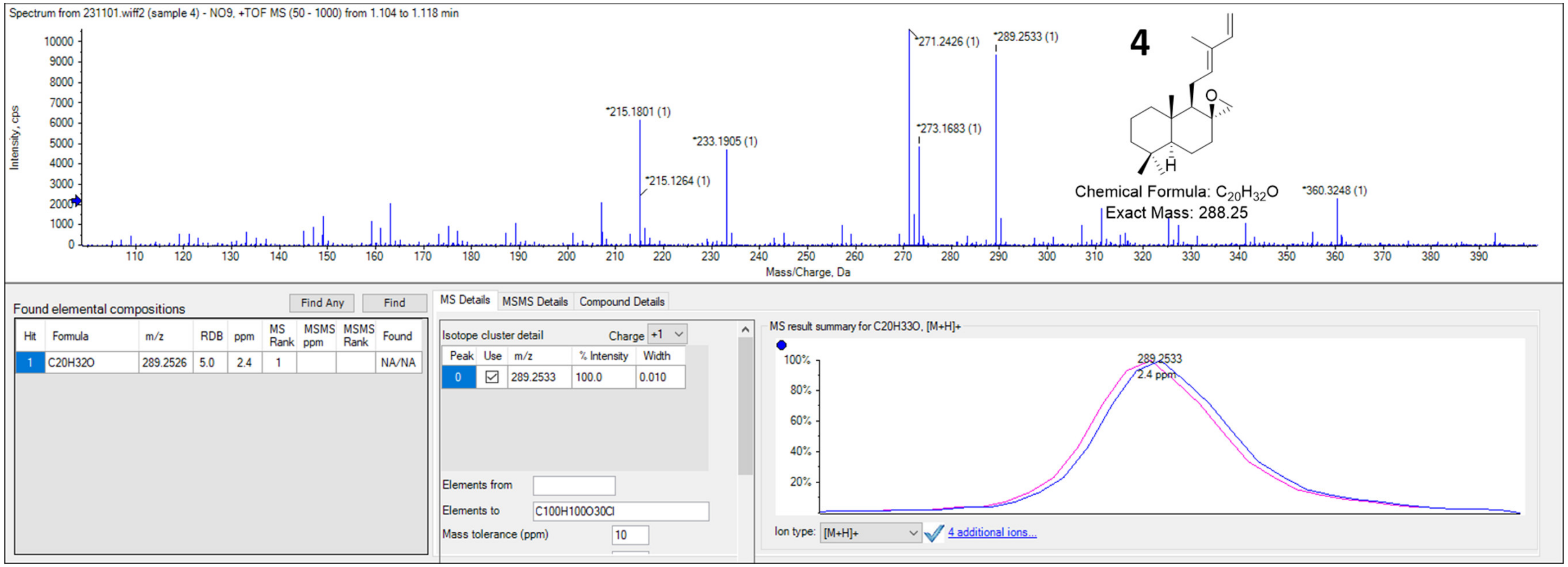

Hit	Formula	m/z	RDB	ppm	MS Rank	MSMS ppm	MSMS Rank	Found
1	C20H32O	289.2526	5.0	2.4	1			NA/NA

Peak	Use	m/z	% Intensity	Width
0	☑	289.2533	100.0	0.010

Figure S38. HRESIMS data of **4**.

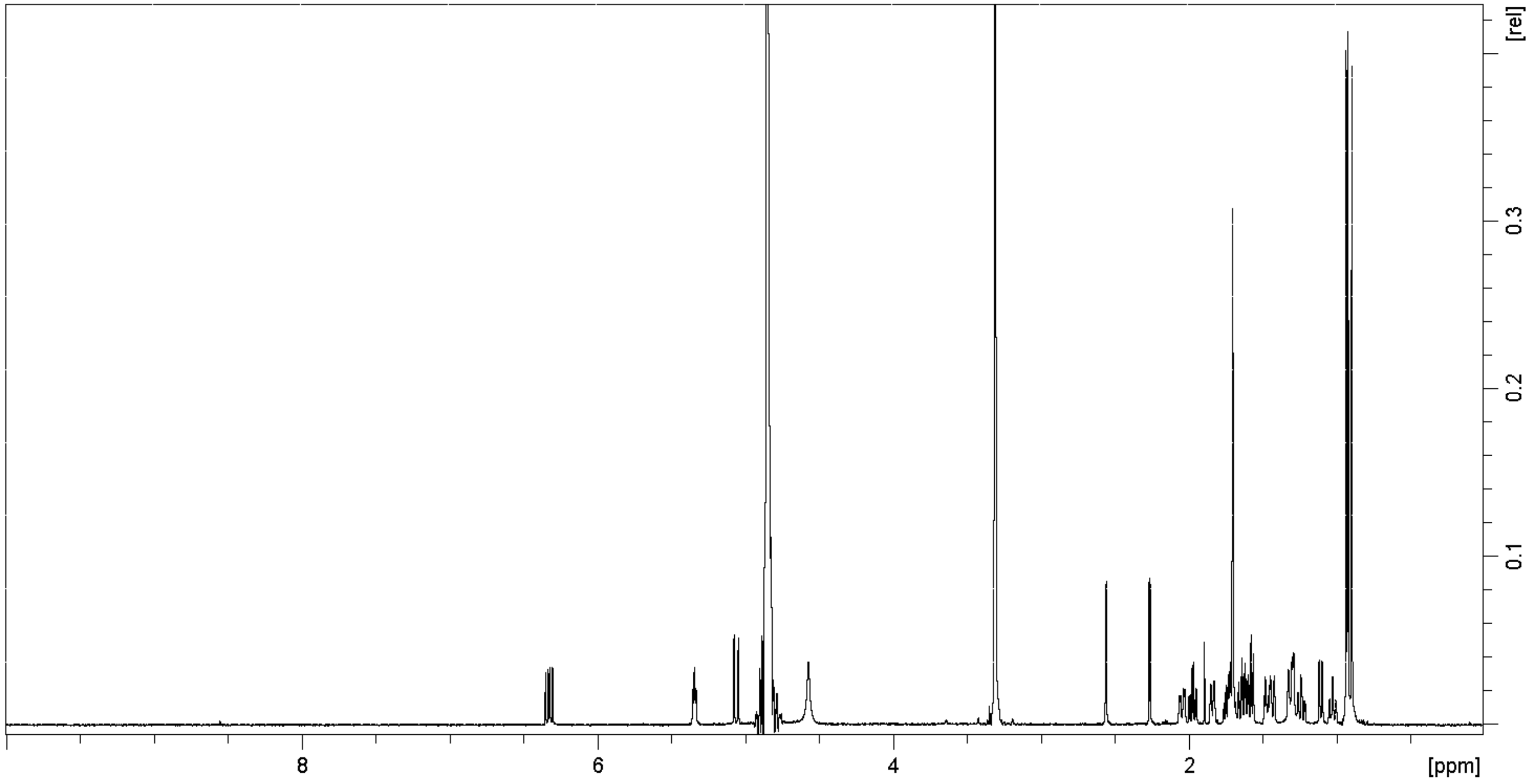
[rel]
0.3
0.2
0.1
8
6
4
2
[ppm]

Figure S39. ^{1}H NMR spectrum of **4** (CD_3OD).

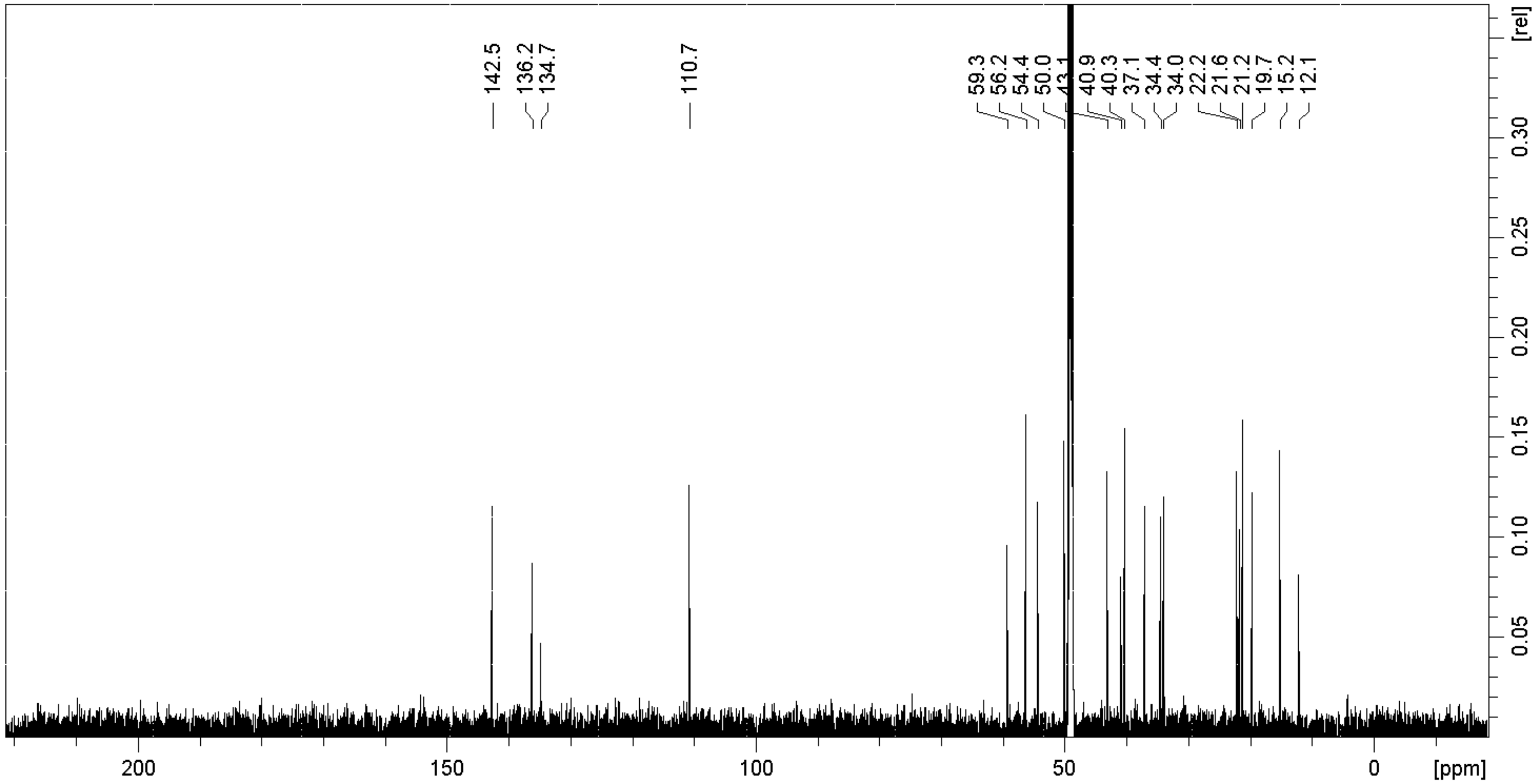

Figure S40. ^{13}C NMR spectrum of **4** (CD_3OD).

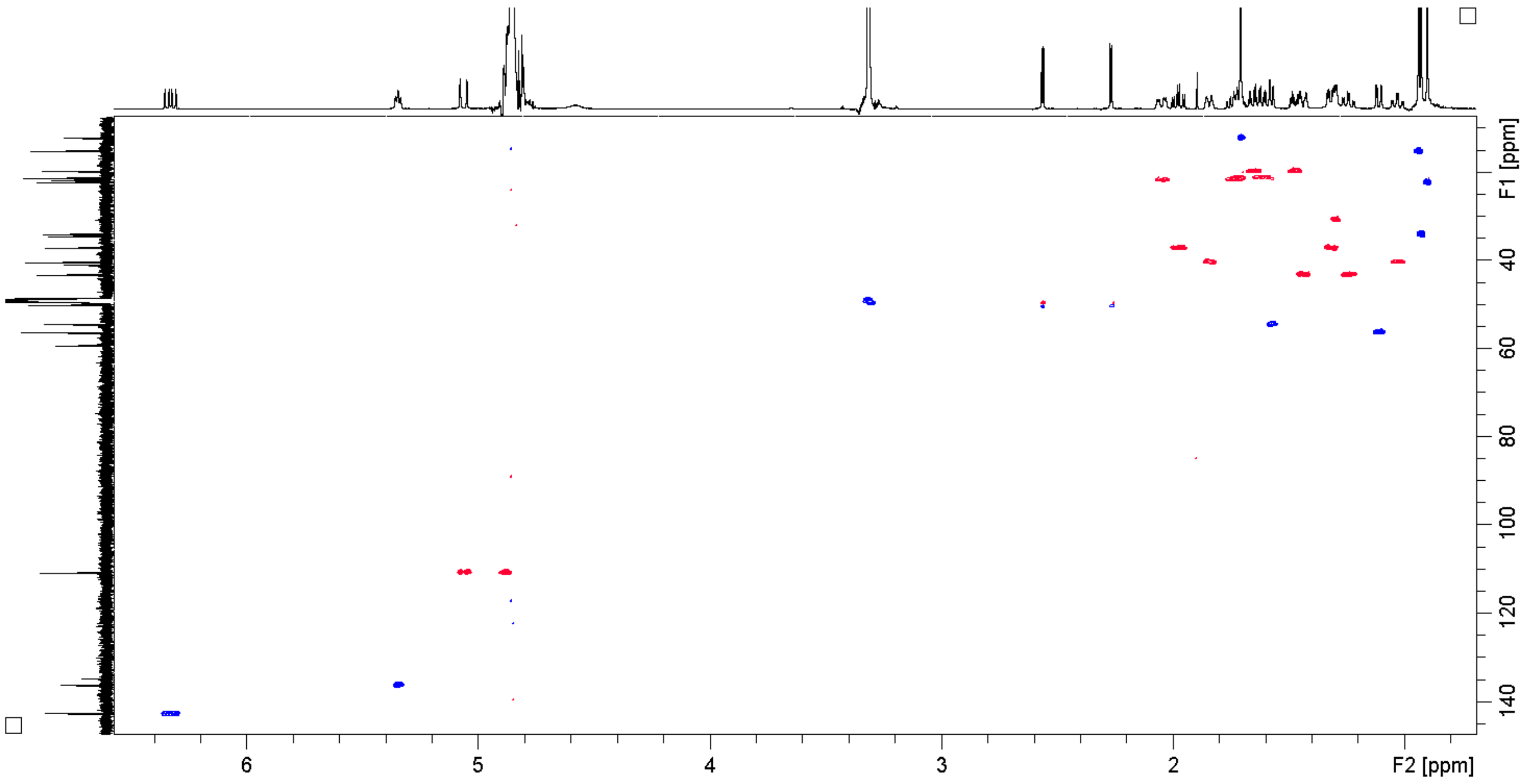

Figure S41. HSQC NMR spectrum of **4** (CD_3OD).

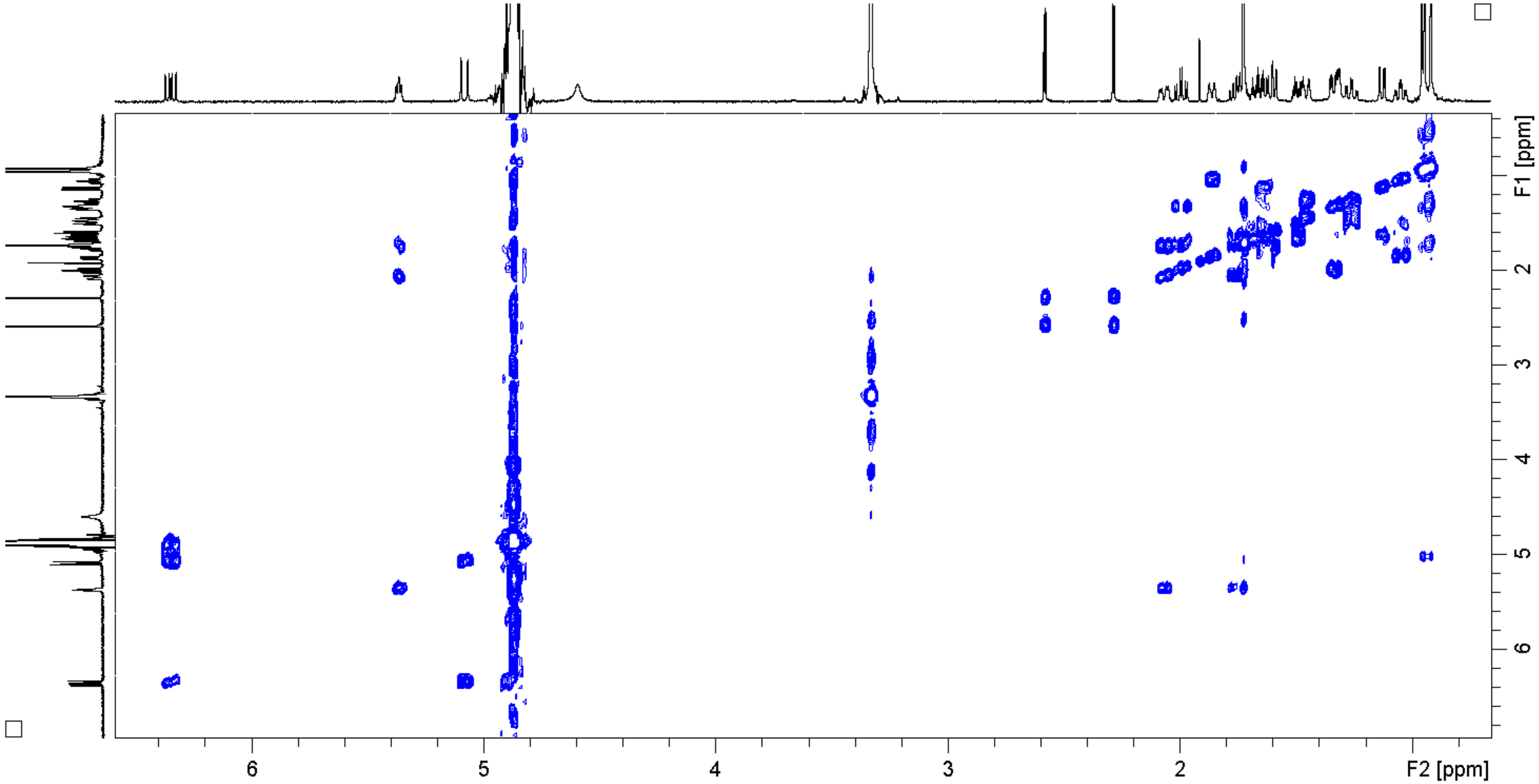

Figure S42. ^{1}H-^{1}H COSY NMR spectrum of **4** (CD_3OD).

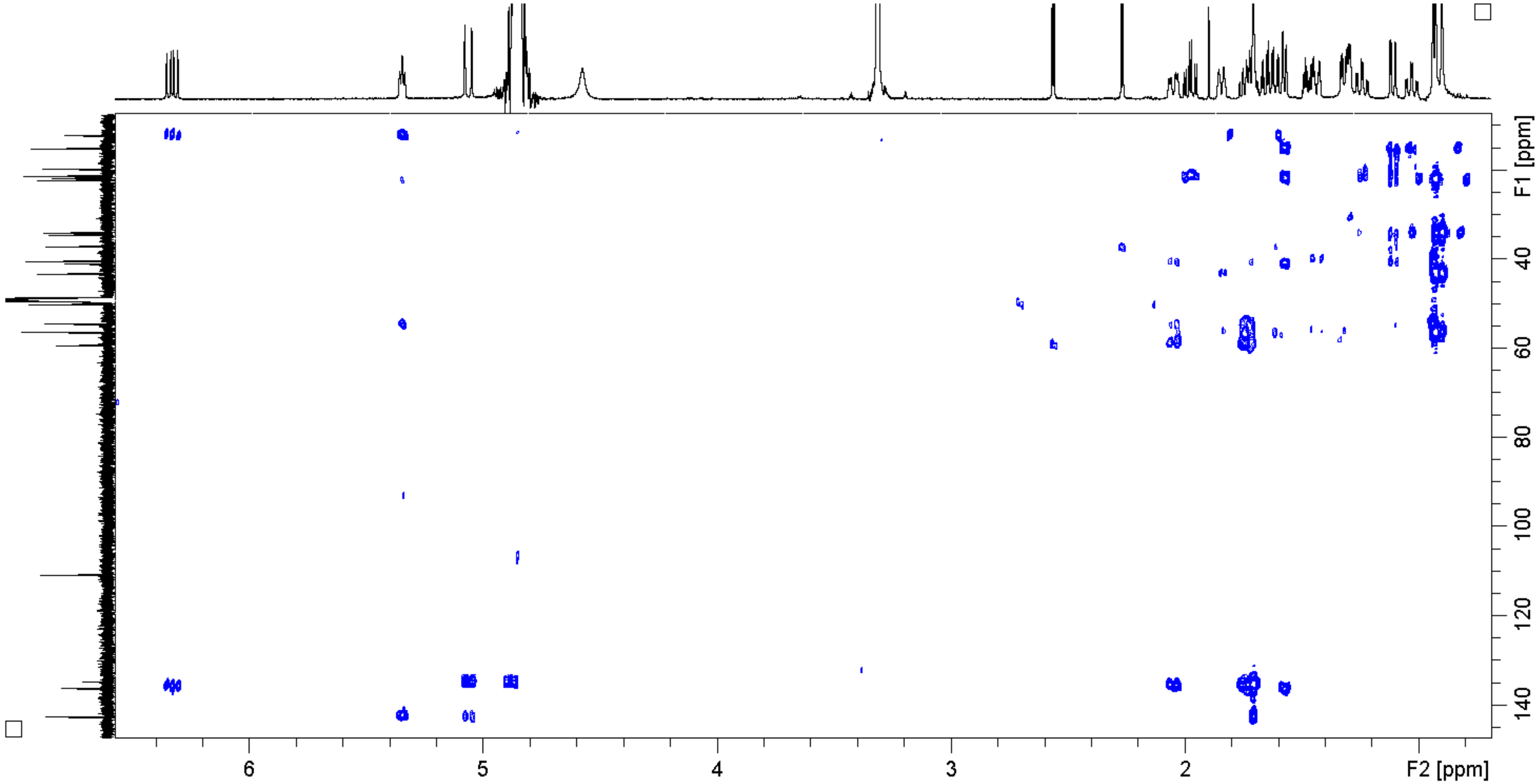

Figure S43. HMBC NMR spectrum of **4** (CD_3OD).

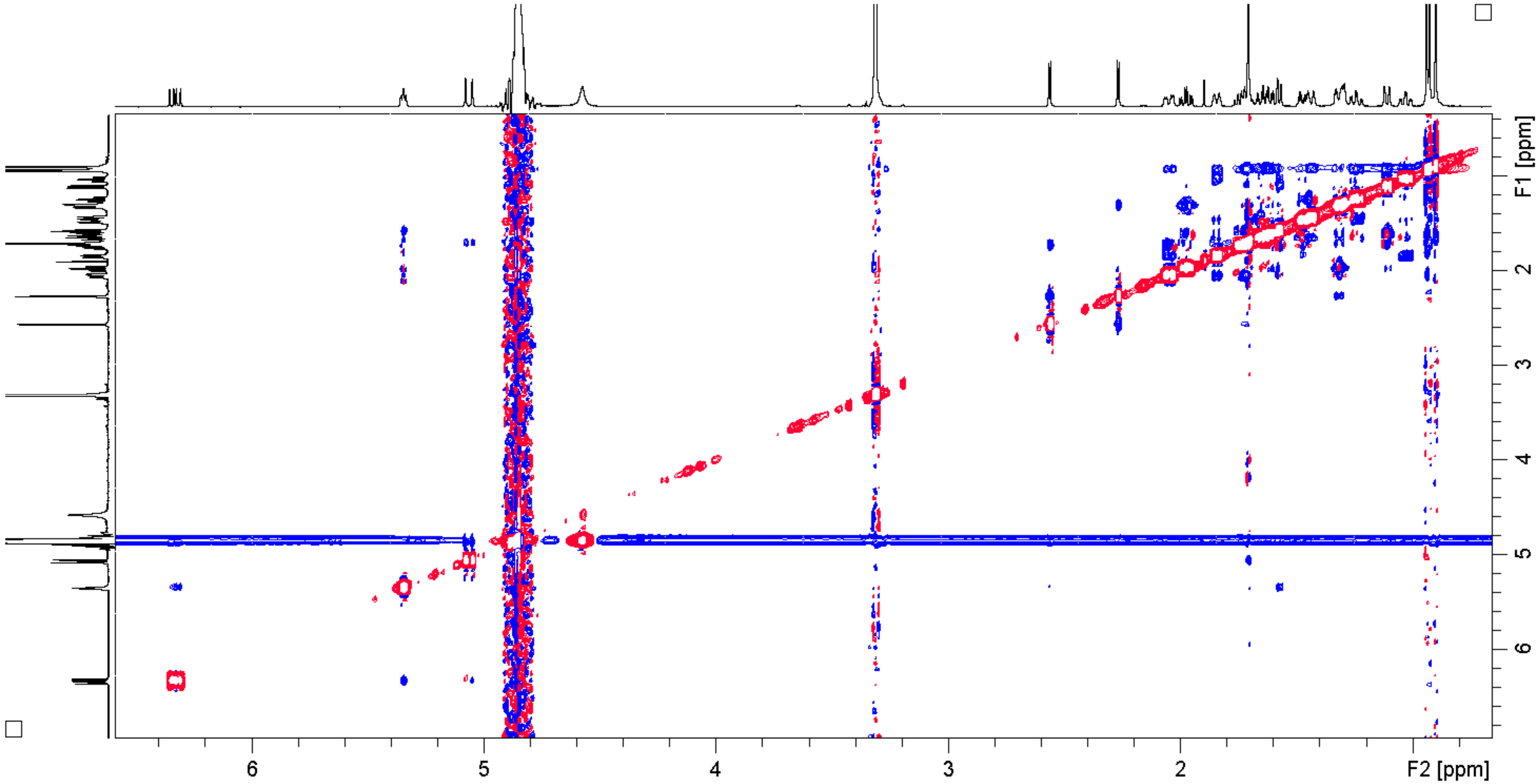
F1 [ppm]
F2 [ppm]
6
5
4
3
2

Figure S44. NOESY NMR spectrum of **4** (CD_3OD).

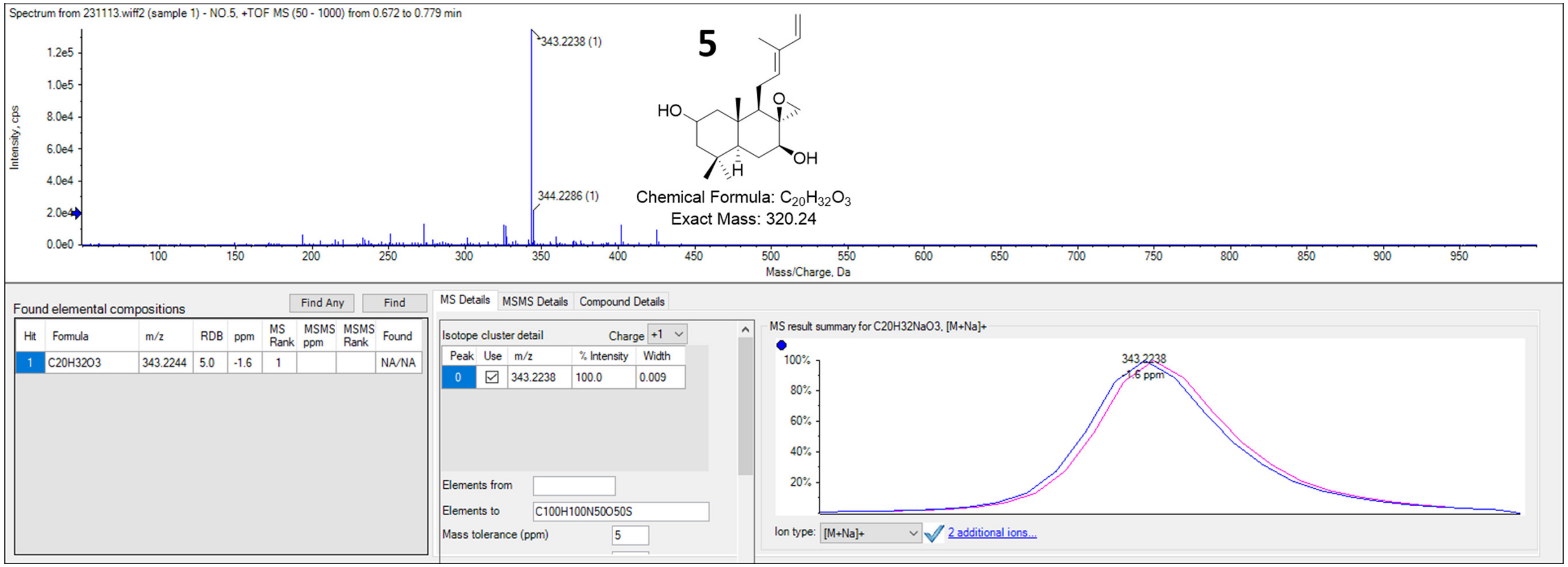

Figure S45. HRESIMS data of **5**.

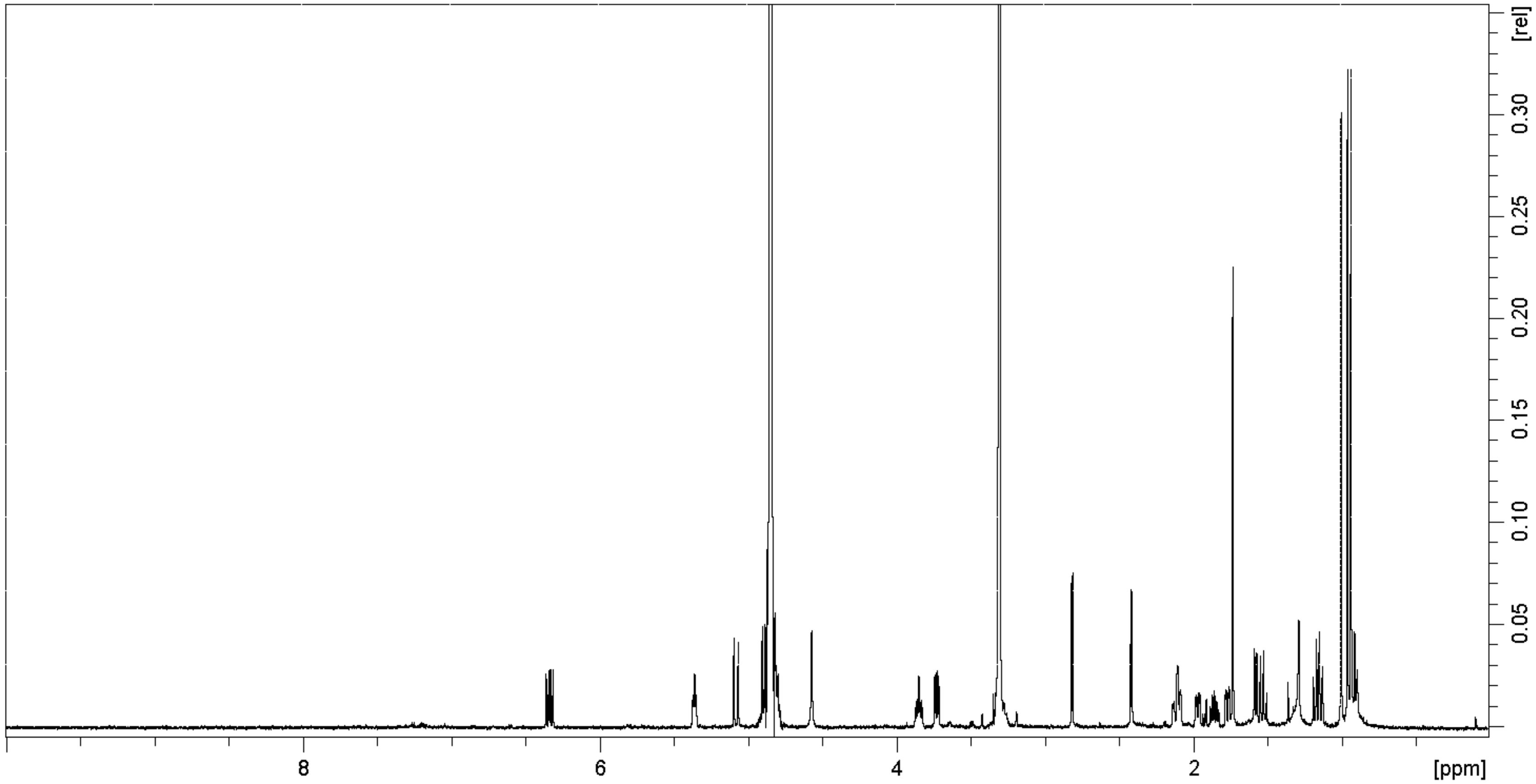

[rel]
0.30
0.25
0.20
0.15
0.10
0.05
8
6
4
2
[ppm]

Figure S46. ^{1}H NMR spectrum of **5** (CD_3OD).

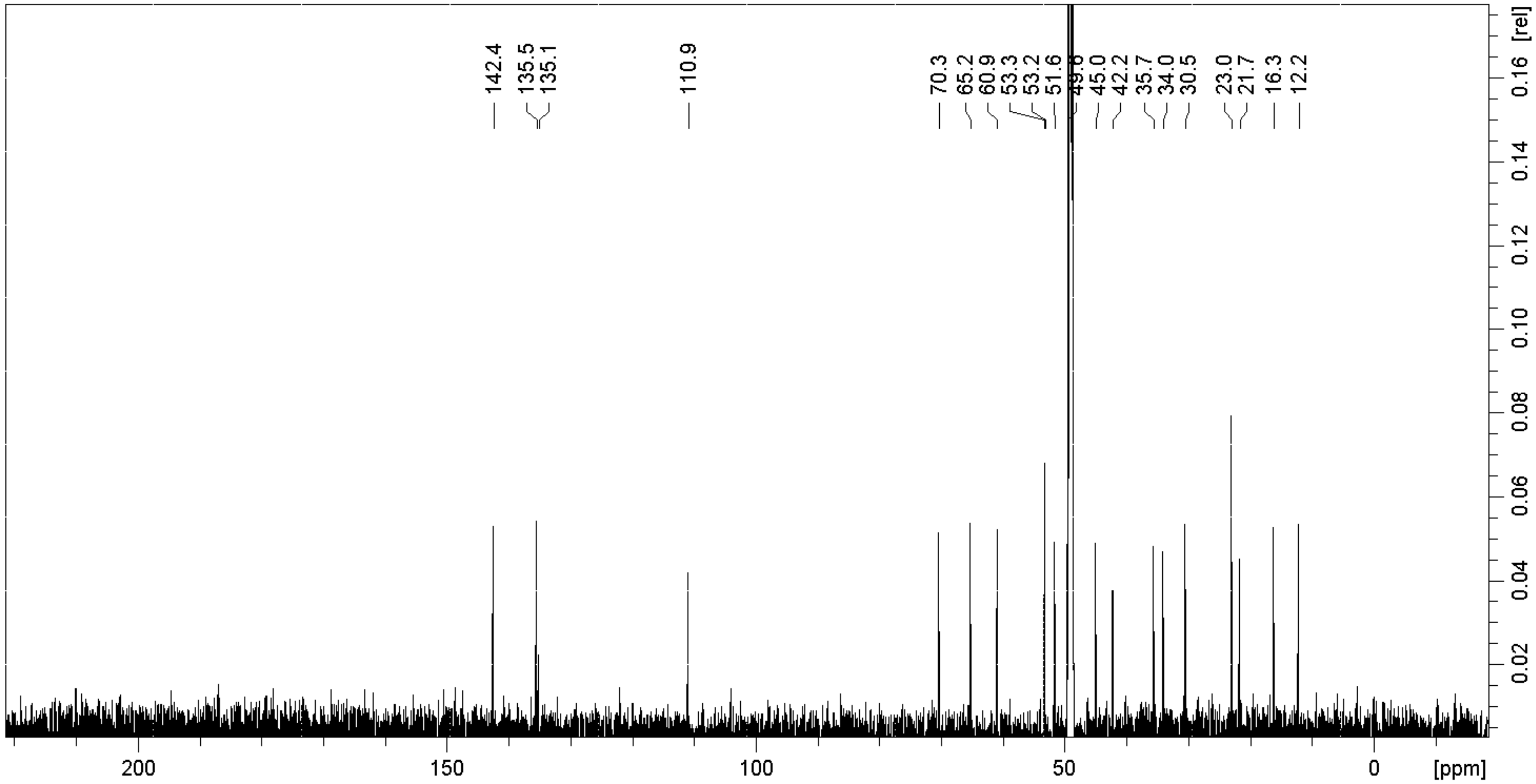

Figure S47. ^{13}C NMR spectrum of **5** (CD_3OD).

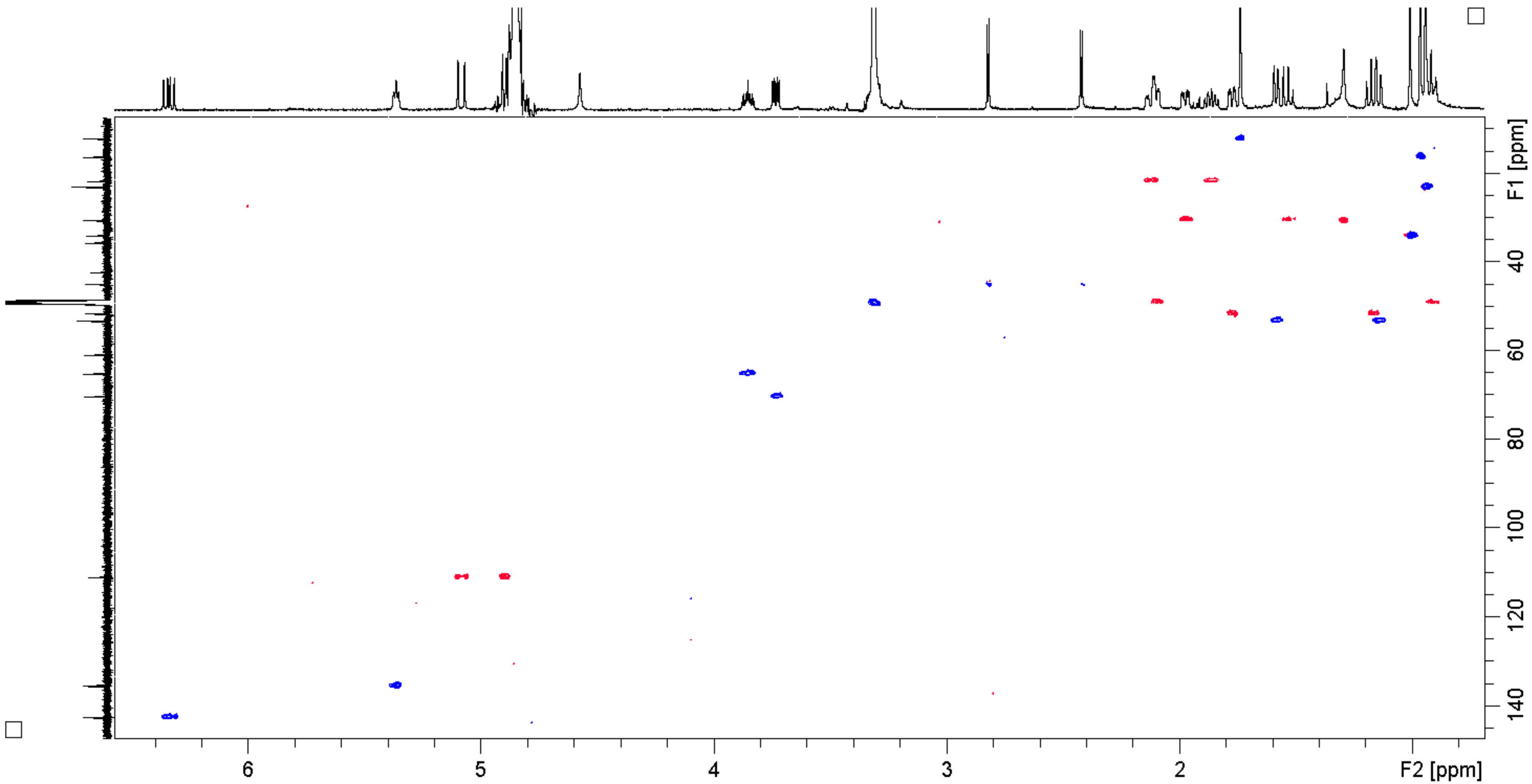

Figure S48. HSQC NMR spectrum of **5** (CD_3OD).

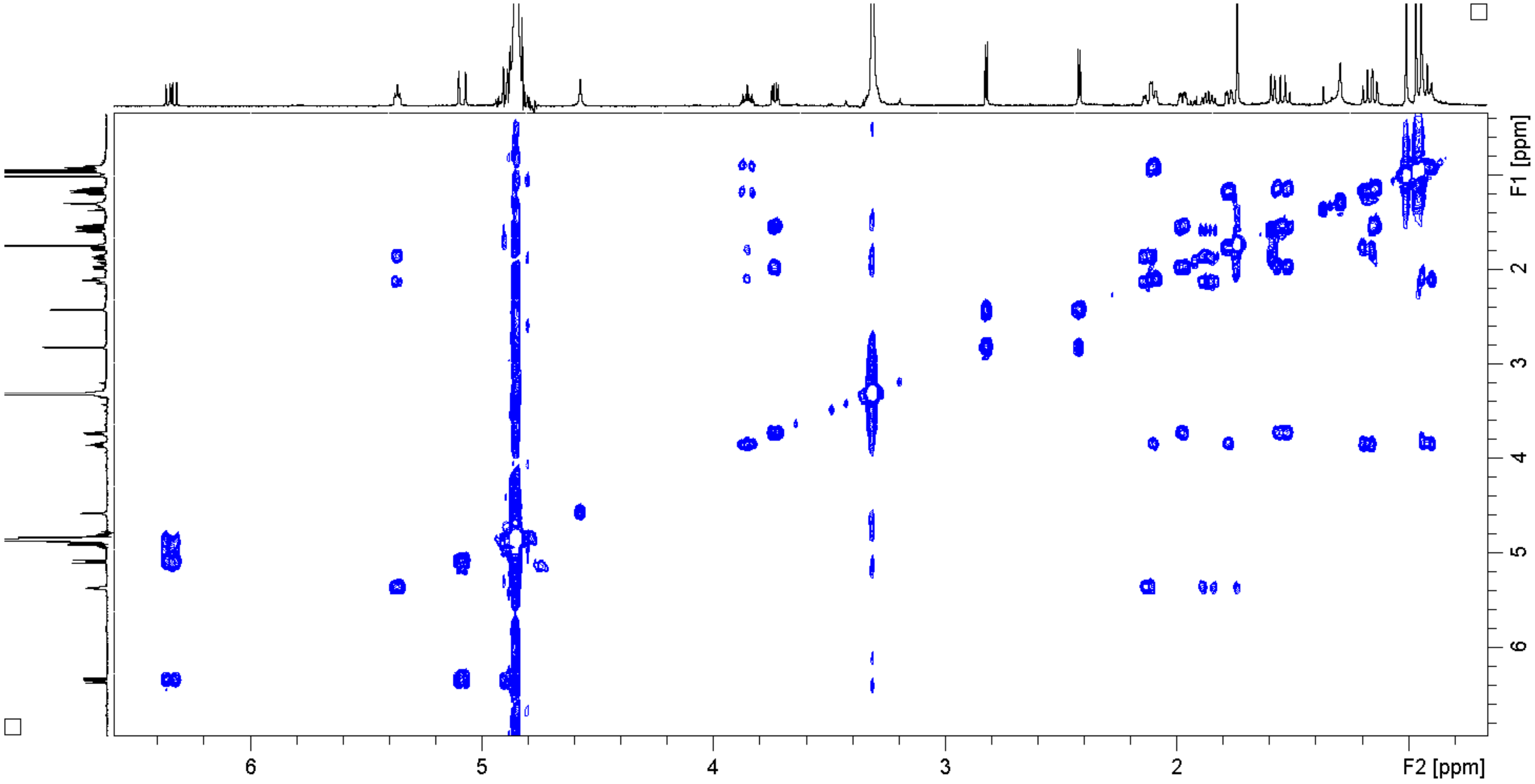

Figure S49. ^{1}H-^{1}H COSY NMR spectrum of **5** (CD_3OD).

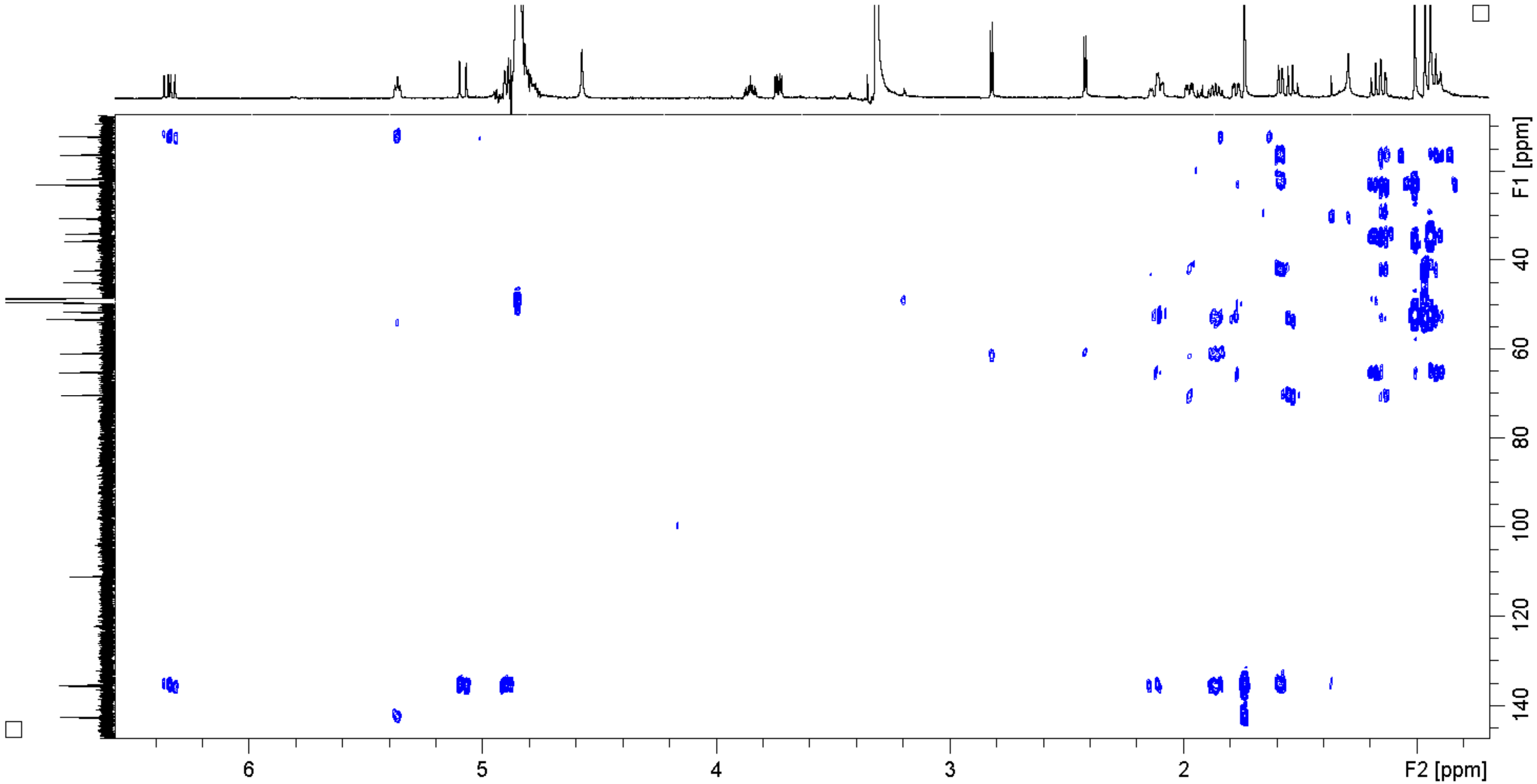

Figure S50. HMBC NMR spectrum of **5** (CD_3OD).

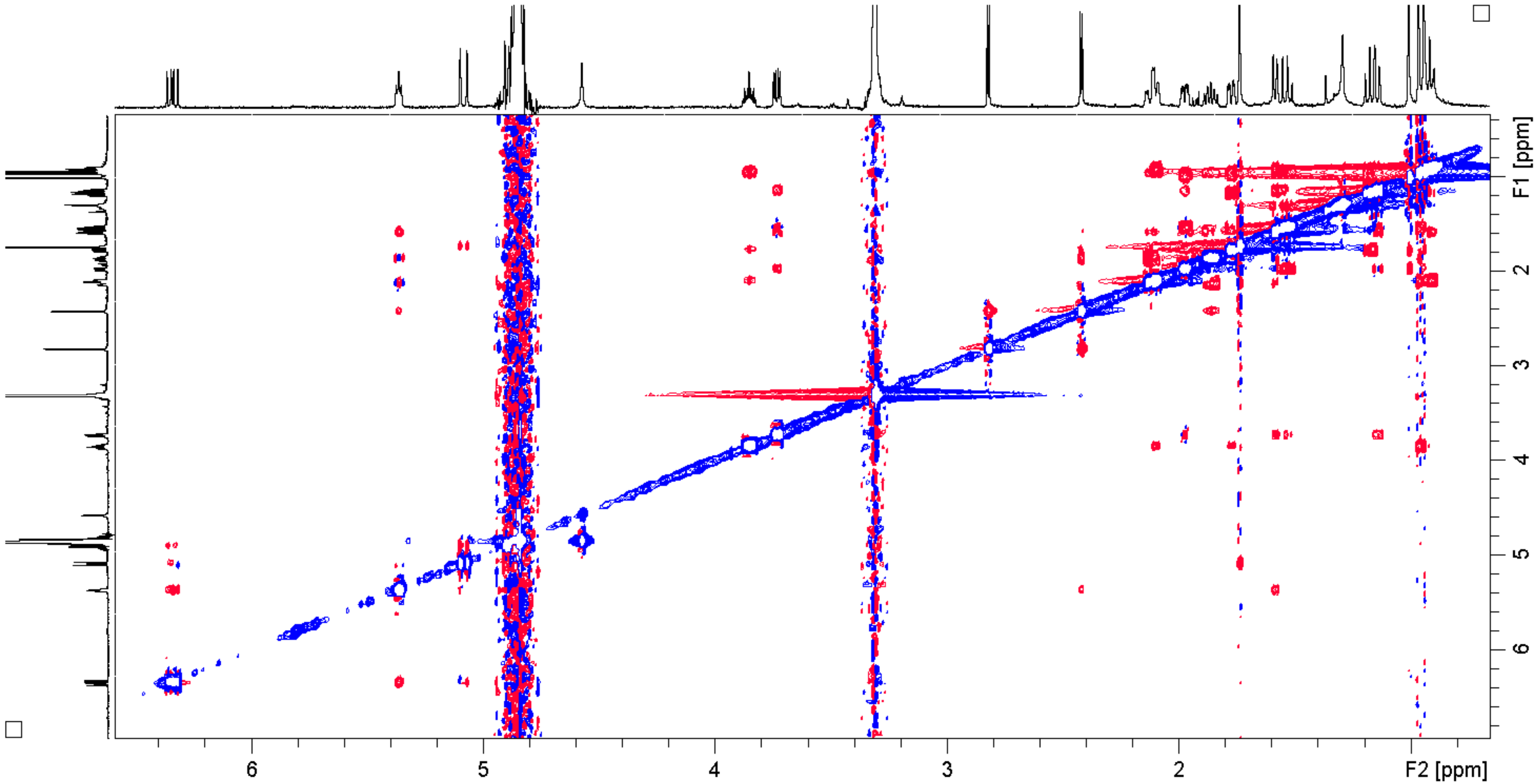

Figure S51. NOESY NMR spectrum of **5** (CD_3OD).

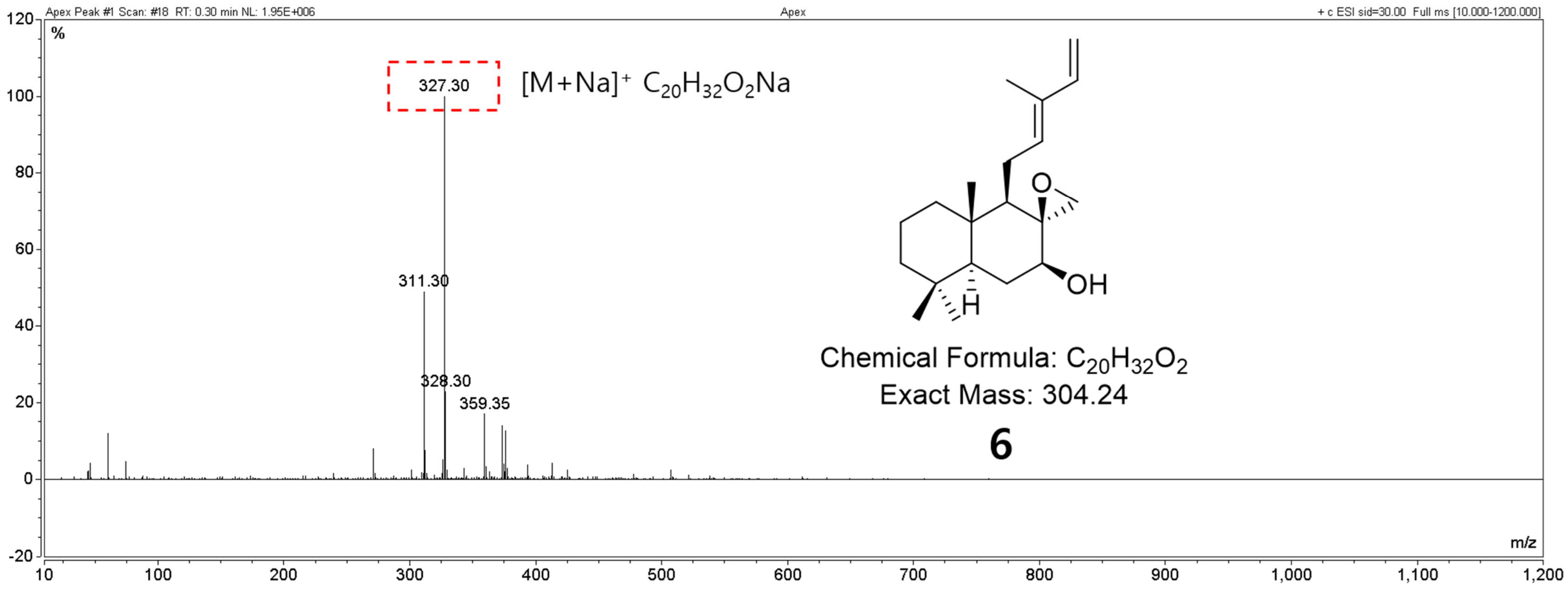

Figure S52. LRESIMS data of **6**.

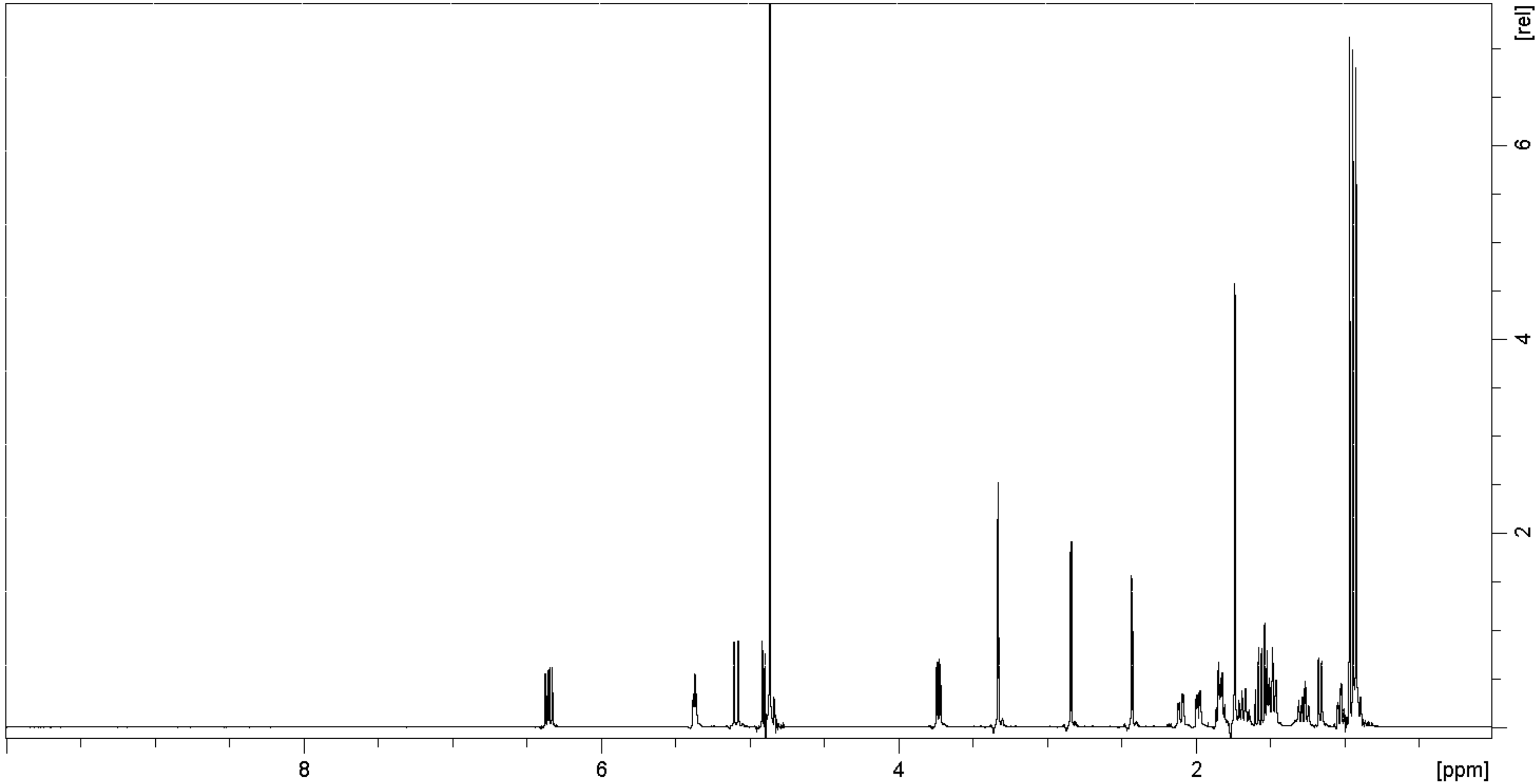

Figure S53. ^{1}H NMR spectrum of **6** (CD_3OD).

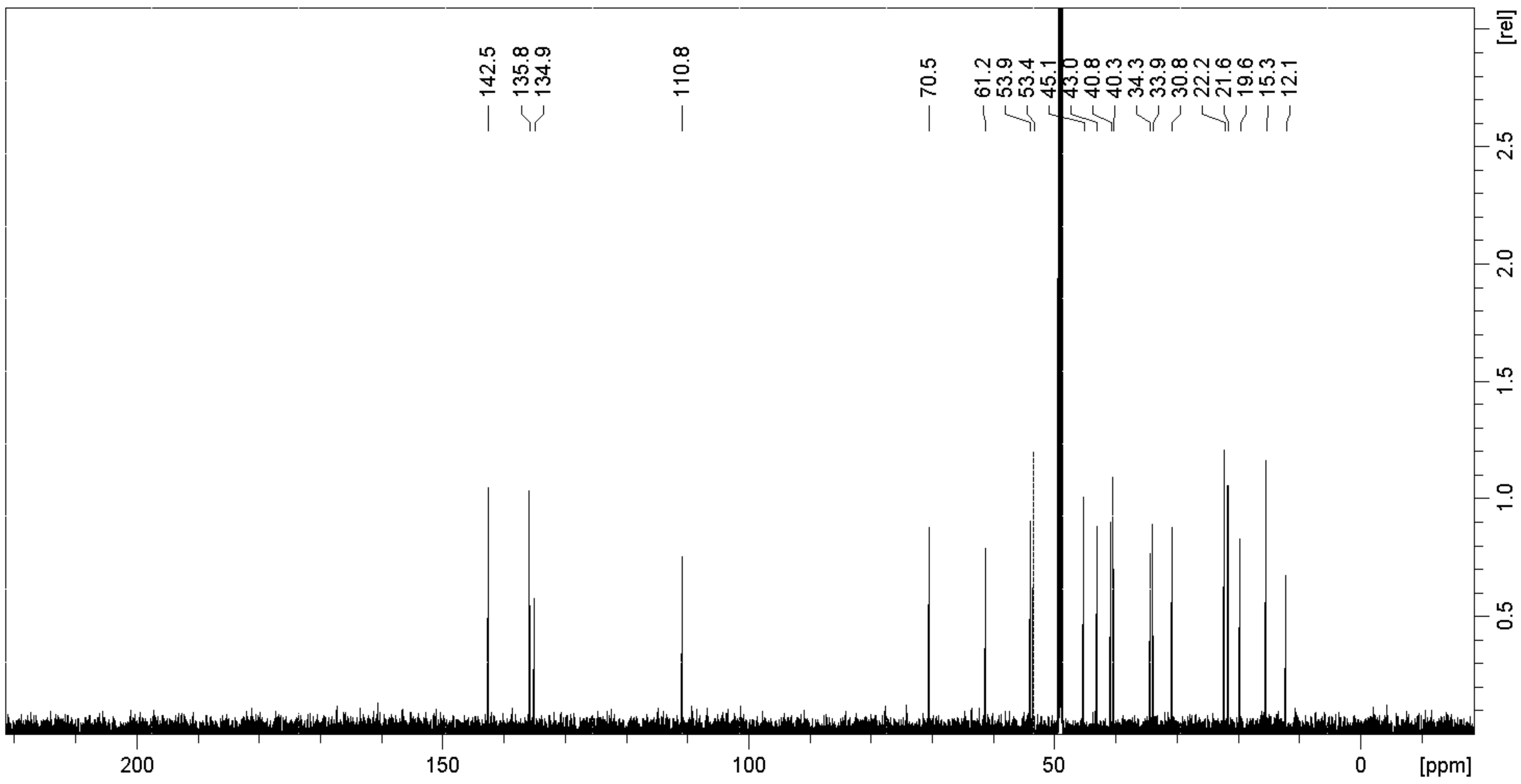

Figure S54. ^{13}C NMR spectrum of **6** (CD_3OD).

HL-60	1		2		4		Doxorubicin	
(μM)	MEAN	SD	MEAN	SD	MEAN	SD	MEAN	SD
0.1	97.7	1.9	80.0	3.0	93.3	1.3	1.6	0.2
0.03	102.6	2.8	95.1	0.7	92.2	2.1	30.0	3.3
0.01	99.6	1.6	99.1	4.3	94.9	2.4	75.2	3.6
0.003	96.8	1.9	98.5	2.6	94.7	2.1	90.5	4.6
0.001	94.6	2.4	96.2	1.1	84.6	4.2	85.8	2.7
IC50	>30		>30		>30		0.018	

Raji	1		2		4		Doxorubicin	
(μM)	MEAN	SD	MEAN	SD	MEAN	SD	MEAN	SD
0.1	27.4	2.6	0.1	0.1	30.1	6.4	0.8	0.2
0.03	67.7	5.5	10.1	5.3	73.6	8.6	22.0	2.5
0.01	86.5	7.4	64.5	7.3	79.1	9.3	52.5	1.2
0.003	92.9	7.5	79.3	6.5	92.5	4.0	85.7	3.2
0.001	102.4	6.4	82.4	8.0	94.3	8.2	88.9	9.9
IC50	15.520		3.406		17.110		0.011	

WSU-DLCL2	1		2		4		Doxorubicin	
(μM)	MEAN	SD	MEAN	SD	MEAN	SD	MEAN	SD
0.1	50.5	4.8	0.0	0.0	8.1	2.8	0.4	0.1
0.03	85.0	4.0	8.7	2.6	75.2	4.0	4.0	0.8
0.01	93.7	6.0	43.1	7.8	82.1	8.0	20.9	0.6
0.003	100.1	6.4	56.4	3.7	89.2	3.9	90.1	8.9
0.001	101.2	7.7	66.5	8.0	92.1	3.6	94.8	8.4
IC50	>30(30.7)		1.182		14.170		0.006	

NALM6 C. G5	1		2		4		Doxorubicin	
(μM)	MEAN	SD	MEAN	SD	MEAN	SD	MEAN	SD
0.1	86.3	5.2	39.3	5.7	100.2	7.6	0.1	0.0
0.03	91.9	3.3	77.7	4.1	91.9	4.1	-0.1	0.0
0.01	102.8	2.3	88.6	1.8	94.3	5.9	2.5	1.1
0.003	110.6	0.6	99.5	2.8	96.0	4.9	64.0	4.7
0.001	99.8	3.9	107.0	2.6	87.4	6.2	82.8	6.8
IC50	>30		22.460		>30		0.003	

K562	1		2		4		Doxorubicin	
(μM)	MEAN	SD	MEAN	SD	MEAN	SD	MEAN	SD
0.1	86.2	1.6	53.4	2.2	97.7	1.2	3.8	0.1
0.03	96.9	1.8	84.3	2.8	86.8	4.6	4.6	0.9
0.01	109.8	5.2	98.4	3.9	91.6	2.6	31.4	1.3
0.003	113.3	3.4	109.7	3.0	99.1	0.7	56.9	2.5
0.001	114.0	2.6	114.3	1.1	98.8	1.5	65.7	4.3
IC50	>30		>30		>30		0.003	

RPMI-8402	1		2		4		Doxorubicin	
(μM)	MEAN	SD	MEAN	SD	MEAN	SD	MEAN	SD
0.1	83.3	3.4	56.3	6.8	91.9	4.4	1.2	0.4
0.03	86.2	3.9	74.0	6.1	85.9	4.2	13.5	0.6
0.01	84.5	5.5	91.5	6.6	82.3	5.4	58.1	5.3
0.003	96.3	4.5	94.9	8.8	80.6	8.4	68.7	5.2
0.001	104.4	6.4	94.3	4.6	85.2	6.8	77.2	2.0
IC50	>30		>30		>30		0.008	

U266	1		2		4		Doxorubicin	
(μM)	MEAN	SD	MEAN	SD	MEAN	SD	MEAN	SD
0.1	101.0	4.2	83.6	1.6	99.0	7.8	17.2	1.4
0.03	96.3	3.3	92.7	5.4	89.3	4.5	68.1	4.2
0.01	99.2	4.1	96.4	1.9	92.8	4.0	84.4	3.5
0.003	100.3	2.0	100.1	4.5	93.4	4.5	88.9	4.4
0.001	97.5	4.6	97.7	2.8	90.6	1.9	86.8	4.5
IC50	>30		>30		>30		0.042	

RPMI-1788	1		2		4		Doxorubicinl	
(μM)	MEAN	SD	MEAN	SD	MEAN	SD	MEAN	SD
0.1	89.9	4.9	60.9	4.1	97.2	9.2	3.2	0.4
0.03	95.2	1.4	88.3	4.6	92.1	6.0	14.0	0.9
0.01	99.1	5.0	94.2	1.2	96.0	4.0	38.6	6.0
0.003	99.0	2.2	94.0	1.7	94.5	3.1	85.7	5.0
0.001	96.1	1.8	96.6	7.2	89.4	2.6	99.9	2.8
IC50	>30		>30		>30		0.008	

ACHN	1		2		4		Adriamycin		
(μM)	MEAN	SD	MEAN	SD	MEAN	SD	(μM)	MEAN	SD
30	72.06	6.94	72.00	5.82	81.94	4.42	3	−13.42	2.41
10	74.24	5.77	83.14	6.51	85.65	5.73	1	−4.90	6.19
3	86.68	7.11	89.39	2.48	90.98	5.35	0.3	12.82	5.58
1	87.08	6.98	93.12	5.49	95.41	6.17	0.1	30.04	2.04
0.3	94.02	3.03	99.14	3.93	98.72	1.13	0.03	92.15	7.02
GI50	>30		>30		>30		GI50	0.074	

MDA-MB-231	1		2		4		Adriamycin		
(μM)	MEAN	SD	MEAN	SD	MEAN	SD	(uM)	MEAN	SD
30	77.94	4.06	72.56	4.31	81.81	6.11	3	−11.01	7.44
10	80.43	2.07	77.83	4.53	88.19	7.42	1	−2.06	4.27
3	83.87	5.13	85.05	6.12	89.22	5.68	0.3	11.59	7.77
1	91.94	4.20	96.03	4.16	95.73	4.98	0.1	30.89	5.00
0.3	96.84	3.50	98.84	4.00	98.22	4.02	0.03	94.68	5.94
GI50	>30		>30		>30		GI50	0.076	

PC-3	1		2		4		Adriamycin		
(μM)	MEAN	SD	MEAN	SD	MEAN	SD	(μM)	MEAN	SD
30	85.23	6.01	80.76	5.23	71.11	1.31	3	−10.58	3.74
10	86.14	3.39	82.94	7.49	76.59	7.90	1	−7.86	1.71
3	90.37	2.47	89.14	4.34	87.52	6.75	0.3	16.13	9.96
1	94.14	3.81	91.13	2.52	93.34	2.66	0.1	27.40	3.53
0.3	98.40	3.16	96.25	4.32	98.22	3.40	0.03	95.10	7.57
GI50	>30		>30		>30		GI50	0.073	

NUGC-3	1		2		4		Adriamycin		
(μM)	MEAN	SD	MEAN	SD	MEAN	SD	(uM)	MEAN	SD
30	75.75	6.55	76.07	2.78	71.34	4.23	3	−10.63	2.56
10	79.27	9.65	82.67	5.34	78.68	0.19	1	−5.42	8.59
3	85.43	7.30	88.71	6.11	89.75	6.00	0.3	18.29	9.25
1	92.73	2.49	92.48	6.47	93.55	5.00	0.1	43.64	7.94
0.3	94.27	2.49	98.26	4.35	97.32	2.77	0.03	93.91	5.80
GI50	>30		>30		>30		GI50	0.095	

NCI-H23	1		2		4		Adriamycin		
(μM)	MEAN	SD	MEAN	SD	MEAN	SD	(μM)	MEAN	SD
30	73.68	5.34	72.20	6.69	78.62	7.31	3	−9.73	5.18
10	83.08	4.95	87.93	2.40	89.75	5.63	1	−6.45	2.12
3	87.02	4.74	88.07	0.81	90.41	3.68	0.3	16.90	8.45
1	95.45	7.56	96.23	4.02	92.36	6.61	0.1	27.58	2.13
0.3	100.36	2.31	98.57	6.77	94.13	5.84	0.03	96.57	7.31
GI50	>30		>30		>30		GI50	0.074	

HCT-15	1		2		4		Adriamycin		
(μM)	MEAN	SD	MEAN	SD	MEAN	SD	(uM)	MEAN	SD
30	75.84	8.94	77.84	4.60	75.49	6.52	3	−7.03	2.43
10	82.36	5.39	88.67	3.30	81.85	7.82	1	−3.82	3.78
3	84.01	6.82	91.35	2.47	84.38	0.68	0.3	19.05	4.66
1	90.80	0.61	92.46	3.51	96.86	1.49	0.1	35.19	2.10
0.3	96.50	3.57	100.50	4.90	98.37	4.05	0.03	98.35	3.87
GI50	>30		>30		>30		GI50	0.084	

MRC-9	1		2		4		Adriamycin		
(μM)	MEAN	SD	MEAN	SD	MEAN	SD	(μM)	MEAN	SD
30	80.62	6.81	85.72	3.97	74.81	8.01	3	−7.72	3.86
10	85.93	4.24	88.22	5.74	89.10	4.65	1	−4.38	3.80
3	88.28	5.42	90.65	5.28	91.80	7.86	0.3	19.26	6.64
1	97.15	4.12	95.54	4.32	93.31	3.99	0.1	39.69	8.11
0.3	100.00	1.62	97.15	5.80	96.06	3.46	0.03	94.36	4.23
GI50	>30		>30		>30		GI50	0.090	

Table S1. Results of the cytotoxicity test of **1**, **2**, and **4**.
